# Emestrin-type epipolythiodioxopiperazines from *Aspergillus nidulans* with cytotoxic activities by regulating PI3K/AKT and mitochondrial apoptotic pathways

**DOI:** 10.1007/s13659-025-00498-8

**Published:** 2025-03-10

**Authors:** Pengkun Li, Qin Li, Aimin Fu, Yang Xiao, Chunmei Chen, Hucheng Zhu, Changxing Qi, Wei Wei, Yuan Zhou, Yonghui Zhang

**Affiliations:** 1 Hubei Key Laboratory of Natural Medicinal Chemistry and Resource Evaluation, School of Pharmacy, Tongji Medical College, Huazhong University of Science and Technology, Wuhan, 430030 People’s Republic of China; 2 China National Center for Biotechnology Development, Beijing, 100039 People’s Republic of China

**Keywords:** *Aspergillus nidulans*, Epipolythiodioxopiperazines, Thioethanothio bridge, Structural elucidation, Cytotoxicity

## Abstract

Five novel emestrin-type epipolythiodioxopiperazines (ETPs), prenylemestrins C−G (**1**–**5**), along with two known ETPs, prenylemestrin A (**6**) and prenylemestrin B (**7**), were obtained from *Aspergillus nidulans*. Their structures were characterized by spectroscopic data, X-ray crystallographic data, ECD comparisons and calculations. Prenylemestrins C−G (**1** − **5**) represent a rare class of ETPs, characterized by a 2,5-dithia-7,9-diazabicyclo[4.2.2]decane-8,10-dione core involving a hemiterpene moiety. Notably, compound **6** exhibited moderate cytotoxicity, inducing G2/M cell cycle arrest and apoptosis of L1210 cells by regulating the PI3K/AKT signaling pathway and mitochondrial apoptotic mechanisms.

**Graphical Abstract:**

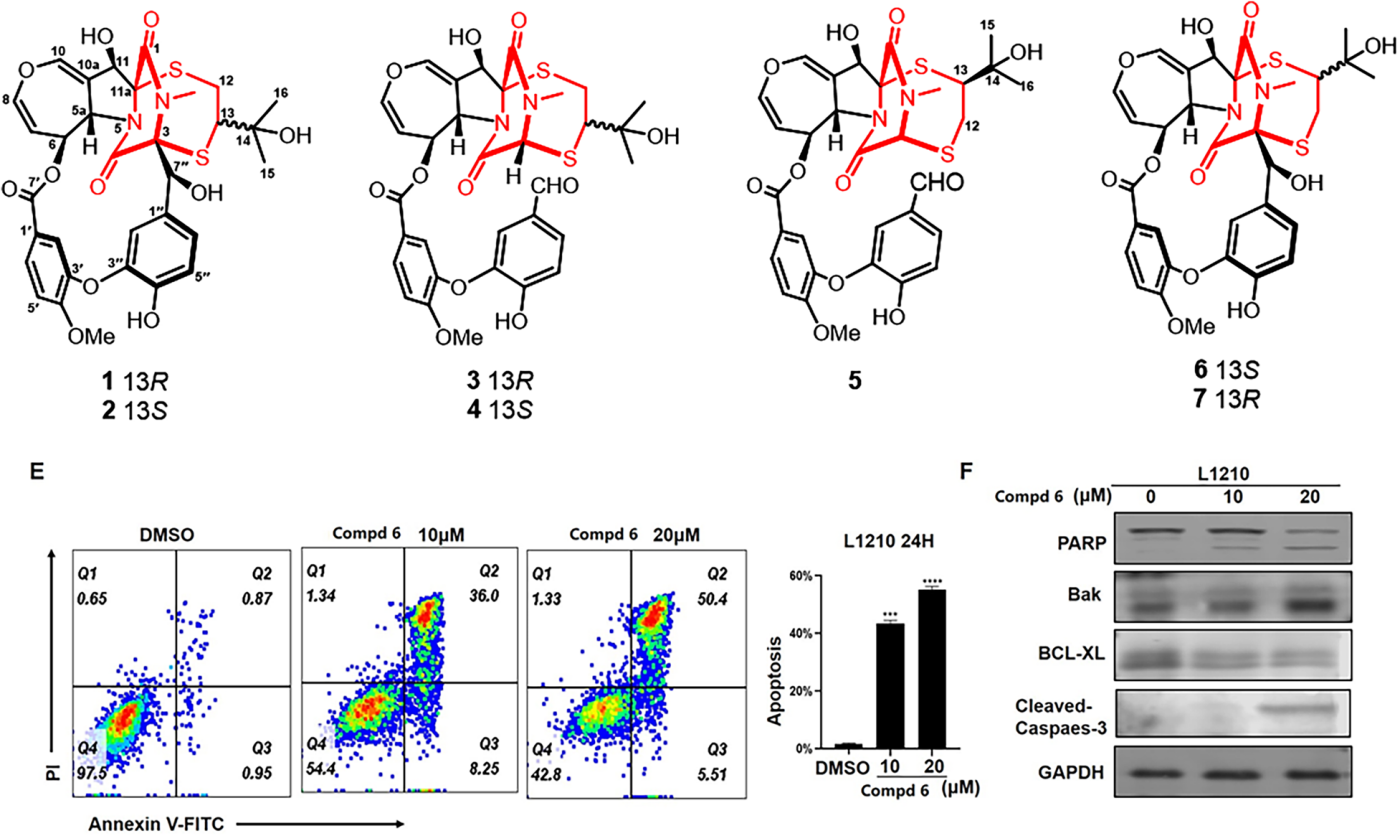

**Supplementary Information:**

The online version contains supplementary material available at 10.1007/s13659-025-00498-8.

## Introduction

Fungal natural products are a crucial source for drug discovery, serving as a rich reservoir for novel bioactive compounds [1–3]. Their fascinating structural diversity and biological activities hold out the ideal choices for the development of novel agents, and they also help discover novel structures, providing effective agents for the treatment of various human diseases [2, 4–6]. Epipolythiodioxopiperazines (ETPs) represent a distinct class of fungal metabolites, characterized by a di- or polysulfide-bridged dioxopiperazine ring, and derived from two amino acids. These compounds are renowned for broad-spectrum of bioactivities, including cytotoxicity against tumor cells, antibacterial function, antiviral, immunologic suppression, and anti-inflammatory action [7–10]. Based on the structure of the core ETP moiety and the modifications, emestrins are a notable group of monomeric ETPs, distinguished by a dihydrooxepino[4,3-*b*]pyrrole core and 15-membered macrolide [11]. Emestrin, first obtained from *Emericella striata* in 1985, demonstrated antifungal activity and cytotoxicity *via* DNA fragmentation in HL-60 cells [12–15]. To date, numerous emestrin-type ETPs have been identified, with their structural uniqueness and promising bioactivities drawing increasing scientific interest [16–22]. For instance, secoemestrin C exhibits potent anti-cancer activity in GEM-resistant and GEM-sensitive pancreatic adenocarcinoma (PAAD) cells, inducing mitochondria-mediated apoptosis and causing severe endoplasmic reticulum damage [23, 24].

**Fig. 1:**
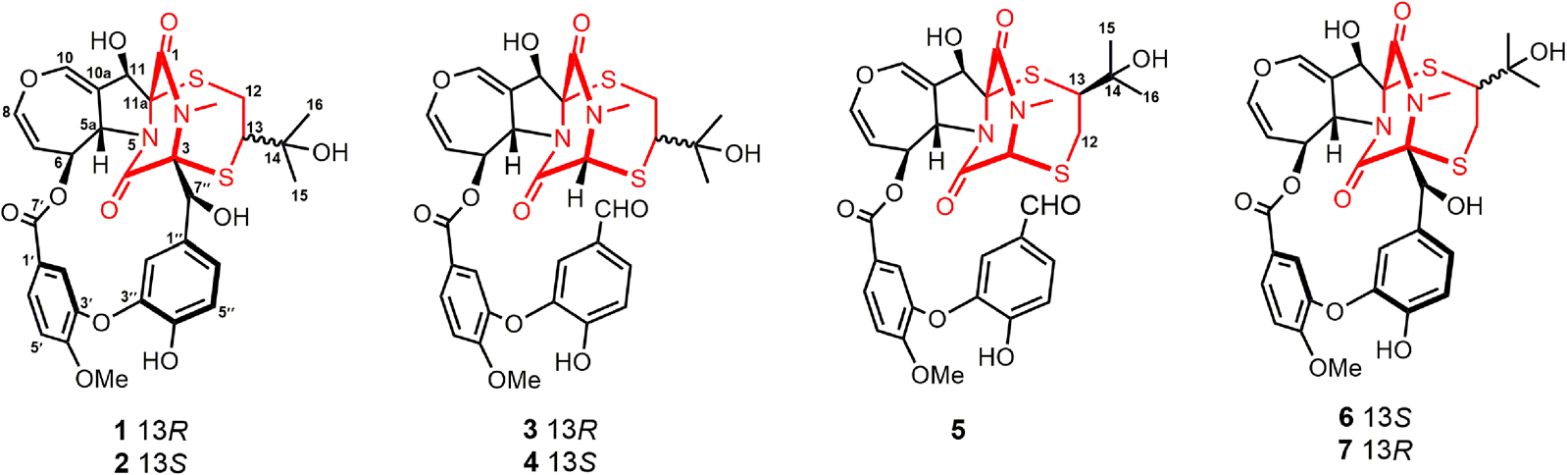
Chemical structures of **1**−**7**

Previously, we identified four emestrin hybrid polymers, asperemestrins A–D, and a highly productive ETP, secoemestrin C, which exhibited notable immunosuppressive effect, from *Aspergillus nidulans* [19, 25]. Recently, we found that the first cysteine-retained emestrin, nidustrin A, features a unique sulfur-containing 18-membered macrocyclic lactone [26]. Building on this work, our ongoing efforts to explore bioactive ETPs have led to the discovery of prenylemestrins C−G (**1** − **5**). These compounds are distinguished by a 2,5-dithia-7,9-diazabicyclo[4.2.2]decane-8,10-dione core involving a hemiterpene moiety (Fig. 1). In this study, the isolation, structural elucidation, and biological activities of these compounds were described in detail.

## Results and discussion

Prenylemestrin C (**1**) was isolated as a white powder with the molecular formula C_32_H_32_N_2_O_11_S_2_, based on the HRESIMS spectrum with an ion peak at *m/z* 707.1345 ([M + Na]^+^, calcd. for 707.1345), indicating 18 degrees of unsaturation. The IR spectrum revealed the presence of hydroxyl (3419 cm^−1^), carbonyl (1691 and 1662 cm^−1^), and aromatic ring (1513 cm^−1^) functionalities. The ^1^H NMR data of **1** recorded in CD_3_OD (Table 1) showed signals corresponding to two 1,3,4-trisubstituted benzene rings protons at *δ*_H_ 7.98 (d, *J* = 2.0 Hz), 7.81 (dd, *J* = 8.6, 2.0 Hz), and 7.19 (d, *J* = 8.6 Hz), and *δ*_H_ 8.42 (d, *J* = 2.3 Hz), 7.28 (dd, *J* = 8.6, 2.3 Hz), and 6.90 (d, *J* = 8.6 Hz), seven methines including three olefinic [*δ*_H_ 6.92 (d, *J* = 2.4 Hz), 6.42 (dd, *J* = 8.1, 2.4 Hz), and 5.01 (dd, *J* = 8.1, 2.3 Hz)] and three oxymethines [*δ*_H_ 5.49 (dt, *J* = 8.3, 2.3 Hz), 5.12 (s), and 4.72 (s)], one methylene [*δ*_H_ 3.46 (dd, *J* = 16.5, 1.8 Hz) and 2.89 (dd, *J* = 16.5, 5.3 Hz)], a methoxy group (*δ*_H_ 3.99), an *N*-methyl (*δ*_H_ 3.36), and two singlet methyls (*δ*_H_ 0.80, 0.68). The ^13^C NMR and DEPT spectroscopic data of **1** recorded in CD_3_OD displayed 32 carbon signals (Table 2), including three carbonyls (*δ*_C_ 167.2, 167.1, 161.5), 10 nonprotonated carbons (seven olefinic and three oxygenated *sp*^3^ at *δ*_C_ 78.0, 76.5, 73.8), 14 methines (nine olefinic and five *sp*^3^), a methylene (*δ*_C_ 35.3), and four methyls (*δ*_C_ 56.8, 29.0, 26.1, 28.4).

**Table 1:** ^1^H NMR Spectroscopic Data (400 MHz; *δ* in ppm, *J* in Hz) for Compounds **1**–**5**

No	**1**^*a*^	**1**^*b*^	**2**^*b*^	**3**^*c*^	**4**^*b*^	**5**^*c*^
3				4.96, s	4.97, s	4.83, s
5a	5.07, dd (8.3, 2.5)	5.30, dd (8.3, 2.5)	5.42, overlapped	5.44, dd (8.0, 2.5)	5.31, dd (8.3, 2.3)	5.44, dd (8.2, 2.5)
6	5.29, dt (8.3, 2.4)	5.49, dt (8.3, 2.3)	5.42, overlapped	6.02, dt (8.0, 2.3)	5.58, dt (8.3, 2.3)	5.73, dt (8.2, 2.2)
7	5.00, dd (8.0, 2.3)	5.01, dd (8.1, 2.3)	4.96, dd (8.2, 1.9)	4.88, dd (8.2, 2.1)	4.90, dd (8.2, 2.2)	4.88, dd (8.2, 2.1)
8	6.47, dd (8.0, 2.4)	6.42, dd (8.1, 2.4)	6.41, dd (8.2, 2.2)	6.38, dd (8.2, 2.4)	6.41, dd (8.2, 2.4)	6.34, dd (8.3, 2.4)
10	6.99, d (2.5)	6.92, d (2.4)	6.89, d (2.3)	6.89, d (2.4)	6.91, d (2.4)	6.85, d (2.5)
11	4.58, d (3.8)	4.72, s	4.61, s	4.80, s	4.61, s	4.73, brs
12	2.68, dd (16.4, 5.2) 3.42, dd (16.4, 1.5)	2.89, dd (16.5, 5.3) 3.46, dd (16.5, 1.8)	2.76, dd (15.5, 5.4) 3.35, overlapped	3.07, dd (16.2, 5.3) 3.34, dd (16.2, 1.8)	2.79, dd (15.9, 5.3) 3.29, dd (15.9, 2.1)	2.70, dd (17.0, 5.1) 2.92, dd (17.0, 2.0)
13	3.16, dd (5.2, 1.5)	3.33, overlapped	2.57, dd (5.5, 1.8)	3.38, d (5.3)	2.67, dd (5.3, 1.8)	3.01, dd (5.1, 2.0)
15	0.56, s	0.68, s	0.77, s	0.93, s	0.78, s	1.17, s
16	0.67, s	0.80, s	1.22, s	1.01, s	1.22, s	1.20, s
2ʹ	7.66, d (2.0)	7.98, d (2.0)	8.09, d (2.1)	8.10, d (2.1)	7.75, d (2.1)	8.10, d (2.1)
5ʹ	7.18, d (9.1)	7.19, d (8.6)	7.20, d (8.7)	7.08, d (8.7)	7.18, d (8.7)	7.09, d (8.7)
6ʹ	7.67, dd (9.1, 2.0)	7.81, dd (8.6, 2.0)	7.85, dd (8.7, 2.1)	8.14, dd (8.7, 2.1)	8.02, dd (8.7, 2.1)	8.11, dd (8.7, 2.1)
2ʹʹ	8.17, d (2.2)	8.42, d (2.3)	8.19, d (2.3)	7.39, d (1.8)	7.12, d (2.0)	7.41, d (1.8)
5ʹʹ	6.87, d (8.6)	6.90, d (8.6)	6.88, d (8.4)	7.14, d (8.2)	6.88, d (8.4)	7.14, d (8.2)
6ʹʹ	7.22, dd (8.6, 2.2)	7.28, dd (8.6, 2.3)	7.16, dd (8.4, 2.3)	7.54, dd (8.2, 1.8)	7.47, dd (8.4, 2.0)	7.53, dd (8.2, 1.8)
7ʹʹ	4.98, d (8.0)	5.12, s	5.29, s	9.75, s	9.51, s	9.75, s
N-Me	3.18, s	3.36, s	3.31, s	3.16, s	3.06, s	3.10, s
O-Me	3.89, s	3.99, s	3.98, s	3.89, s	3.90, s	3.91, s
OH-11	6.07, d (3.8)					
OH-14	4.70, s					
OH-7ʹʹ	6.25, d (8.0)					

^*a*^Recorded in DMSO-*d*_6_; ^*b*^Recorded in CD_3_OD; ^*c*^Recorded in CDCl_3_

**Table 2:** ^13^C NMR Spectroscopic Data (100 MHz; *δ* in ppm) for Compounds **1**–**5**

No	**1**^*a*^	**1**^*b*^	**2**^*b*^	**3**^*c*^	**4**^*b*^	**5**^*c*^
1	164.2	167.1	170.4	164.6	169.1	168.5
3	74.9	76.5	76.3	65.7	64.5	63.6
4	159.4	161.5	165.4	160.6	165.5	163.9
5a	57.6	59.4	59.6	59.9	60.4	60.1
6	74.7	74.5	75.2	73.6	74.7	73.3
7	108.7	109.6	108.4	107.6	108.5	106.8
8	139.4	140.4	140.5	139.7	140.4	139.7
10	139.6	141.7	142.0	142.8	142.5	142.8
10a	111.7	112.3	113.7	110.0	114.2	111.0
11	78.2	80.6	80.6	79.5	79.7	79.9
11a	76.1	78.0	75.3	76.9	76.0	74.6
12	33.9	35.3	35.7	35.7	35.5	33.3
13	57.8	59.8	61.0	57.6	58.5	60.9
14	71.9	73.8	73.7	73.3	73.5	73.0
15	25.7	26.1	24.8	27.2	24.7	27.2
16	27.8	28.4	29.3	24.8	29.4	26.0
1′	121.0	122.9	122.9	122.7	123.3	122.4
2′	115.7	118.8	122.5	124.8	123.0	124.7
3′	147.2	148.3	146.6	144.0	146.4	144.0
4′	153.6	155.9	156.7	155.9	157.0	156.0
5′	112.1	113.3	113.3	112.4	113.1	112.4
6′	125.0	127.2	127.9	129.8	129.1	129.9
7′	165.1	167.2	167.2	165.7	167.2	166.4
1′′	134.1	134.8	132.8	130.0	126.5	130.0
2′′	120.5	121.6	120.2	116.5	118.4	116.4
3′′	139.2	141.9	143.2	146.1	148.9	146.1
4′′	146.6	148.0	147.7	152.9	148.5	153.1
5′′	116.0	116.9	116.8	116.2	119.7	116.2
6′′	122.9	124.2	125.0	129.2	131.0	129.4
7′′	73.0	75.4	76.9	190.9	191.8	191.0
N-Me	28.3	29.0	28.7	32.0	32.0	32.0
O-Me	55.9	56.8	56.8	56.3	56.6	56.3

^*a*^Recorded in DMSO-*d*_6_; ^*b*^Recorded in CD_3_OD; ^*c*^Recorded in CDCl_3_

**Fig. 2:**
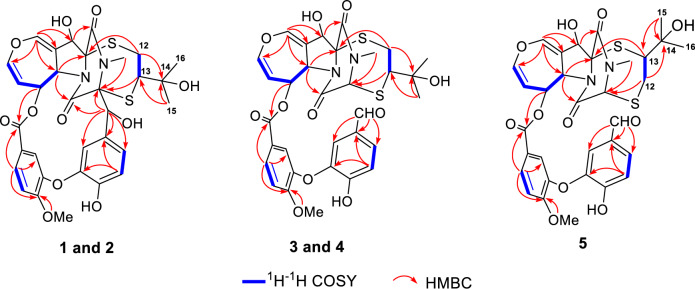
Key ^1^H−^1^H COSY and HMBC correlations of compounds **1**−**5**

The planar structure of compound **1** was elucidated through 2D NMR spectral analysis. The independent spin system of H-5a/H-6/H-7/H-8, along with HMBC correlations of H-10/C-5a, C-8, and C-10a, confirmed the presence of a 6,7-dihydrooxepine ring. Further HMBC spectrum showed correlations from H-11 to C-5a, C-10a, C-11a, and C-1, as well as from H-5a to C-4 and from the N-methyl protons to C-1 and C-3. These findings established that the 6,7-dihydrooxepine ring was fused to a dioxopiperazine moiety via an 11-hydroxypyrrolidine ring. Additionally, a 3′-oxygen-4′-methoxy-benzoate fragment and a 3′′-oxygen-4′′-hydroxybenzyl unit were identified as being connected to the 6,7-dihydrooxepine ring at C-6 and the dioxopiperazine ring at C-3, respectively. This connectivity was supported by the HMBC correlations from H-6 to C-7′ and from H-7′′ to C-3, C-4, C-1′′, C-2′′, and C-6′′. Moreover, the ^1^H−^1^H COSY correlation of H_2_-12/H-13, combined with the HMBC cross-peak from H_2_-12 to C-11a, H-13 to C-3, H_3_-15/H_3_-16 to C-13 and C-14, confirmed the attachment of an isopentan-3-ol fragment to C-11a and C-3 through sulfur atoms. Consequently, compound **1** was deduced to feature an epipolythiodioxopiperazine skeleton with a thioethanothio bridge, closely resembling the planar structure with prenylemestrins A and B (**6** and **7**) [17]. A detailed comparison of their ^1^H and ^13^C NMR data revealed that the key difference lies in the 2,5-dithia-7,9-diazabicyclo[4.2.2]decane-8,10-dione core. HMBC correlations (Fig. 2) from H_2_-12 to C-11a and from H-13 to C-3 confirmed that C-12 and C-13 were linked to C-11a and C-3, respectively. With the hemiterpene moiety switched, **1** can also be interpreted as a migration of the 2-hydroxyisopropyl group.

**Fig. 3:**
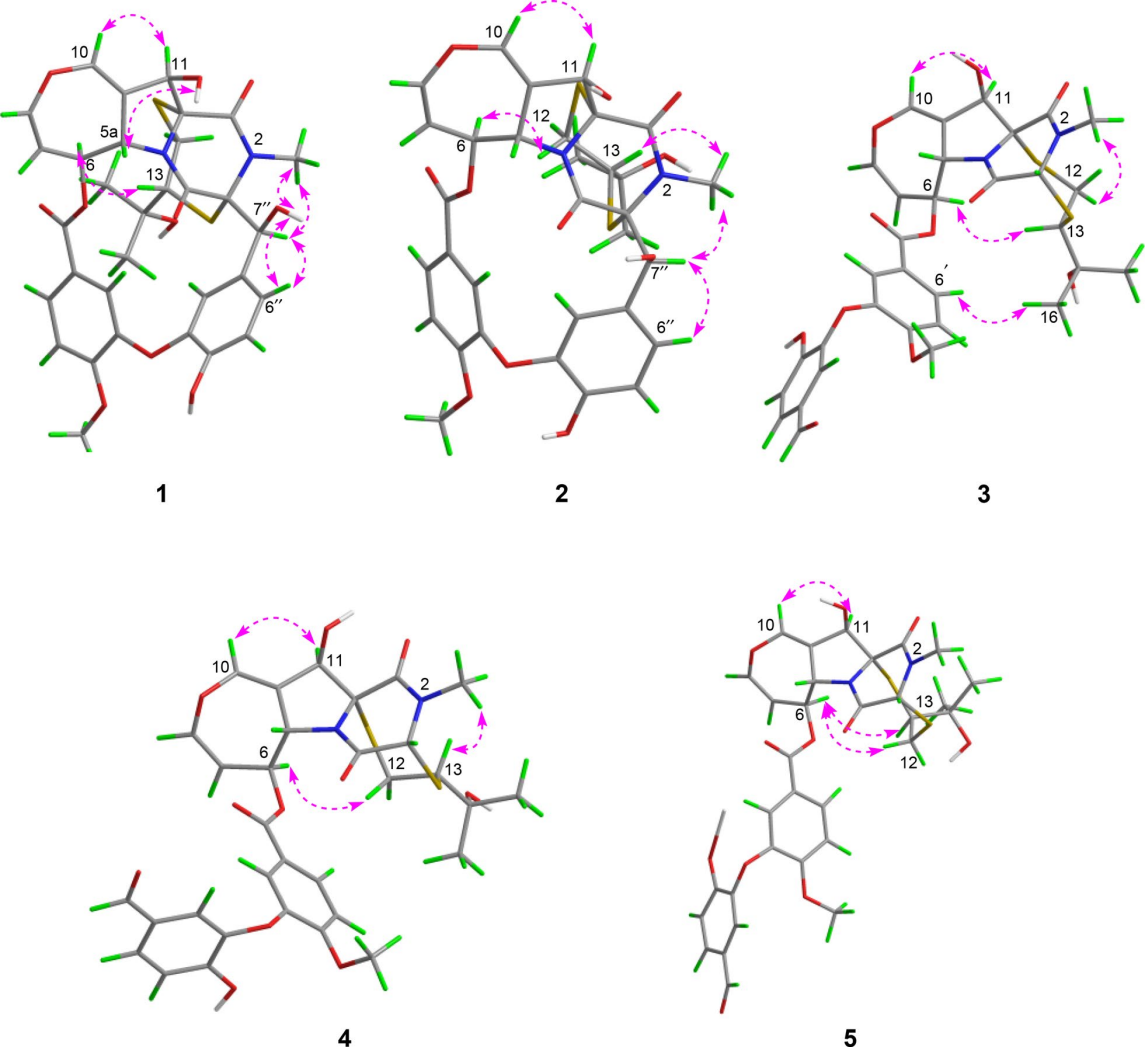
Key NOESY correlations of compounds **1**−**5**

To determine the relative configuration of compound **1**, NMR data were recorded in DMSO-*d*_6_ (Tables 1 and 2). The *trans*-orientation of H-5a and H-6 was confirmed by the large coupling constant (*J* = 8.3 Hz) observed between those protons [21]. The NOESY correlation between H-5a and OH-11 indicated that H-11 and H-6 were on the same side, tentatively assigned as *α* orientation [17]. Furthermore, NOESY correlations (Fig. 3) of H-6/H-13, H-2′/Me-15/Me-16, H-2″/Me-15/Me-16, suggested that the configuration of C-13 should be R^*^. Accordingly, the C-3 − S and C-11a − S bonds were deduced to be *α*-oriented. Additionally, NOESY interactions of N-Me/H-7″, H-7″/H-2″, and H-7″/H-6″, OH-7″/H-6″, and OH-7″/N-Me, supported the assignment of the configurations at C-7′′ as S*. Based on these observations, the relative configuration of **1** was established.

**Fig. 4:**
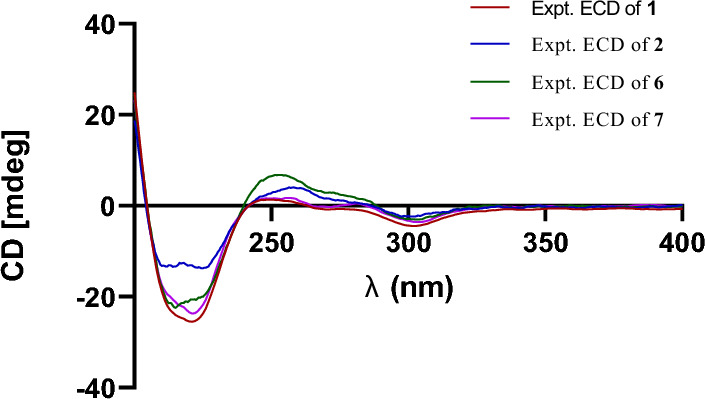
Experimental ECD spectra of compounds **1**, **2**, **6**, and **7**

**Fig. 5:**
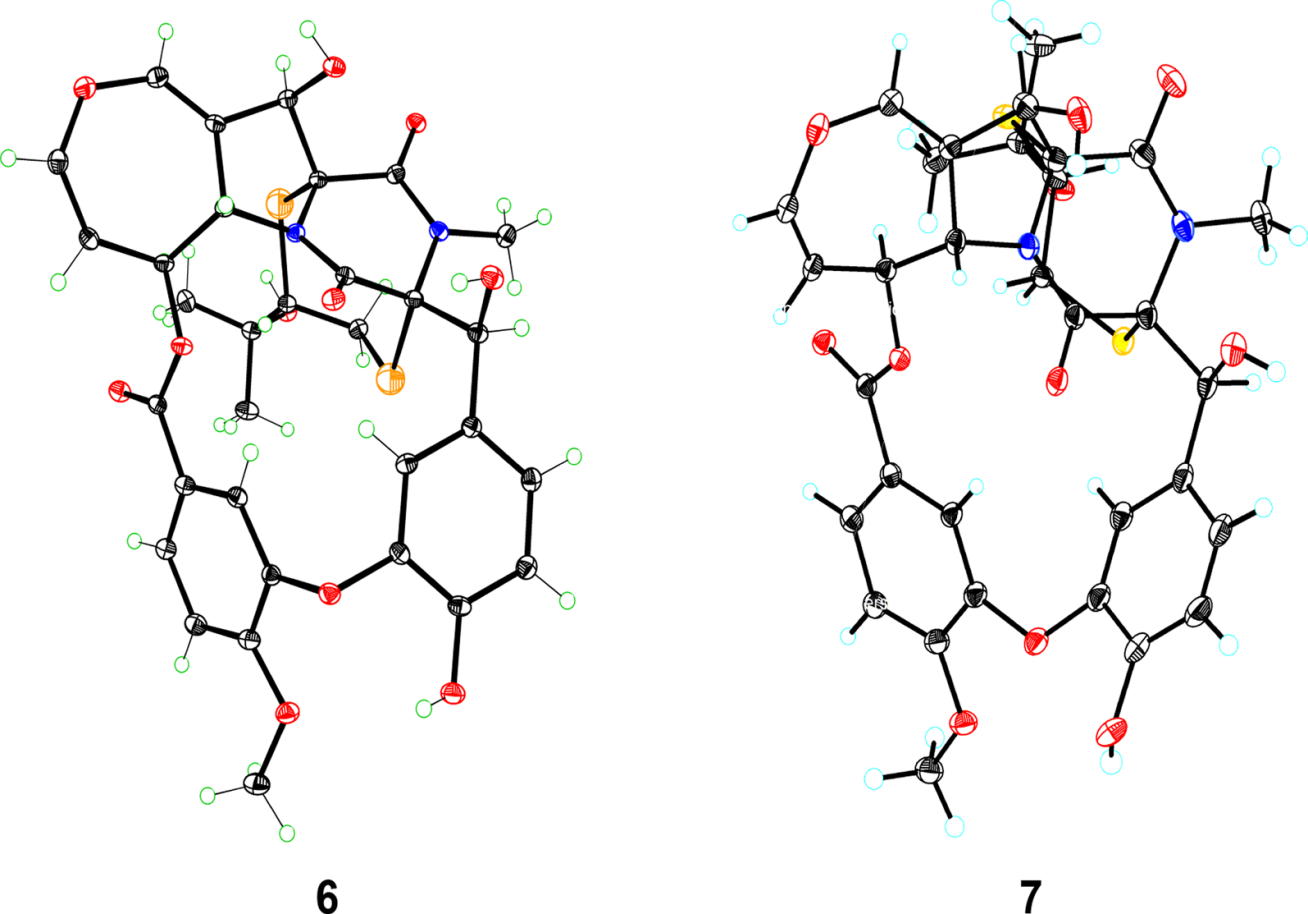
ORTEP drawing of the X-ray crystal structures of **6** and **7**

Prenylemestrin D (**2**) was determined to share the same molecular formula, C_32_H_32_N_2_O_11_S_2_, as compound **1**, based on HRESIMS and NMR spectra. A comparison of the 1D (Tables 1 and 2) and 2D NMR spectra (Fig. 3) revealed significant similarities between the two compounds, except for the slightly different chemical shifts of C-1 (**2**: *δ*_C_ 170.4; **1**: *δ*_C_ 167.1), C-4 (**2**: *δ*_C_ 165.4; **1**: *δ*_C_ 161.5), and C-11a (**2**: *δ*_C_ 75.3; **1**: *δ*_C_ 78.0), which inferred that **2** could be the C-13 epimer of **1** (Fig. 1). This deduction was further supported by NOESY interactions of H-13/N-Me and H-6/H-12a (*δ*_H_ 2.76) in **2**. Additionally, the experimental ECD spectra (Fig. 4) of compounds **1** and **2** closely matched those of **6** and **7**, whose absolute configurations had been previously confirmed by X-ray diffraction (Fig. 5). These findings conclusively established the absolute configurations of **1** and **2** as depicted in Fig. 1.

**Fig. 6:**
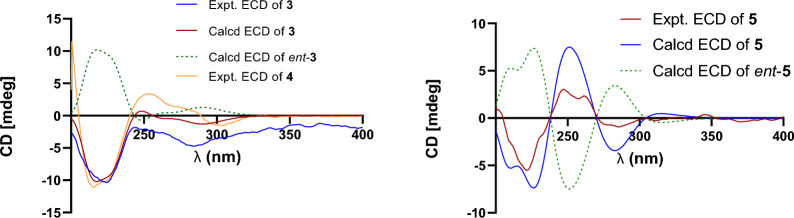
Calculated and experimental ECD spectra of **3** and **5**

Prenylemestrin E (**3**) was assigned the molecular formula of C_32_H_32_N_2_O_11_S_2_, as indicated by its HRESIMS spectrum, showing an ion peak at *m/z* 707.1372 ([M + Na]^+^, calcd. for C_32_H_32_N_2_O_11_S_2_Na^+^, 707.1345), corresponding 18 degrees of unsaturation. The 1D NMR data of **3** closely resembled those of **1** and **2** (Tables 1 and 2), except for the appearance of distinctive signals for a conjugated aldehyde group (*δ*_C_ 190.9 and *δ*_H_ 9.75), and a methine (C-3, *δ*_H_ 4.96, s; *δ*_C_ 65.7), which replaced the nonprotonated carbon C-3 (*δ*_C_ 76.5) in **1**. These differences suggested that **3** was structurally related to secoemestrin C, with cleavage of the 15-membered macrolactone ring between C-3 and C-7′′. HMBC correlations from H-7′′ to C-1′′, C-2′′, and C-6′′ confirmed the presence and position of an aldehyde group. Moreover, HMBC interactions from H-13 to C-3, and from H_2_-12 to C-11a along with the ^1^H − ^1^H COSY correlations of H_2_-12/H-13 further established the caged core structure of **3** as consistent with that of **1** and **2**. The relative configuration of **3** was deduced from the NOESY spectrum and coupling constant analysis. The coupling constants (*J*_H‑5a/H‑6_ = 8.0 Hz) indicated that H-5a was *trans*-oriented. The NOESY correlation of between H-11 and H-6 indicated that H-11 is *α*-oriented. The observed NOESY correlation (Fig. 3) of H-6/H-13 suggested that the configuration of C-13 was R*, with the C-3−S and C-11a−S bonds being *α*-oriented, and H-3 being *β*-oriented. Furthermore, the theoretical ^13^C NMR calculations with DP4 + analysis of two isomers13R*-**3** and 13S*-**3** were conducted through the GIAO method at the mPW1PW91/6-311G(d,p) level in chloroform with the Gaussian 09 software. The results (Fig. S1) showed that 13R*-**3** had a better coefficient of determination (*R*^2^ = 0.9983) between the experimental and calculated ^13^C NMR chemical shits than 13S*-**3** (*R*^2^ = 0.9964). Additionally, the DP4 + analysis with a high probability of 100% permitted the relative configuration 13R*-**3**. To further confirm the absolute configuration of **3**, the ECD spectra of (3R*,5aS*,6S*,11R*,11aR*,13R*)-**3** were calculated using time-dependent density functional theory (TDDFT) methods (Tables S3 and S4). The calculated spectra matched well with the experimental data (Fig. 6), leading to the assignment of the absolute configurations of **3** as 3*R*,5a*S*,6*S*,11*R*,11a*R*,13*R*.

Prenylemestrin F (**4**) was determined to have the same molecular formula, C_32_H_32_N_2_O_11_S_2_, as compound **3**, as confirmed by HRESIMS and NMR data. The ^1^H and ^13^C NMR data (Tables 1 and 2) of **4** closely resembled those of **3**, indicating a similar structural framework. Further interpretation of the ^1^H − ^1^H COSY and HMBC spectra (Fig. 2) confirmed that compound **4** shares the same ETPs core as **3**. Notable differences between **3** and **4** were observed in the chemical shifts of C-1 and C-4, which were significantly downfield-shifted by + 4.5 ppm and + 4.9 ppm, respectively, in **3**. Detailed ^1^H − ^1^H COSY and HMBC analyses confirmed that both compounds possess the same planar structure. Therefore, **3** and **4** were concluded to be stereoisomers. The NOESY interaction of H-13/N-Me was observed for **4**, as opposed to H-13/H-6 in **3**, which, along with careful NOESY elucidation, suggested that **4** is the C-13 epimer of **3**. Furthermore, the experimental ECD spectra (Fig. 6) of compound **4** closely matched those of **3**, leading to the determination of the absolute configuration of **4** as 3*R*,5a*S*,6*S*,11*R*,11a*R*,13*S*.

Prenylemestrin G (**5**) was assigned the same molecular formula of C_32_H_32_N_2_O_11_S_2_ as **3** and **4**, based on HRESIMS analysis. The ^1^H and ^13^C NMR data of **5** (Tables 1 and 2) closely resembled those of **3**. HMBC correlations (Fig. 2) from H_2_-12 to C-3, H-13 to C-11a confirmed that the connection between the hemiterpene moiety and the ETP core in **5** was consistent with prenylemestrins A and B (**6** and **7**) [17]. NOESY correlations of H-6/H-13 (Fig. 3) indicated that H-13 in **5** adopts an *α*-orientation. The absolute configuration of **5** was determined by comparison its experimental ECD spectrum with the calculated one. The calculated ECD spectrum for (3*R*,5a*S*,6*S*,11*R*,11a*R*,13*S*)-**5** was in good agreement with the experimental data (Fig. 6), leading to the assignment of the absolute configuration as **5** as 3*R*,5a*S*,6*S*,11*R*,11a*R*,13*S*.

Based on previous biosynthetic studies of ETPs, hypothetic biogenetic pathways of **1**−**7** were proposed to explain their origins (Scheme S1) with two molecules of L-phenylalanine as precursors. The key intermediate **I** was obtained *via* a series of peptide cyclization, ring-expansion, and esterification reactions. Then, intermediate **I** underwent methylation and macrocyclization to form **II**. Sulfurization of the intermediate **II** at C-3 and C-11a produced the key dithiol intermediate **III**, which was further decorated with dimethylallyl diphosphate via pathway a or *b* to form **III** or **V**, respectively. Subsequently, the followed epoxidation and nucleophilic attack of the sulfhydryl group led to the production of **1**−**2** and** 6**−**7**. The intermediate **VI**, with the C-3−C-7′′ bond cleavaged, could be derived from **III**; ultimately, compounds **3**−**5** could be derived *via* pathways *c* and *d*, with dimethylallyl diphosphate and modified farnesyl diphosphate, respectively.

**Fig. 7:**
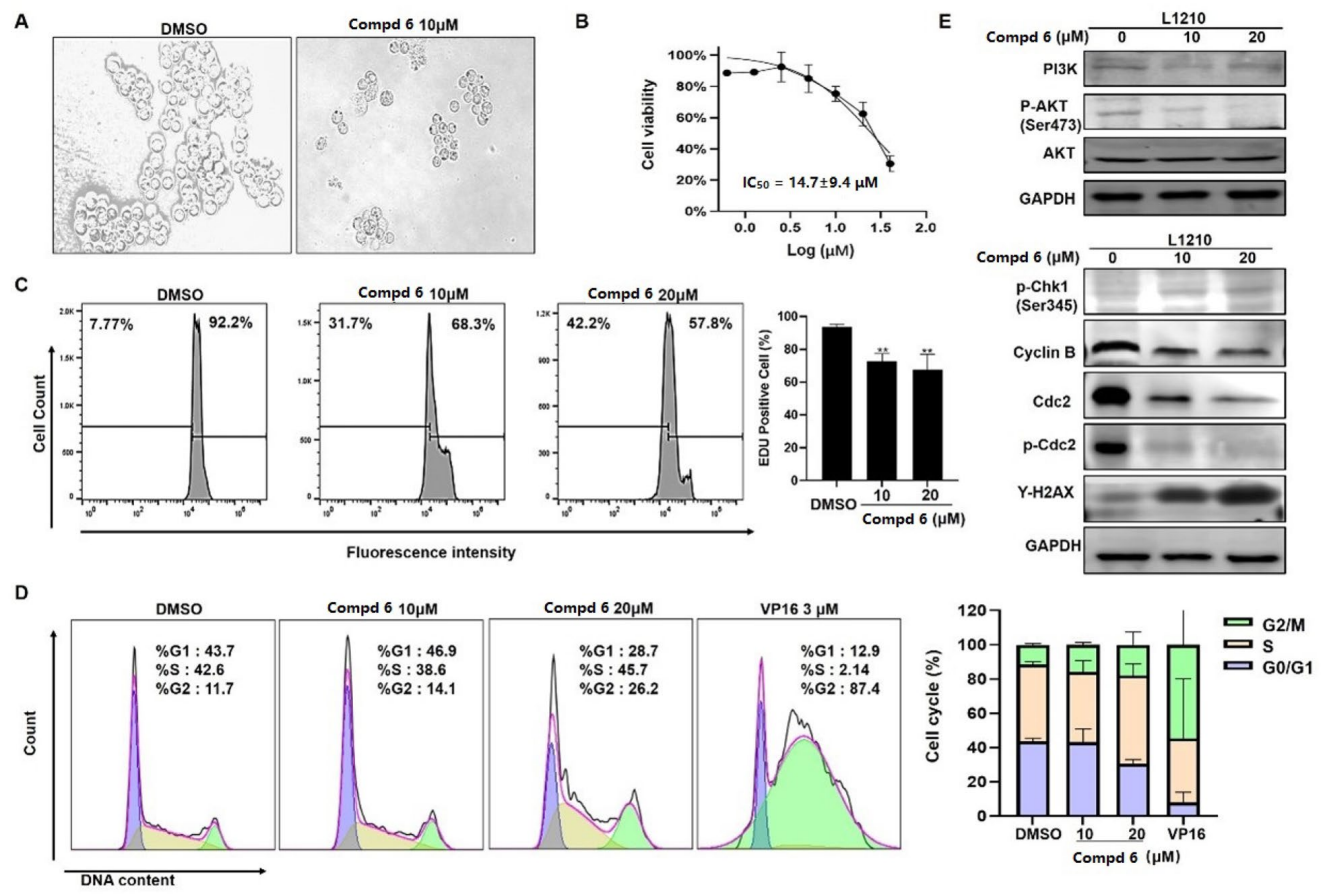
Compound **6** inhibited proliferation and induced G2/M cell cycle arrest in L1210 cells. Morphologic change of L1210 cells after treatment with **6** (**A**); Cell proliferation curve of CCK-8 viability assay (**B**); EdU assay showed that EdU positive ratio was affected by **6** treated (**C**). Cell cycle distribution (**D**); Western blot analysis of PI3K/AKT and cell cycle related proteins, with GAPDH was used as loading control (**E**)

**Fig. 8:**
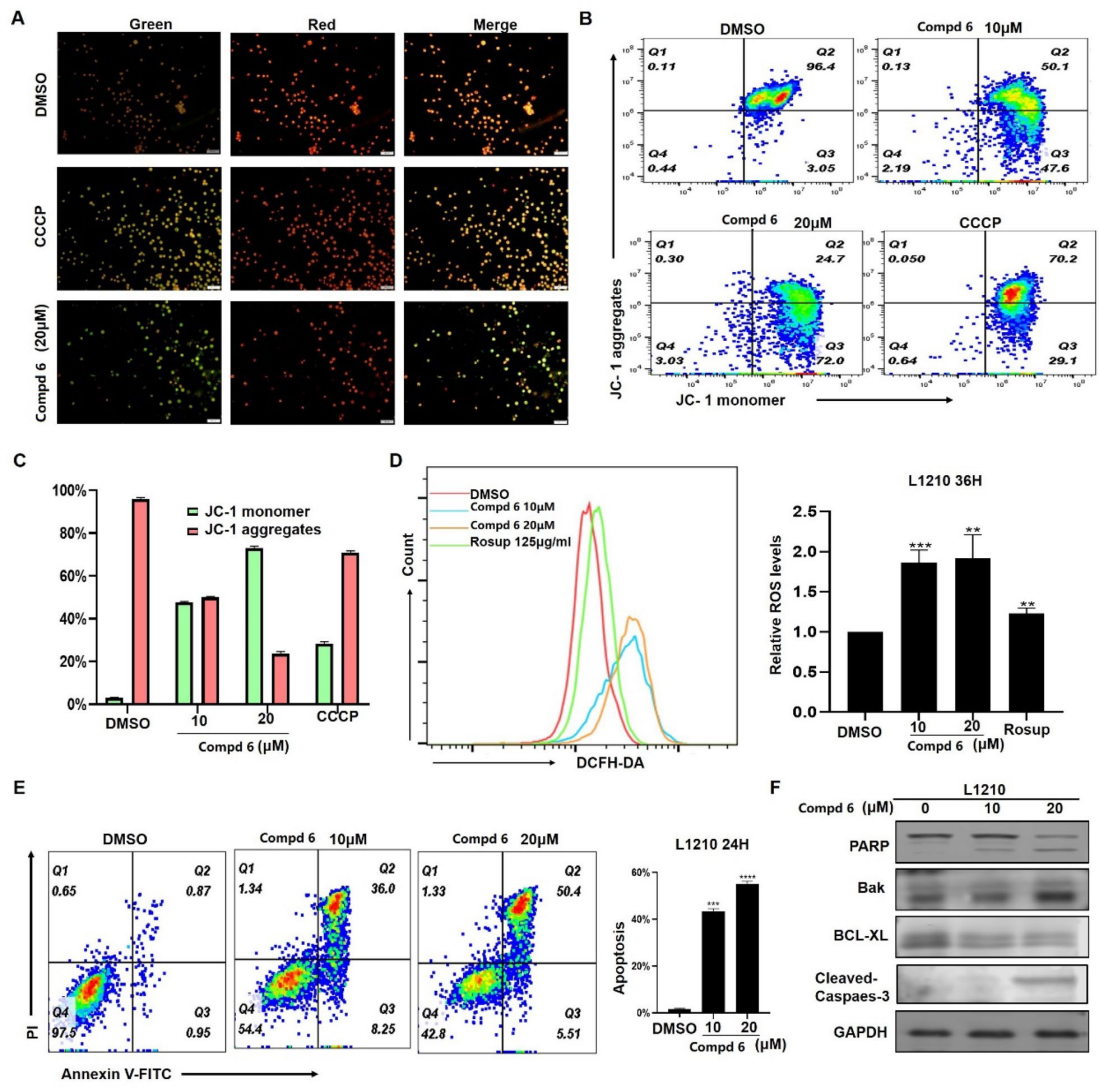
Effect of compound **6** on L1210 cells. Treat L1210 cells with different concentrations of **6** for 24 h and detect mitochondrial membrane potential status using JC-1 staining kit (**A**−**C**). **D** Fluorescence data of DCFH-DA staining of cells treated with **6** or Rosup for 36 h. **E** Cell apoptosis was determined by flow cytometric analysis using Annexin V-FITC and PI staining. **F** Western blot analysis of apoptosis related proteins, with GAPDH used as loading control

In our biological evaluation, compounds **1**−**7** exhibited no anti-inflammatory activity. Additionally, compounds **1**−**5** and **7** showed no cytotoxicity against the tested cell lines (A549, L1210, HL-60, SW-480, and Hep3B). Interestingly, **6** demonstrated moderate cytotoxicity against L1210 mouse leukemia cells. Using L1210 cells as a model, we further investigated the antitumor effect and the underlying mechanism of compound **6**. The results revealed that **6** inhibited proliferation and induced G2/M cell cycle arrest in L1210 cells by regulating PI3K/AKT pathway and cell cycle-related proteins, such as p-Chk1, Cyclin B, Cdc2, p-Cdc2, and γh2ax (Fig. 7). Furthermore, **6** inhibited mitochondrial membrane potential (MMP), increased ROS levels, and induced apoptosis in L1210 cells (Fig. 8). These changes in MMP and ROS levels suggest that the antitumor effects of **6** are closely related to mitochondrial dysfunction. Western blot analysis further supported this hypothesis, showing that **6** altered the expression of mitochondria-related proteins, including Bak and Bcl-xl, indicating that apoptosis induced by **6** occurs predominantly *via* the mitochondrial pathway. The antitumor effects of compound **6** were also validated in other leukemia cell lines, with results consistent with those observed in L1210 cells (data not shown). These findings highlight the potential of **6** as a candidate for anti-leukemia research, warranting further in-depth studies on its mechanism and therapeutic potential.

## Conclusion

This study elucidates the structures of prenylemestrins C−G (**1**−**5**) and highlights the moderate cytotoxicity of compound **6**, which is likely attributed to its distinct structural features. Compound **6** was found to induce G2/M cell cycle arrest and apoptosis in L1210 cells, primarily through the regulation of the signaling pathway and mitochondrial apoptotic mechanisms. These findings underscore its significant impact on cell growth and survival. The discovery of prenylemestrins C−G expands the structural diversity of epipolythiodioxopiperazine (ETP) compounds and provides a solid foundation for investigating their pharmacological and medicinal potential. Future research should focus on a detailed exploration of the mechanism of action of compound **6** and its potential therapeutic applications in cancer treatment. Additionally, the biological activities and pharmacological properties of other prenylemestrins, as well as their interactions with cellular signaling pathways, warrant further study.

In conclusion, this work not only uncovers the structures and bioactivities of novel ETP compounds but also paves the way for their exploration in synthetic and pharmacological research, fostering interest in their potential applications in drug discovery and development.

## Experimental

### General experimental procedures

Using Shanghai SGW® X-4B microscopic melting point instrument (uncorrected, China) and hot stage melting point determination to measure melting points. HRESIMS was performed on a microOTOF-Q-II acquisition parameter (Bruker, Germany). Measure the optical rotations of compounds in methanol or dichloromethane using a Perkin Elmer 341 polarimeter. The ultraviolet (UV) spectrum was obtained with the Lambda 35 UV-1 spectrometer (PerkinElmer, US). CD curve was obtained on the J-810 spectrometer (JASCO, Japan). IR spectrum was acquired on a Nicolet iS50R FT-IR spectrometer (Thermo Scientific, US). NMR spectrum was recorded on an AM-400 and an AVANCE NEO 600 spectrometer (Bruker, Germany), with chemical shifts are expressed in ppm relative to CD_3_OD (*δ*_C_ 49.0, *δ*_H_ 3.31) and CDCl_3_ (*δ*_C_ 77.16, *δ*_H_ 7.26). Silica gel (100–200 mesh and 200–300 mesh, Qingdao Marine Chemical Inc., Qingdao, China) was used for normal-phase column chromatography (CC). Reversed-phase CC was accomplished using YMC-Gel (50 μm, YMC, Japan), and Sephadex LH-20 (Pharmacia Biotech AB, Uppsala, Sweden) were used for CC performed with methanol/dichloromethane as the mobile phase. Thin-layer chromatography (TLC) analyses were carried out with precoated silica gel F_254_ plates (Qingdao Marine Chemical Inc., Qingdao, China). The HPLC separations were conducted on an Agilent 1220 HPLC system equipped with a UV detector, accompanied by reversed-phase Ultimate XB-C_18_ column (5 μm, 10 × 250 mm). 10% H_2_SO_4_ in EtOH was used to visualize spots in TLC.

### Fungal material

The fungus *Aspergillus nidulans*, deposited at the Huazhong University of Science and Techbology (HUST, 414JZ-1), Hubei Province, China, was isolated from the Annelida *Whitmania pigra* Whitman. The ITS sequence data for this strain have been submitted to DDBJ/EMBL/GenBank under accession No. MK397763.

### Fermentation, extraction, and isolation

The fungus *A. nidulans* was maintained as seeds on potato dextrose agar (PDA) medium at 28 °C for 6 days, which was carried out on rice medium at room temperature for three weeks. After incubation, extract the fermented rice substrate seven times with ethanol. After removing the solvent under vacuum, the entire extract was suspended in water and then extracted with ethyl acetate to obtain a dark solid (400.0 g). Based on the TLC analysis, dark solid was chromatographed over silica gel using petroleum ether/ethyl acetate (20:1, 10:1, 5:1, 3:1, 1:1, 0:1, v/v) and ethyl acetate/methanol gradient (60:1 to methanol, v/v) as eluting solvent, to yield six subfractions (Fr.1−Fr.6). Fr.3 (1.5 g) was further separated through Sephadex LH-20 by eluting with dichloromethane/methanol (1:1, v/v), to produce three major fractions (Fr.3.1–Fr.3.3), Fr.3.3 (210 mg) was further separated through a silica gel column eluted with dichloromethane/methanol (100:1, 80:1, 50:1, v/v) and semipreparative HPLC to yield **4** (acetonitrile/water, 65:35, 2.0 mL/min; *t*_R_ 14.0 min, 2 mg). The Fr.4 (400 mg) was fractionated on a silica gel column eluted with dichloromethane/methanol (200:1, 100:1, 80:1, 50:1, v/v) to yield six major fractions (Fr.4.1–Fr.4.6), Fr.4.2 (100 mg) was further separated via a Sephadex LH-20 CC in methanol and purified by HPLC to obtain compound **6** (acetonitrile/water, 46:54, 2.0 mL/min; 27 mg; *t*_R_ 39.3 min), Fr.4.3 (89.2 mg) was separated by a Sephadex LH-20 CC in dichloromethane/methanol (1:1, v/v) and further purified by HPLC to yield compounds **2** (acetonitrile/water, 40:60, 2.0 mL/min; 10.2 mg; *t*_R_ 35.2 min) and **5** (acetonitrile/water, 42:58, 2.0 mL/min; 11.5 mg; *t*_R_ 13.0 min). The Fr.5 (12 g) was subjected to an reverse phase C_18_ silica gel eluted with a gradient methanol/water (20%, 40%, 60%, 80%, 100%, v/v) to yield eight fractions (Fr.5.1−Fr.5.8), Fr.5.3 (260 mg) was separated through Sephadex LH-20 eluted with dichloromethane/methanol (1:1, v/v), and followed by semipreparative HPLC to yield compounds **1** (acetonitrile/water, 40:60, 2.0 mL/min; *t*_R_ 36.0 min, 20 mg) and **3** (acetonitrile/water, 40:60, 2.0 mL/min; *t*_R_ 48.5 min, 1.9 mg). Fr.5.6 (2.3 g) was chromatographed on a silica gel column by eluting with dichloromethane/methanol (100:1, 80:1, 60:1, v/v) to afford three fractions Fr.5.6.1 − Fr.5.6.3. Fr.5.6.2 (146.0 mg) was further purified to semipreparative HPLC to yield **7** (acetonitrile/water, 30:70, 2.0 mL/min; *t*_R_ 36.0 min, 38.8 mg).

*Prenylemestrin C (****1****):* White powder, ${[\alpha ]}_{\text{D}}^{20}$ –99 (*c* 0.1, MeOH); UV (MeOH) λ_max_ (log *ε*) = 203 (4.90), 263 (4.33) nm; IR (KBr) *ν*_max_ = 3419, 2942, 2838, 1691, 1662, 1513, and 1381 cm^−1^; ECD (MeOH) λ_max_ (Δ*ε*) = 221 (–50.7), 275 (–1.4), 302 (–8.8) nm; ^1^H and ^13^C NMR data see Tables S1 and S2; HRMS (ESI-TOF) *m/z*: [M + Na]^+^ 707.1345 (calcd for C_32_H_32_N_2_O_11_S_2_Na, 707.1345).

*Prenylemestrin D (****2****):* White powder; ${[\alpha ]}_{\text{D}}^{20}$ –29 (*c* 0.1, MeOH); UV (MeOH) λ_max_ (log *ε*) = 262 (4.08), 202 (4.62) nm; IR (KBr) *ν*_max_ = 3423, 2974, 2954, 2934, 1675, 1653, 1513, and 1383 cm^−1^; ECD (MeOH) λ_max_ (Δ*ε*) = 225 (–34.3), 258 (+ 10.0), 303 (–5.9) nm; ^1^H and ^13^C NMR data see Tables S1 and S2; HRMS (ESI-TOF) *m/z*: [M + Na]^+^ 707.1377 (calcd for C_32_H_32_N_2_O_11_S_2_Na, 707.1345).

*Prenylemestrin E (****3****):* White powder; ${[\alpha ]}_{\text{D}}^{20}$ –15 (*c* 0.1, MeOH); UV (MeOH) λ_max_ (log *ε*) = 264 (4.33), 228 (4.50), 203 (4.68) nm; IR (KBr) *ν*_max_ = 3430, 2920, 2850, 1681, 1606, 1512, and 1384 cm^−1^; ECD (MeOH) λ_max_ (Δ*ε*) = 224 (–25.8), 284 (–11.8) nm; ^1^H and ^13^C NMR data see Tables S1 and S2; HRMS (ESI-TOF) *m/z*: [M + Na]^+^ 707. 1372 (calcd for C_32_H_32_N_2_O_11_S_2_Na, 707.1345).

*Prenylemestrin F (****4****):* White powder; ${[\alpha ]}_{\text{D}}^{20}$ –67 (*c* 0.1, MeOH); UV (MeOH) λ_max_ (log *ε*) = 263 (4.38), 226 (4.56) nm; IR (KBr) *ν*_max_ = 3429, 2922, 2850, 1681, 1605, 1511, and 1277 cm^−1^; ECD (MeOH) λ_max_ (Δ*ε*) = 215 (–55.8), 253 (+ 16.8), 304 (–7.6) nm; ^1^H and ^13^C NMR data see Tables S1 and S2; HRMS (ESI-TOF) *m/z*: [M + Na]^+^ 707.1433 (calcd for C_32_H_32_N_2_O_11_S_2_Na, 707.1345).

*Prenylemestrin G (****5****):* White powder; ${[\alpha ]}_{\text{D}}^{20}$ +51 (*c* 0.1, MeOH); UV (MeOH) λ_max_ (log *ε*) = 263 (4.43), 204 (4.92) nm; IR (KBr) *ν*_max_ = 3363, 2920, 2850, 1683, 1662, 1604, 1511, and 1277 cm^−1^; ECD (MeOH) λ_max_ (Δ*ε*) = 221 (–59.0), 256 (+ 4.27), 274 (–0.2), 281 (+ 1.2) nm; ^1^H and ^13^C NMR data see Tables S1 and S2; HRMS (ESI-TOF) *m/z*: [M + Na]^+^ 707.1338 (calcd for C_32_H_32_N_2_O_11_S_2_Na, 707.1345).

### X-ray crystal structure analysis

After crystallization from methanol using the vapor diffusion method, colorless crystals of **6** and **7** were obtained at room temperature. Collect crystal data using Bruker D8 Quest (Bruker, Germany) with a graphite-monochromated Cu K*α* radiation. The structure was solved with the SHELXTL refinement packages using least squares minimization. All crystallographic data were stored in the Cambridge Crystallographic Data Centre (2133579 for **6** and 2133560 for **7**).

Crystal data of **6**. C_32_H_32_N_2_O_11_S_2_, *M* = 684.72, space group *P*212121, *a* = 9.416(0) Å, *b* = 10.339(0), *c* = 33.754(0), *α* = *β* = *γ* = 90°, *V* = 3286.0(3) Å^3^, *T* = 293(2) K, *Z* = 4, *μ*(Cu K*α*) = 2.012 mm^−1^, 83,642 reflections measured, 6631 independent reflections (*R*_*int*_ = 0.0325). The final *R*_*1*_ values = 0.0332 (*I* > 2*σ*(*I*)), *wR*(*F*^2^) values = 0.1116 (*I* > 2*σ*(*I*)), *R*_*1*_ values = 0.0334 (all data), and *wR*(*F*^2^) values = 0.1118 (all data). The goodness of fit on *F*^2^ = 1.044. Flack parameter = 0.023(4). m.p. 181** − **184 °C.

Crystal data of** 7**. C_32_H_32_N_2_O_11_S_2_•4(H_2_O), *M* = 748.71, space group *P*43212, *a* = 18.8206(4) Å, *b* = 18.8206(4) Å, *c* = 20.6776(4) Å, *α* = *β* = *γ* = 90°, *V* = 7324.3(3) Å^3^, *T* = 100(2) K, *Z* = 8, *μ*(Cu K*α*) = 1.938 mm^−1^, 108,024 reflections measured, 7232 independent reflections (*R*_*int*_ = 0.0743). The final *R*_*1*_ values = 0.0807 (*I* > 2*σ*(*I*)), *wR*(*F*^2^) values = 0.2240 (*I* > 2*σ*(*I*)), *R*_*1*_ values = 0.1109 (all data), and *wR*(*F*^2^) values = 0.2508 (all data). The goodness of fit on *F*^2^ = 1.562. Flack parameter = 0.021(7). m.p. 188 − 190 °C.

### Computational section for electronic circular dichroism (ECD)

The methodology utilized for the theoretical computation of ECD spectra, as well as the production of corresponding calculated ECD spectra, remained consistent with the previously outlined procedure[19]. The details were described in the supplementary material.

## Supplementary Information

Supplementary Material 1. Supplementary data associated with this article (biological assay; ECD calculation result of compounds 3 and 5; 1D and 2D-NMR, HRESIMS, UV, IR spectra for compounds **1** − **5**; 1D NMR spectra for compounds **6** and **7**).

## Data Availability

The datasets used or analysed during the current study are available from the corresponding author on reasonable request.
