# Supplementary material for: Emestrin-type epipolythiodioxopiperazines from Aspergillus nidulans with cytotoxic activities by regulating PI3K/AKT and mitochondrial apoptotic pathways
Source: Nat Prod Bioprospect. 2025 Mar 10;15(1):17. doi: 10.1007/s13659-025-00498-8 (PMC11891114; doi:10.1007/s13659-025-00498-8)
Supplement: Supplementary file 1 — Supplementary Material 1. Supplementary data associated with this article (biological assay; ECD calculation result of compounds 3 and 5; 1D and 2D-NMR, HRESIMS, UV, IR spectra for compounds 1 − 5; 1D NMR spectra for compounds 6 and 7). [file 13659_2025_498_MOESM1_ESM.pdf]

# Supporting Information

## Emestrin-Type Epipolythiodioxopiperazines from *Aspergillus nidulans* with Cytotoxic Activities by Regulating PI3K/AKT and Mitochondrial Apoptotic Pathways

Pengkun Li[1Δ], Qin Li[1Δ], Aimin Fu[1], Yang Xiao[1], Chunmei Chen[1], Hucheng Zhu[1], Changxing Qi[1], Wei Wei[2*], Yuan Zhou[1*], and Yonghui Zhang[1*]

[1]*Hubei Key Laboratory of Natural Medicinal Chemistry and Resource Evaluation, School of Pharmacy, Tongji Medical College, Huazhong University of Science and Technology, Wuhan 430030, People's Republic of China*

[2]*China National Center for Biotechnology Development, Beijing, 100039, China, People's Republic of China*

**[*]Corresponding authors.**

*E-mail addresses*: zhangyh@mails.tjmu.edu.cn (Y. Zhang), zhouyuan@hust.edu.cn (Y. Zhou), weiwei@cncbd.org.cn (W.Wei)

[Δ]These authors contributed equally.

# List of Supporting Information

**Biological assays** ........................................ 5

**ECD calculation Results** ........................................ 9

**Figure S1.** NMR calculations of **3** with DP4+ probability analysis between 13*R**-**3** and 13*S**-**3**. ........................................ 4
**Figure S2.** DP4+ probability analysis of $^{13}$C NMR chemical shifts for isomers 13*R**-**3** and 13*S**-**3**. ........................................ 4
**Figure S3.** $^{1}$H NMR (400 MHz) spectrum of **1** recorded in $CD_3OD$ ........................................ 39
**Figure S4**. $^{13}$C NMR (100 MHz) spectrum of **1** recorded in $CD_3OD$ ........................................ 39
**Figure S5.** DEPT NMR spectrum of **1** recorded in $CD_3OD$ ........................................ 40
**Figure S6.** HSQC spectrum of **1** recorded in $CD_3OD$ ........................................ 40
**Figure S7.** HMBC spectrum of **1** recorded in $CD_3OD$ ........................................ 41
**Figure S8.** $^{1}$H–$^{1}$H COSY spectrum of **1** recorded in $CD_3OD$ ........................................ 41
**Figure S9.** NOESY spectrum of **1** recorded in $CD_3OD$ ........................................ 42
**Figure S10.** HRESIMS spectrum of **1** ........................................ 42
**Figure S11.** IR spectrum of **1** ........................................ 43
**Figure S12.** UV spectrum of **1** ........................................ 43
**Figure S13.** $^{1}$H NMR (600 MHz) spectrum of **2** recorded in $CD_3OD$ ........................................ 44
**Figure S14.** $^{13}$C NMR (150 MHz) spectrum of **2** recorded in $CD_3OD$ ........................................ 44
**Figure S15.** DEPT NMR spectrum of **2** recorded in $CD_3OD$ ........................................ 45
**Figure S16.** HSQC spectrum of **2** recorded in $CD_3OD$ ........................................ 45
**Figure S17.** HMBC spectrum of **2** recorded in $CD_3OD$ ........................................ 46
**Figure S18.** $^{1}$H–$^{1}$H COSY spectrum of **2** recorded in $CD_3OD$ ........................................ 46
**Figure S 19.** NOESY spectrum of **2** recorded in $CD_3OD$ ........................................ 47
**Figure S20.** HRESIMS spectrum of **2** ........................................ 47
**Figure S21.** IR spectrum of **2** ........................................ 48
**Figure S22.** UV spectrum of **2** ........................................ 48
**Figure S23.** $^{1}$H NMR (600 MHz) spectrum of **3** recorded in $CDCl_3$ ........................................ 49
**Figure S24.** $^{13}$C NMR (150 MHz) spectrum of **3** recorded in $CDCl_3$ ........................................ 49
**Figure S25.** DEPT NMR spectrum of **3** recorded in $CDCl_3$ ........................................ 50
**Figure S26.** HSQC spectrum of **3** recorded in $CDCl_3$ ........................................ 50
**Figure S27.** HMBC spectrum of **3** recorded in $CDCl_3$ ........................................ 51
**Figure S28.** $^{1}$H–$^{1}$H COSY spectrum of **3** recorded in $CDCl_3$ ........................................ 51
**Figure S29.** NOESY spectrum of **3** recorded in $CDCl_3$ ........................................ 52
**Figure S30.** HRESIMS spectrum of **3** ........................................ 52
**Figure S31.** IR spectrum of **3** ........................................ 53
**Figure S32.** UV spectrum of **3** ........................................ 53
**Figure S33.** $^{1}$H NMR (600 MHz) spectrum of **4** recorded in $CD_3OD$ ........................................ 54
**Figure S34.** $^{13}$C{$^{1}$H} NMR (150 MHz) spectrum of **4** recorded in $CD_3OD$ ........................................ 54
**Figure S35.** DEPT NMR spectrum of **4** recorded in $CD_3OD$ ........................................ 55
**Figure S36.** HSQC spectrum of **4** recorded in $CD_3OD$ ........................................ 55
**Figure S37.** HMBC spectrum of **4** recorded in $CD_3OD$ ........................................ 56
**Figure S38.** $^{1}$H–$^{1}$H COSY spectrum of **4** recorded in $CD_3OD$ ........................................ 56

**Figure S39.** NOESY spectrum of **4** recorded in $CD_3OD$ ........ 57
**Figure S40.** HRESIMS spectrum of **4** ........ 57
**Figure S41.** IR spectrum of **4** ........ 58
**Figure S42.** UV spectrum of **4** ........ 58
**Figure S43.** $^1H$ NMR (600 MHz) spectrum of **5** recorded in $CDCl_3$ ........ 59
**Figure S44.** $^{13}C$ NMR (150 MHz) spectrum of **5** recorded in $CDCl_3$ ........ 59
**Figure S45.** DEPT NMR spectrum of **5** recorded in $CDCl_3$ ........ 60
**Figure S46.** HSQC spectrum of **5** recorded in $CDCl_3$ ........ 60
**Figure S47.** HMBC spectrum of **5** recorded in $CDCl_3$ ........ 61
**Figure S48.** $^1H$–$^1H$ COSY spectrum of **5** recorded in $CDCl_3$ ........ 61
**Figure S49.** NOESY spectrum of **5** recorded in $CDCl_3$ ........ 62
**Figure S50.** HRESIMS spectrum of **5** ........ 62
**Figure S51.** IR spectrum of **5** ........ 63
**Figure S52.** UV spectrum of **5** ........ 63
**Figure S53.** $^1H$ NMR (400 MHz) spectrum of **6** recorded in $CD_3OD$ ........ 64
**Figure S54.** $^{13}C$ NMR (100 MHz) spectrum of **6** recorded in $CD_3OD$ ........ 64
**Figure S55.** DEPT NMR spectrum of **6** recorded in $CD_3OD$ ........ 65
**Figure S56.** $^1H$ NMR (400 MHz) spectrum of **7** recorded in $CD_3OD$ ........ 65
**Figure S57.** $^{13}C$ NMR (100 MHz) spectrum of **7** recorded in $CD_3OD$ ........ 66
**Figure S58.** DEPT NMR spectrum of **7** recorded in $CD_3OD$ ........ 66

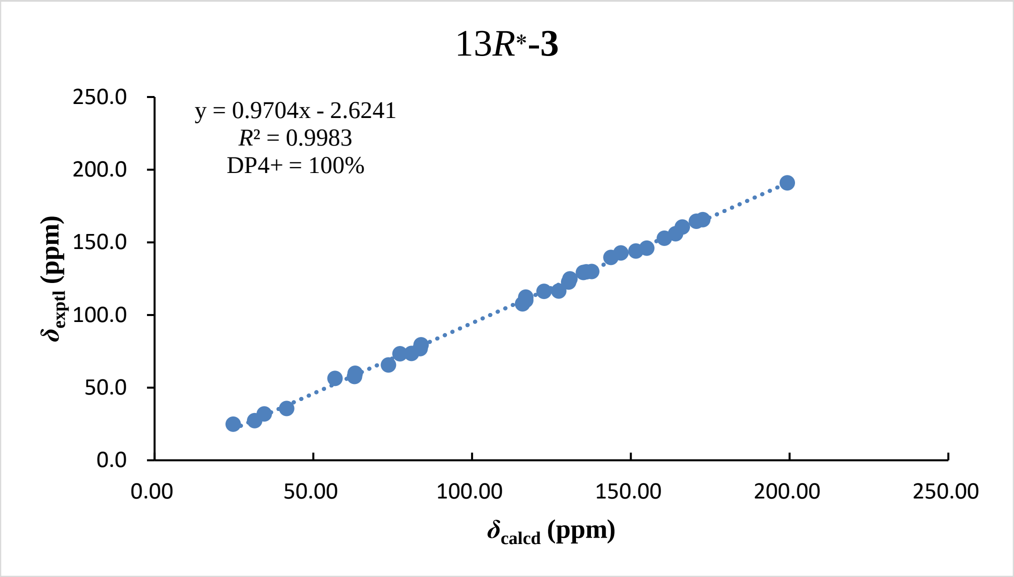


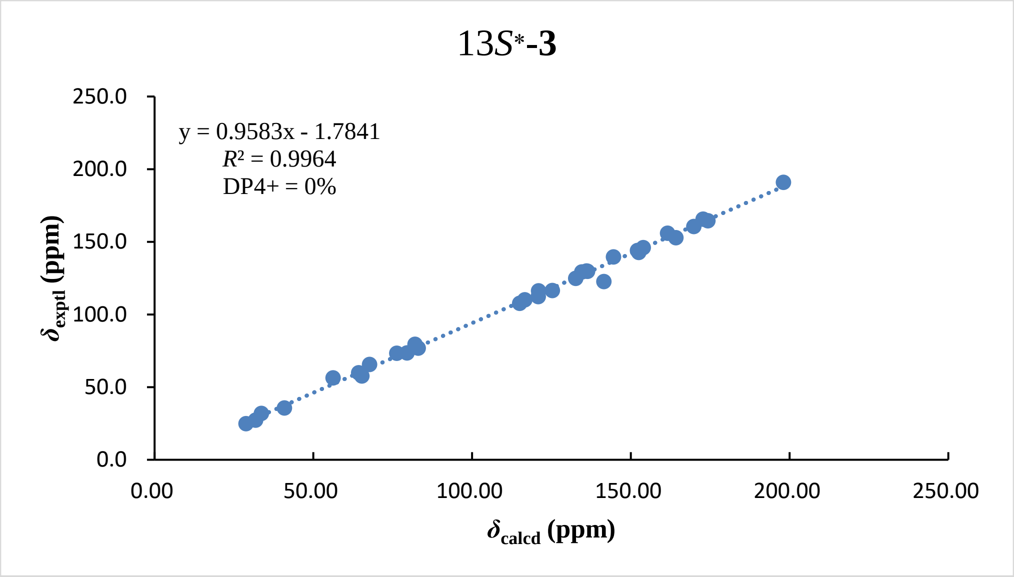


**Figure S1.** NMR calculations of **3** with DP4+ probability analysis between 13*R**-**3** and 13*S**-**3**.

| **Isomer N°** | | 13*R**-**3** | 13*S**-**3** |
|---|---|---|---|
| | **H data** | - | - |
| **DP4+ (%)** | **C data** | 100.00% | 0.00% |
| | **All data** | **100.00%** | **0.00%** |

**Figure S2.** DP4+ probability analysis of $^{13}C$ NMR chemical shifts for isomers 13*R**-**3** and 13*S**-**3**.

**Scheme S1.** Hypothetical biosynthesis of **1**−**7**.

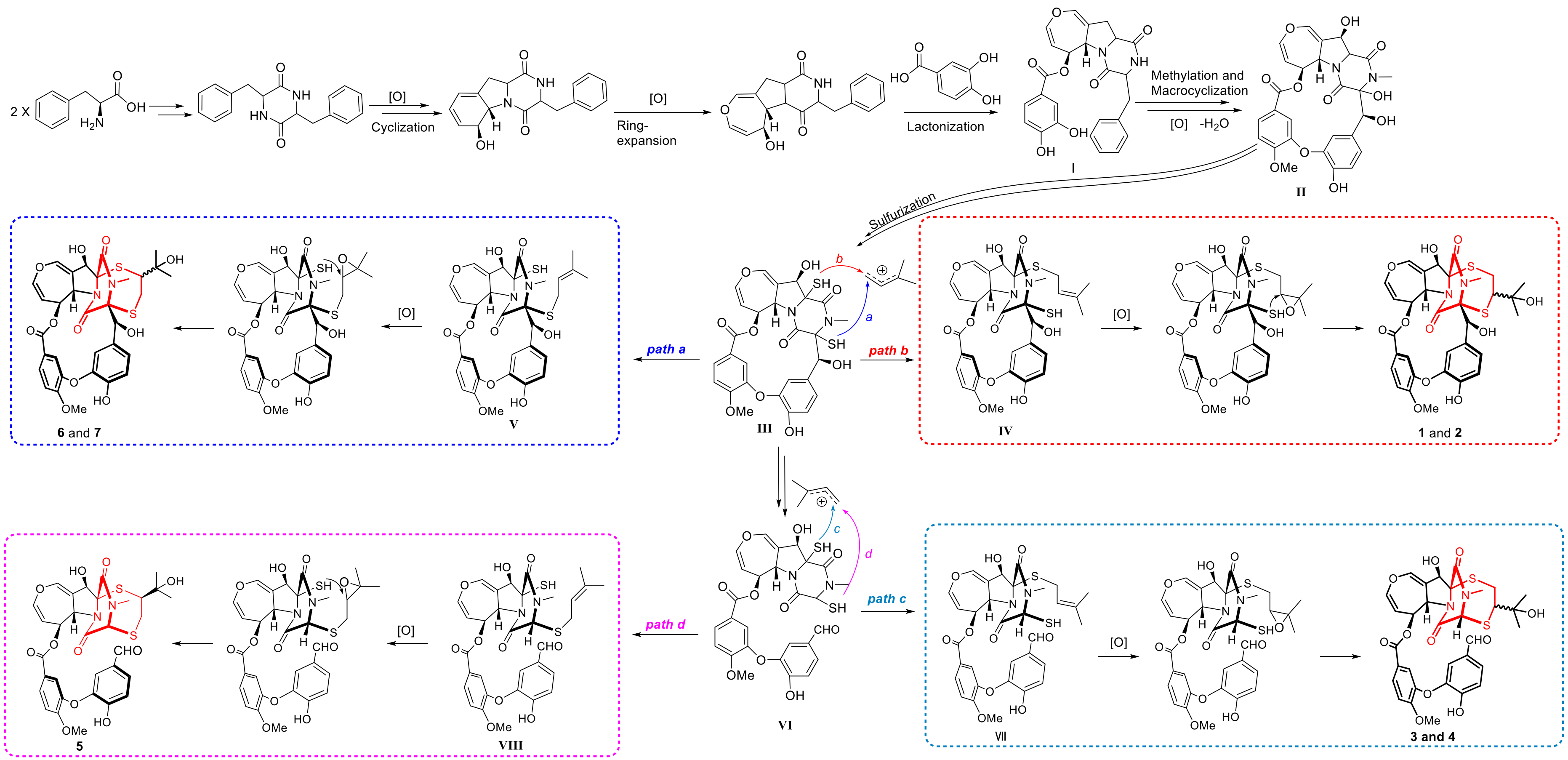

## Biological assays

### *Cell lines and cell culture*

Cultivatemouse leukemia cell line L1210 in RPMI-1640 (Procell, Wuhan, China) supplemented with 10% (v/v) fetal bovine serum and 1% (v/v) penicillin streptomycin solution. Cells were incubated at 37 °C in the presence of 5% $CO_2$ and used for less than six months after recovery.

### *Cytotoxicity Assay*

According to the manufacturer's instructions, measure cytotoxicity using Cell Counting Kit-8 (Topscience, Shanghai, China). Incubate cells at a density of 5000 cells/well into 96-well plates and incubated (with compound **6** or not) at a continuously diluted concentration. After 48 h, add 10 $\mu$L of CCK-8 reagent to the wells. Then incubate cells in the dark for 4 h. After that, the optical density value was measured at 450 nm using a plate reader (Bio-Tek Synergy HT, VT, USA). Calculate the 50% inhibitory concentration ($IC_{50}$) of compound **6** using the SPSS software.

### *Apoptosis analysis*

An Annexin V-FITC/PI apoptosis detection Kit (Keygen, Nanjing, China) was used to identify the apoptotic induction effect of compound **6**. Briefly, cells were exposed to vehicle control (DMSO, < 0.1%) or the compound **6** (10, 20 $\mu$M) for 24 h. Cells were harvested and washed with PBS and resuspended in a binding buffer with AnnexinV-FITC and PI. After 10 minutes, the cells were subjected to flow cytometry analysis (BD C6, USA). FlowJo_v10 software was used to analyze data.

### *Cell cycle analysis*

L1210 cells were seeded in 24-well plates at $2 \times 10^4$ cells per well. After being treated with compounds for 24 h, cells were harvested and washed by ice-cold PBS, then fixed with 70% ethanol at −20 °C for 12 h. Fixed cells were incubated with 50 $\mu$g/mL propidium iodide in the presence of 100 $\mu$g/mL RNAase and 0.2% Triton X-100 for at least 30 min. The DNA content of the cells was measured on BD C6

(USA) and cell cycle distribution was determined. FlowJo_v10 software was used to analyze data.

***EdU assays***

L1210 cells (1 × $10^5$ cells per well) were plated onto 6-well plates and treated with compounds for 48 h. The cell culture medium in each well was replaced with 1 ml of EdU medium (50 $\mu$M) and incubated for 2 h. Cells were harvested and cell proliferation activities were determined using a Cell-Light™ EdU Apollo® 567 In Vitro Flow Cytometry Kit (RiboBio, C10310-1). FlowJo_v10 software was used to analyze data.

***Measurement of mitochondrial membrane potential***

The fluorescent JC-1 assay kit (C2006, Beyotime, China) was used to quantify mitochondrial membrane potential (MMP). L1210 cells (2 × $10^4$ cells per well) were plated onto 24-well plates treated with compounds for 24 h and incubated in a culture medium supplemented with JC-1 for 20 min at 37 ℃ in the dark. We then washed the cells twice with PBS and measured JC-1 fluorescence with a fluorescence microscope or flow cytometry.

***Determination of ROS***

The Reactive Oxygen Species Assay Kit (Beyotime, S0033) was used to determine ROS. After the compound treatment, L1210 cells were incubated with DCFH-DA (diluted 1:1000) in a 37 °C incubator in the dark for 30 min and then harvested, washed, and resuspended in serum-free RPMI-1640. The fluorescence intensity was recorded by flow cytometry. FlowJo_v10 software was used to analyze data.

***Western Blot Analysis***

Cells were treated with compounds, and then lysed in RIPA (Beyotime Biotechnology, Shanghai, China) lysate buffer, the protein concentrations were quantified with BCA (bicinchoninic acid) protein assay kit. Protein concentrations were equalized before loading. Equal amounts of protein were denatured and subjected to electrophoresis in 10/12% SDS-PAGE gels followed by transfer to NC membrane. Immunoblotting was performed using indicated primary antibodies and

the image was developed with a two-color near-infrared fluorescence imaging system. Primary antibodies against PARP, cleaved caspase-3, Bcl-xl, Bak, PI3 KClass III, CyclinB1, CDC2, Phospho-CDC2, Phospho-CHK1 (Ser345), AKT, Phospho-AKT(Ser473), $\gamma$-H2A.X were purchased from Cell Signaling Technology (MA, USA). GAPDH and secondary antibodies were purchased from Proteintech (CHI, USA).

***Statistical analysis***

Statistical analysis was performed using the GraphPad Prism 6.0 software (San Diego, CA, USA). The results were obtained from at least three independent experiments and presented as the mean ± SD. ***$p < 0.01$, ****$p < 0.001$, unpaired two-tailed Student's t-test.

# ECD calculation Results

**Table S1.** Gibbs free energy[a] and equilibrium proporlation[b] of low-energy conformers of Compound **3**.

| Conformers | ΔG(a.u.) | P(%)/100 | G (a.u.) |
|---|---|---|---|
| **Compd 3**000002_tddft_ | 0.00592 | 0.12 | -2971.653642 |
| **Compd 3**000003_tddft_ | 0.0 | 61.17 | -2971.659563 |
| **Compd 3**000004_tddft_ | 0.00705 | 0.04 | -2971.652509 |
| Compd 3000006_tddft_ | 0.00662 | 0.06 | -2971.652944 |
| **Compd 3**000007_tddft_ | 0.00249 | 4.38 | -2971.657073 |
| **Compd 3**000008_tddft_ | 0.00055 | 34.25 | -2971.659015 |
| **Compd 3**000009_tddft_ | 0.00873 | 0.01 | -2971.650838 |

[a]wB97M-V/def2-TZVP, in a.u.

[b]From ΔG values at 298.15K.

**Table S2.** Cartesian coordinates for the low-energy reoptimized random research conformers of Compound **3** at B3LYP-D3(BJ)/6-31G* level of theory in methanol.

| **Compd 3**000002_en_ | | Standard Orientation (A.U.) | | | |
|---|---|---|---|---|---|
| Center number | Atomic number | Atomic Type | X | Y | Z |
| 0 | 6 | C | -6.593734 | -1.282383 | -5.572485 |
| 1 | 6 | C | -8.359608 | -1.558759 | -3.575749 |
| 2 | 6 | C | -8.776107 | -3.964472 | -2.543063 |
| 3 | 6 | C | -7.50582 | -6.06411 | -3.480853 |
| 4 | 6 | C | -5.780147 | -5.791017 | -5.474442 |
| 5 | 6 | C | -5.340154 | -3.382306 | -6.503659 |
| 6 | 8 | O | -6.159861 | 0.99043 | -6.755047 |
| 7 | 6 | C | -6.025707 | 3.226456 | -5.428346 |
| 8 | 6 | C | -4.580713 | 3.453575 | -3.242111 |
| 9 | 6 | C | -4.48268 | 5.763634 | -1.951292 |
| 10 | 6 | C | -5.786874 | 7.860699 | -2.920165 |
| 11 | 6 | C | -7.162257 | 7.644978 | -5.144541 |
| 12 | 6 | C | -7.344415 | 5.324558 | -6.41364 |
| 13 | 6 | C | -3.175551 | 6.060699 | 0.50944 |
| 14 | 8 | O | -1.151482 | 4.544234 | 0.783106 |
| 15 | 6 | C | 0.200865 | 4.54731 | 3.166093 |
| 16 | 6 | C | -1.404112 | 3.28389 | 5.249827 |
| 17 | 6 | C | 0.010091 | 2.993959 | 7.70563 |
| 18 | 6 | C | 1.456408 | 4.646372 | 8.962937 |
| 19 | 8 | O | 2.065119 | 7.0603 | 8.369491 |
| 20 | 6 | C | 1.884403 | 8.120771 | 6.021505 |
| 21 | 6 | C | 1.1364 | 7.137736 | 3.813208 |
| 22 | 7 | N | -2.008231 | 0.651683 | 4.587348 |
| 23 | 6 | C | -0.581905 | -1.134299 | 6.117242 |
| 24 | 6 | C | -0.321038 | 0.326749 | 8.653188 |
| 25 | 6 | C | -3.99411 | -0.008177 | 3.12381 |
| 26 | 6 | C | -4.153765 | -2.806411 | 2.527015 |
| 27 | 7 | N | -3.470386 | -4.418488 | 4.628928 |
| 28 | 6 | C | -1.872131 | -3.679875 | 6.475714 |
| 29 | 8 | O | -3.837535 | 7.570286 | 2.111865 |
| 30 | 8 | O | -1.376399 | -4.957577 | 8.354935 |
| 31 | 8 | O | -5.518512 | 1.495603 | 2.235964 |
| 32 | 8 | O | -8.755742 | 5.278436 | -8.547294 |
| 33 | 8 | O | -9.595878 | 0.506711 | -2.759609 |
| 34 | 6 | C | -4.576157 | -6.939913 | 4.649138 |
| 35 | 6 | C | -10.302288 | 3.140126 | -9.147662 |
| 36 | 8 | O | -2.584172 | 0.145173 | 10.061741 |
| 37 | 16 | S | 2.678668 | -1.715448 | 4.952944 |

| | | | | | |
|---|---|---|---|---|---|
| 38 | 16 | S | -2.516448 | -3.49743 | -0.48734 |
| 39 | 6 | C | 2.139106 | -3.607771 | 2.117582 |
| 40 | 6 | C | 0.699391 | -2.251204 | -0.010061 |
| 41 | 1 | H | -3.175044 | 4.34603 | 5.471855 |
| 42 | 6 | C | 2.152107 | -2.34015 | -2.590066 |
| 43 | 6 | C | 2.328039 | -5.008904 | -3.671772 |
| 44 | 6 | C | 0.97289 | -0.513578 | -4.494636 |
| 45 | 8 | O | 4.697081 | -1.570219 | -2.101149 |
| 46 | 6 | C | -4.357792 | -7.986487 | -6.37346 |
| 47 | 8 | O | -2.706505 | -7.923882 | -7.990261 |
| 48 | 1 | H | -10.140095 | -4.147938 | -1.002141 |
| 49 | 1 | H | -7.838234 | -7.927135 | -2.653969 |
| 50 | 1 | H | -3.987122 | -3.145262 | -8.04159 |
| 51 | 1 | H | -3.619644 | 1.78839 | -2.509653 |
| 52 | 1 | H | -5.724171 | 9.657081 | -1.909326 |
| 53 | 1 | H | -8.188692 | 9.255452 | -5.926793 |
| 54 | 1 | H | 1.800206 | 3.282286 | 2.774216 |
| 55 | 1 | H | 2.312618 | 4.121495 | 10.767905 |
| 56 | 1 | H | 2.552778 | 10.071086 | 6.115715 |
| 57 | 1 | H | 1.295379 | 8.426663 | 2.207979 |
| 58 | 1 | H | 1.30958 | -0.349817 | 9.751285 |
| 59 | 1 | H | -6.131417 | -3.184512 | 2.028838 |
| 60 | 1 | H | -10.68697 | 0.08708 | -1.343499 |
| 61 | 1 | H | -3.629355 | -8.089473 | 6.086613 |
| 62 | 1 | H | -6.612893 | -6.840476 | 5.071815 |
| 63 | 1 | H | -4.315815 | -7.817204 | 2.781456 |
| 64 | 1 | H | -11.100695 | 2.282472 | -7.429946 |
| 65 | 1 | H | -11.849745 | 3.869108 | -10.328064 |
| 66 | 1 | H | -9.24283 | 1.707292 | -10.216149 |
| 67 | 1 | H | -2.675077 | -1.568783 | 10.71788 |
| 68 | 1 | H | 4.081986 | -3.996964 | 1.494641 |
| 69 | 1 | H | 1.274932 | -5.42457 | 2.631368 |
| 70 | 1 | H | 0.515068 | -0.23755 | 0.441975 |
| 71 | 1 | H | 3.070998 | -6.354321 | -2.269368 |
| 72 | 1 | H | 0.474266 | -5.687376 | -4.319426 |
| 73 | 1 | H | 3.613825 | -5.001861 | -5.30764 |
| 74 | 1 | H | 0.925348 | 1.415167 | -3.712322 |
| 75 | 1 | H | -0.965591 | -1.053364 | -4.999155 |
| 76 | 1 | H | 2.119324 | -0.491996 | -6.230884 |
| 77 | 1 | H | 4.670462 | 0.178037 | -1.546463 |
| 78 | 1 | H | -4.869076 | -9.809752 | -5.442672 |

| **Compd 3**000003_en_ | | Standard Orientation (A.U.) | | | |
|---|---|---|---|---|---|
| Center number | Atomic number | Atomic Type | X | Y | Z |
| 0 | 6 | C | -6.24546 | -1.615438 | -5.783167 |
| 1 | 6 | C | -8.114926 | -1.711869 | -3.864193 |
| 2 | 6 | C | -8.683371 | -4.040042 | -2.731692 |
| 3 | 6 | C | -7.446031 | -6.233417 | -3.479859 |
| 4 | 6 | C | -5.59844 | -6.134289 | -5.378227 |
| 5 | 6 | C | -5.01494 | -3.805147 | -6.515744 |
| 6 | 8 | O | -5.698278 | 0.569325 | -7.078444 |
| 7 | 6 | C | -5.454668 | 2.854473 | -5.856915 |
| 8 | 6 | C | -4.175813 | 3.142564 | -3.590591 |
| 9 | 6 | C | -4.110009 | 5.527247 | -2.418269 |
| 10 | 6 | C | -5.276098 | 7.592538 | -3.591317 |
| 11 | 6 | C | -6.466641 | 7.323479 | -5.925677 |
| 12 | 6 | C | -6.592727 | 4.943382 | -7.077174 |
| 13 | 6 | C | -2.9824 | 5.931811 | 0.11132 |
| 14 | 8 | O | -1.052969 | 4.355762 | 0.633226 |
| 15 | 6 | C | 0.119888 | 4.4607 | 3.106432 |
| 16 | 6 | C | -1.68594 | 3.39783 | 5.137205 |
| 17 | 6 | C | -0.472577 | 3.218623 | 7.707448 |
| 18 | 6 | C | 0.943145 | 4.895324 | 8.967009 |
| 19 | 8 | O | 1.69688 | 7.239666 | 8.272418 |
| 20 | 6 | C | 1.736983 | 8.147796 | 5.855507 |
| 21 | 6 | C | 1.117329 | 7.048951 | 3.66218 |
| 22 | 7 | N | -2.350897 | 0.751856 | 4.598365 |
| 23 | 6 | C | -1.117112 | -0.980243 | 6.342611 |
| 24 | 6 | C | -0.98838 | 0.634731 | 8.793765 |
| 25 | 6 | C | -4.252335 | 0.071566 | 3.036077 |
| 26 | 6 | C | -4.480868 | -2.751504 | 2.603296 |
| 27 | 7 | N | -4.01669 | -4.249308 | 4.844048 |
| 28 | 6 | C | -2.526046 | -3.453104 | 6.756 |
| 29 | 8 | O | -3.69079 | 7.567148 | 1.565047 |
| 30 | 8 | O | -2.211577 | -4.63121 | 8.736582 |
| 31 | 8 | O | -5.645088 | 1.569304 | 1.944373 |
| 32 | 8 | O | -7.765722 | 4.446475 | -9.280955 |
| 33 | 8 | O | -9.291782 | 0.443581 | -3.214967 |
| 34 | 6 | C | -5.216128 | -6.726219 | 4.929895 |
| 35 | 6 | C | -8.978629 | 6.456266 | -10.590507 |
| 36 | 8 | O | -3.358829 | 0.634253 | 10.027418 |
| 37 | 16 | S | 2.194654 | -1.750266 | 5.46684 |
| 38 | 16 | S | -2.658147 | -3.692377 | -0.231289 |
| 39 | 6 | C | 1.785442 | -3.811727 | 2.728457 |

| | | | | | |
|---|---|---|---|---|---|
| 40 | 6 | C | 0.569441 | -2.552172 | 0.409596 |
| 41 | 1 | H | -3.423498 | 4.535396 | 5.157535 |
| 42 | 6 | C | 2.199059 | -2.891028 | -2.041494 |
| 43 | 6 | C | 2.325293 | -5.637138 | -2.916318 |
| 44 | 6 | C | 1.248192 | -1.160755 | -4.152662 |
| 45 | 8 | O | 4.737472 | -2.197498 | -1.419955 |
| 46 | 6 | C | -4.207668 | -8.423422 | -6.068022 |
| 47 | 8 | O | -2.467279 | -8.508433 | -7.587422 |
| 48 | 1 | H | -10.134938 | -4.086433 | -1.262035 |
| 49 | 1 | H | -7.895334 | -8.034142 | -2.573956 |
| 50 | 1 | H | -3.573 | -3.70436 | -7.986332 |
| 51 | 1 | H | -3.35303 | 1.491005 | -2.680762 |
| 52 | 1 | H | -5.23977 | 9.436661 | -2.668802 |
| 53 | 1 | H | -7.335972 | 8.961915 | -6.819441 |
| 54 | 1 | H | 1.687076 | 3.111988 | 2.914128 |
| 55 | 1 | H | 1.636781 | 4.459746 | 10.863248 |
| 56 | 1 | H | 2.478886 | 10.073525 | 5.877511 |
| 57 | 1 | H | 1.452684 | 8.222316 | 1.996653 |
| 58 | 1 | H | 0.522555 | -0.027547 | 10.058753 |
| 59 | 1 | H | -6.430164 | -3.091898 | 1.981586 |
| 60 | 1 | H | -10.413445 | 0.155795 | -1.78972 |
| 61 | 1 | H | -4.86071 | -7.721427 | 3.138773 |
| 62 | 1 | H | -4.415075 | -7.819156 | 6.494505 |
| 63 | 1 | H | -7.272021 | -6.532785 | 5.199731 |
| 64 | 1 | H | -9.792593 | 5.621537 | -12.306943 |
| 65 | 1 | H | -10.5035 | 7.294842 | -9.443593 |
| 66 | 1 | H | -7.617427 | 7.940353 | -11.126706 |
| 67 | 1 | H | -3.566709 | -1.015921 | 10.80752 |
| 68 | 1 | H | 3.750003 | -4.318873 | 2.284503 |
| 69 | 1 | H | 0.806097 | -5.553797 | 3.292152 |
| 70 | 1 | H | 0.449451 | -0.504324 | 0.703607 |
| 71 | 1 | H | 2.914604 | -6.903007 | -1.373661 |
| 72 | 1 | H | 0.489628 | -6.287455 | -3.639877 |
| 73 | 1 | H | 3.714578 | -5.80214 | -4.456466 |
| 74 | 1 | H | 1.232026 | 0.820217 | -3.51216 |
| 75 | 1 | H | -0.670271 | -1.657176 | -4.767038 |
| 76 | 1 | H | 2.51957 | -1.312808 | -5.792619 |
| 77 | 1 | H | 4.753869 | -0.417509 | -0.977652 |
| 78 | 1 | H | -4.835571 | -10.171583 | -5.066749 |

| **Compd 3**000004_en_ | | Standard Orientation (A.U.) | | | |
|---|---|---|---|---|---|
| Center number | Atomic number | Atomic Type | X | Y | Z |
| 0 | 6 | C | -8.910371 | -1.275278 | -4.737224 |
| 1 | 6 | C | -11.404146 | -0.359944 | -4.435713 |
| 2 | 6 | C | -13.066158 | -1.650162 | -2.824517 |
| 3 | 6 | C | -12.243783 | -3.782703 | -1.526156 |
| 4 | 6 | C | -9.754116 | -4.665114 | -1.802179 |
| 5 | 6 | C | -8.087495 | -3.383804 | -3.436342 |
| 6 | 8 | O | -7.328842 | -0.019814 | -6.387539 |
| 7 | 6 | C | -6.058891 | 2.071883 | -5.511403 |
| 8 | 6 | C | -5.8445 | 2.604895 | -2.945269 |
| 9 | 6 | C | -4.249389 | 4.5443 | -2.124585 |
| 10 | 6 | C | -2.930525 | 6.017774 | -3.878839 |
| 11 | 6 | C | -3.29428 | 5.587421 | -6.453264 |
| 12 | 6 | C | -4.838443 | 3.617357 | -7.310375 |
| 13 | 6 | C | -3.88704 | 4.849818 | 0.653157 |
| 14 | 8 | O | -1.469007 | 4.386899 | 1.278768 |
| 15 | 6 | C | -0.641683 | 4.464125 | 3.878733 |
| 16 | 6 | C | -1.797494 | 2.306707 | 5.500966 |
| 17 | 6 | C | -0.376473 | 2.101247 | 7.961751 |
| 18 | 6 | C | 0.200661 | 3.914356 | 9.629013 |
| 19 | 8 | O | -0.376252 | 6.411242 | 9.566368 |
| 20 | 6 | C | -1.013798 | 7.738069 | 7.444499 |
| 21 | 6 | C | -1.103451 | 7.013078 | 5.021344 |
| 22 | 7 | N | -1.469459 | -0.217997 | 4.391676 |
| 23 | 6 | C | 0.583137 | -1.601256 | 5.588162 |
| 24 | 6 | C | 0.503045 | -0.581949 | 8.350698 |
| 25 | 6 | C | -3.187095 | -1.296845 | 2.841285 |
| 26 | 6 | C | -2.503948 | -3.913336 | 1.898287 |
| 27 | 7 | N | -1.180669 | -5.453474 | 3.727441 |
| 28 | 6 | C | 0.296298 | -4.466213 | 5.553561 |
| 29 | 8 | O | -5.530391 | 5.375897 | 2.157007 |
| 30 | 8 | O | 1.424799 | -5.734472 | 7.144892 |
| 31 | 8 | O | -5.137243 | -0.254865 | 2.145763 |
| 32 | 8 | O | -4.987807 | 3.230621 | -9.844079 |
| 33 | 8 | O | -12.030388 | 1.727486 | -5.733457 |
| 34 | 6 | C | -1.384173 | -8.180838 | 3.395337 |
| 35 | 6 | C | -7.378821 | 2.660324 | -10.97315 |
| 36 | 8 | O | -1.318658 | -1.910349 | 9.785886 |
| 37 | 16 | S | 3.715952 | -0.803487 | 4.236165 |
| 38 | 16 | S | -0.972024 | -3.705473 | -1.232338 |
| 39 | 6 | C | 3.64236 | -2.375064 | 1.155152 |

| | | | | | |
|---|---|---|---|---|---|
| 40 | 6 | C | 1.642346 | -1.444577 | -0.740306 |
| 41 | 1 | H | -3.827803 | 2.658289 | 5.740733 |
| 42 | 6 | C | 2.82899 | -0.811549 | -3.376735 |
| 43 | 6 | C | 4.077199 | -3.099812 | -4.613657 |
| 44 | 6 | C | 0.86376 | 0.385064 | -5.12663 |
| 45 | 8 | O | 4.817209 | 0.966058 | -2.932813 |
| 46 | 6 | C | -8.919311 | -6.876987 | -0.367969 |
| 47 | 8 | O | -6.764842 | -7.723109 | -0.370551 |
| 48 | 1 | H | -14.997683 | -0.950105 | -2.609332 |
| 49 | 1 | H | -13.542144 | -4.778874 | -0.265238 |
| 50 | 1 | H | -6.141975 | -4.024189 | -3.669277 |
| 51 | 1 | H | -6.78482 | 1.410841 | -1.561182 |
| 52 | 1 | H | -1.652966 | 7.507748 | -3.242857 |
| 53 | 1 | H | -2.345029 | 6.749584 | -7.870284 |
| 54 | 1 | H | 1.386643 | 4.073057 | 3.688141 |
| 55 | 1 | H | 1.232727 | 3.464153 | 11.360671 |
| 56 | 1 | H | -1.404626 | 9.696423 | 7.965464 |
| 57 | 1 | H | -1.568682 | 8.517818 | 3.68665 |
| 58 | 1 | H | 2.379462 | -0.691302 | 9.241001 |
| 59 | 1 | H | -4.287651 | -4.85978 | 1.426883 |
| 60 | 1 | H | -13.767689 | 2.213958 | -5.387318 |
| 61 | 1 | H | -0.704325 | -8.702627 | 1.499035 |
| 62 | 1 | H | -0.22782 | -9.124452 | 4.828413 |
| 63 | 1 | H | -3.367894 | -8.776281 | 3.579905 |
| 64 | 1 | H | -8.94338 | 3.538856 | -9.919552 |
| 65 | 1 | H | -7.322301 | 3.44787 | -12.896202 |
| 66 | 1 | H | -7.695783 | 0.608535 | -11.082928 |
| 67 | 1 | H | -0.629528 | -3.583667 | 10.110126 |
| 68 | 1 | H | 5.528927 | -1.899354 | 0.426084 |
| 69 | 1 | H | 3.581411 | -4.433846 | 1.421157 |
| 70 | 1 | H | 0.803107 | 0.332376 | -0.070506 |
| 71 | 1 | H | 5.539171 | -3.928469 | -3.388201 |
| 72 | 1 | H | 2.678273 | -4.573984 | -5.049564 |
| 73 | 1 | H | 4.983414 | -2.515401 | -6.392904 |
| 74 | 1 | H | -0.063689 | 1.983952 | -4.179371 |
| 75 | 1 | H | -0.607997 | -0.960075 | -5.70823 |
| 76 | 1 | H | 1.810745 | 1.094076 | -6.838002 |
| 77 | 1 | H | 4.068664 | 2.513477 | -2.292157 |
| 78 | 1 | H | -10.416694 | -7.789769 | 0.801677 |

| **Compd 3**000006_en_ | | Standard Orientation (A.U.) | | | |
|---|---|---|---|---|---|
| Center number | Atomic number | Atomic Type | X | Y | Z |
| 0 | 6 | C | -12.038878 | 0.105277 | -7.124933 |
| 1 | 6 | C | -11.422066 | 2.288933 | -8.547056 |
| 2 | 6 | C | -12.512001 | 2.626043 | -10.937769 |
| 3 | 6 | C | -14.20942 | 0.858928 | -11.891808 |
| 4 | 6 | C | -14.840643 | -1.288071 | -10.467467 |
| 5 | 6 | C | -13.728989 | -1.646498 | -8.077432 |
| 6 | 8 | O | -11.105072 | -0.273921 | -4.725893 |
| 7 | 6 | C | -8.594401 | 0.095554 | -4.140611 |
| 8 | 6 | C | -8.033636 | 1.289853 | -1.881918 |
| 9 | 6 | C | -5.510721 | 1.615551 | -1.124725 |
| 10 | 6 | C | -3.566297 | 0.727015 | -2.681117 |
| 11 | 6 | C | -4.118518 | -0.481977 | -4.954533 |
| 12 | 6 | C | -6.634382 | -0.834845 | -5.707827 |
| 13 | 6 | C | -5.00637 | 2.839742 | 1.336743 |
| 14 | 8 | O | -2.527136 | 3.281477 | 1.715023 |
| 15 | 6 | C | -1.666561 | 4.279328 | 4.101154 |
| 16 | 6 | C | -2.037807 | 2.334564 | 6.248618 |
| 17 | 6 | C | -0.845951 | 3.197252 | 8.686425 |
| 18 | 6 | C | -0.94495 | 5.443471 | 9.848058 |
| 19 | 8 | O | -2.164124 | 7.585491 | 9.157893 |
| 20 | 6 | C | -3.026255 | 8.116217 | 6.782849 |
| 21 | 6 | C | -2.835143 | 6.804044 | 4.628251 |
| 22 | 7 | N | -0.701071 | -0.051202 | 5.729236 |
| 23 | 6 | C | 1.514393 | -0.350777 | 7.326234 |
| 24 | 6 | C | 0.71121 | 1.073083 | 9.768966 |
| 25 | 6 | C | -1.675269 | -1.923996 | 4.297747 |
| 26 | 6 | C | 0.040659 | -4.200175 | 3.948998 |
| 27 | 7 | N | 1.526154 | -4.821022 | 6.158136 |
| 28 | 6 | C | 2.219769 | -3.081219 | 7.890293 |
| 29 | 8 | O | -6.636359 | 3.419229 | 2.853318 |
| 30 | 8 | O | 3.410835 | -3.576857 | 9.825436 |
| 31 | 8 | O | -3.751617 | -1.84014 | 3.274576 |
| 32 | 8 | O | -7.355776 | -2.016085 | -7.841415 |
| 33 | 8 | O | -9.797882 | 3.951956 | -7.517264 |
| 34 | 6 | C | 2.336731 | -7.441946 | 6.398307 |
| 35 | 6 | C | -5.486935 | -2.909591 | -9.555147 |
| 36 | 8 | O | -0.839305 | -0.485595 | 11.288542 |
| 37 | 16 | S | 4.351658 | 1.270459 | 6.064228 |
| 38 | 16 | S | 1.827694 | -3.966233 | 0.950893 |
| 39 | 6 | C | 5.353872 | -0.818764 | 3.507044 |

| | | | | | |
|---|---|---|---|---|---|
| 40 | 6 | C | 3.607117 | -0.969456 | 1.184433 |
| 41 | 1 | H | -4.065052 | 1.932118 | 6.447358 |
| 42 | 6 | C | 5.136623 | -0.635507 | -1.321548 |
| 43 | 6 | C | 7.145716 | -2.68792 | -1.681429 |
| 44 | 6 | C | 3.400751 | -0.481204 | -3.630181 |
| 45 | 8 | O | 6.332803 | 1.771469 | -0.987565 |
| 46 | 6 | C | -16.631531 | -3.142314 | -11.481027 |
| 47 | 8 | O | -17.303986 | -5.06608 | -10.39339 |
| 48 | 1 | H | -12.012596 | 4.31474 | -12.019267 |
| 49 | 1 | H | -15.058954 | 1.142744 | -13.753395 |
| 50 | 1 | H | -14.183124 | -3.318588 | -6.957981 |
| 51 | 1 | H | -9.581384 | 1.976215 | -0.706234 |
| 52 | 1 | H | -1.607552 | 0.950217 | -2.101534 |
| 53 | 1 | H | -2.579283 | -1.17935 | -6.131426 |
| 54 | 1 | H | 0.379226 | 4.465553 | 3.793877 |
| 55 | 1 | H | 0.018053 | 5.727684 | 11.653079 |
| 56 | 1 | H | -3.932605 | 9.970329 | 6.796464 |
| 57 | 1 | H | -3.622325 | 7.757097 | 2.974202 |
| 58 | 1 | H | 2.37013 | 1.745009 | 10.826904 |
| 59 | 1 | H | -1.221519 | -5.798058 | 3.545895 |
| 60 | 1 | H | -9.500678 | 5.357285 | -8.661271 |
| 61 | 1 | H | 0.700733 | -8.675058 | 6.769532 |
| 62 | 1 | H | 3.251017 | -8.044211 | 4.629935 |
| 63 | 1 | H | 3.687054 | -7.593592 | 7.959432 |
| 64 | 1 | H | -4.283159 | -4.360613 | -8.668529 |
| 65 | 1 | H | -6.520602 | -3.7509 | -11.144991 |
| 66 | 1 | H | -4.298861 | -1.346798 | -10.252615 |
| 67 | 1 | H | 0.25195 | -1.755841 | 12.045369 |
| 68 | 1 | H | 7.138875 | 0.07697 | 2.93946 |
| 69 | 1 | H | 5.827953 | -2.691414 | 4.268589 |
| 70 | 1 | H | 2.234219 | 0.588886 | 1.231226 |
| 71 | 1 | H | 8.290195 | -2.243883 | -3.364312 |
| 72 | 1 | H | 8.424656 | -2.802615 | -0.046304 |
| 73 | 1 | H | 6.28891 | -4.559845 | -1.974456 |
| 74 | 1 | H | 4.551664 | -0.135267 | -5.330851 |
| 75 | 1 | H | 2.07562 | 1.104859 | -3.416345 |
| 76 | 1 | H | 2.318397 | -2.228017 | -3.93684 |
| 77 | 1 | H | 7.477722 | 2.025599 | -2.395763 |
| 78 | 1 | H | -17.385503 | -2.679475 | -13.397179 |

| **Compd 3**000007_en_ | | Standard Orientation (A.U.) | | | |
|---|---|---|---|---|---|
| Center number | Atomic number | Atomic Type | X | Y | Z |
| 0 | 6 | C | -10.919429 | 0.041366 | -4.229912 |
| 1 | 6 | C | -13.203252 | -0.625573 | -5.464612 |
| 2 | 6 | C | -13.969373 | -3.156593 | -5.506496 |
| 3 | 6 | C | -12.495909 | -5.003366 | -4.348657 |
| 4 | 6 | C | -10.239842 | -4.341621 | -3.120943 |
| 5 | 6 | C | -9.455849 | -1.791462 | -3.070145 |
| 6 | 8 | O | -10.342552 | 2.576275 | -4.249074 |
| 7 | 6 | C | -7.838062 | 3.235721 | -3.939189 |
| 8 | 6 | C | -6.99244 | 4.17055 | -1.653425 |
| 9 | 6 | C | -4.417543 | 4.729402 | -1.329585 |
| 10 | 6 | C | -2.757341 | 4.425714 | -3.363454 |
| 11 | 6 | C | -3.621101 | 3.556803 | -5.697222 |
| 12 | 6 | C | -6.168748 | 2.90282 | -6.000865 |
| 13 | 6 | C | -3.53646 | 5.349335 | 1.251201 |
| 14 | 8 | O | -1.069192 | 4.790728 | 1.557548 |
| 15 | 6 | C | 0.059955 | 4.832527 | 4.035618 |
| 16 | 6 | C | -1.065558 | 2.741885 | 5.752619 |
| 17 | 6 | C | 0.465404 | 2.441573 | 8.134438 |
| 18 | 6 | C | 1.33107 | 4.203732 | 9.731385 |
| 19 | 8 | O | 1.027945 | 6.746201 | 9.668406 |
| 20 | 6 | C | 0.316695 | 8.121952 | 7.600898 |
| 21 | 6 | C | -0.07698 | 7.406651 | 5.205708 |
| 22 | 7 | N | -0.904627 | 0.216839 | 4.601736 |
| 23 | 6 | C | 1.052863 | -1.322691 | 5.764052 |
| 24 | 6 | C | 1.076585 | -0.313396 | 8.527916 |
| 25 | 6 | C | -2.668367 | -0.718916 | 3.01588 |
| 26 | 6 | C | -2.130972 | -3.341132 | 2.004087 |
| 27 | 7 | N | -0.96904 | -5.0075 | 3.831202 |
| 28 | 6 | C | 0.548119 | -4.158206 | 5.696647 |
| 29 | 8 | O | -4.870159 | 6.173442 | 2.93047 |
| 30 | 8 | O | 1.553593 | -5.528005 | 7.285917 |
| 31 | 8 | O | -4.571503 | 0.434486 | 2.363533 |
| 32 | 8 | O | -7.193745 | 1.935163 | -8.115359 |
| 33 | 8 | O | -14.520123 | 1.258631 | -6.544432 |
| 34 | 6 | C | -1.372193 | -7.706736 | 3.452768 |
| 35 | 6 | C | -5.589521 | 1.36973 | -10.199685 |
| 36 | 8 | O | -0.868962 | -1.460409 | 9.957802 |
| 37 | 16 | S | 4.23159 | -0.755301 | 4.406717 |
| 38 | 16 | S | -0.584256 | -3.201837 | -1.124495 |
| 39 | 6 | C | 4.112653 | -2.483074 | 1.421734 |

| | | | | | |
|---|---|---|---|---|---|
| 40 | 6 | C | 2.383311 | -1.412803 | -0.652638 |
| 41 | 1 | H | -3.062949 | 3.176488 | 6.114816 |
| 42 | 6 | C | 3.811101 | -1.313588 | -3.254564 |
| 43 | 6 | C | 4.64492 | -3.924413 | -4.160793 |
| 44 | 6 | C | 2.239195 | 0.028627 | -5.272795 |
| 45 | 8 | O | 6.088849 | 0.084953 | -2.854977 |
| 46 | 6 | C | -8.754103 | -6.319386 | -1.88675 |
| 47 | 8 | O | -6.792197 | -5.989753 | -0.705042 |
| 48 | 1 | H | -15.729224 | -3.646735 | -6.471401 |
| 49 | 1 | H | -13.098291 | -6.978709 | -4.389203 |
| 50 | 1 | H | -7.708289 | -1.278562 | -2.10314 |
| 51 | 1 | H | -8.313559 | 4.343035 | -0.080962 |
| 52 | 1 | H | -0.75906 | 4.863316 | -3.123237 |
| 53 | 1 | H | -2.296082 | 3.346992 | -7.2584 |
| 54 | 1 | H | 2.025594 | 4.300265 | 3.632381 |
| 55 | 1 | H | 2.413131 | 3.660422 | 11.404775 |
| 56 | 1 | H | 0.161211 | 10.108806 | 8.137091 |
| 57 | 1 | H | -0.535579 | 8.945903 | 3.909118 |
| 58 | 1 | H | 2.933063 | -0.607925 | 9.416683 |
| 59 | 1 | H | -3.970316 | -4.135178 | 1.470519 |
| 60 | 1 | H | -16.038431 | 0.596721 | -7.337385 |
| 61 | 1 | H | -0.382248 | -8.344271 | 1.735577 |
| 62 | 1 | H | -0.646235 | -8.74138 | 5.091283 |
| 63 | 1 | H | -3.40437 | -8.07785 | 3.219825 |
| 64 | 1 | H | -4.701406 | 3.095097 | -10.957898 |
| 65 | 1 | H | -4.111341 | 0.004567 | -9.657814 |
| 66 | 1 | H | -6.806002 | 0.528558 | -11.654362 |
| 67 | 1 | H | -0.350612 | -3.195395 | 10.273746 |
| 68 | 1 | H | 6.079854 | -2.290333 | 0.782305 |
| 69 | 1 | H | 3.781933 | -4.500825 | 1.782388 |
| 70 | 1 | H | 1.864689 | 0.549351 | -0.1984 |
| 71 | 1 | H | 5.815698 | -4.892272 | -2.740594 |
| 72 | 1 | H | 3.009669 | -5.131512 | -4.591158 |
| 73 | 1 | H | 5.778577 | -3.716273 | -5.89268 |
| 74 | 1 | H | 1.753919 | 1.946785 | -4.638267 |
| 75 | 1 | H | 0.473403 | -0.976231 | -5.706697 |
| 76 | 1 | H | 3.353799 | 0.179728 | -7.022999 |
| 77 | 1 | H | 5.634254 | 1.806147 | -2.411701 |
| 78 | 1 | H | -9.549175 | -8.262215 | -2.080392 |

| **Compd 3**000008_en_ | | Standard Orientation (A.U.) | | | |
|---|---|---|---|---|---|
| Center number | Atomic number | Atomic Type | X | Y | Z |
| 0 | 6 | C | -10.20759 | 0.492404 | -3.72617 |
| 1 | 6 | C | -12.686399 | 0.775481 | -2.755689 |
| 2 | 6 | C | -13.761146 | -1.177422 | -1.331881 |
| 3 | 6 | C | -12.378252 | -3.365889 | -0.873719 |
| 4 | 6 | C | -9.921618 | -3.640894 | -1.842415 |
| 5 | 6 | C | -8.836458 | -1.689066 | -3.296559 |
| 6 | 8 | O | -9.400053 | 2.493274 | -5.173853 |
| 7 | 6 | C | -6.949929 | 3.356541 | -5.080614 |
| 8 | 6 | C | -5.596594 | 3.4673 | -2.830184 |
| 9 | 6 | C | -3.182758 | 4.559267 | -2.781754 |
| 10 | 6 | C | -2.166974 | 5.599859 | -5.00191 |
| 11 | 6 | C | -3.516554 | 5.475028 | -7.244284 |
| 12 | 6 | C | -5.902399 | 4.313077 | -7.343772 |
| 13 | 6 | C | -1.638557 | 4.580044 | -0.458629 |
| 14 | 8 | O | -2.912556 | 3.761899 | 1.568573 |
| 15 | 6 | C | -1.700848 | 3.822062 | 4.006835 |
| 16 | 6 | C | -2.481917 | 1.422089 | 5.472288 |
| 17 | 6 | C | -1.171526 | 1.336522 | 8.005366 |
| 18 | 6 | C | -0.961975 | 3.114291 | 9.793309 |
| 19 | 8 | O | -1.903378 | 5.496142 | 9.833923 |
| 20 | 6 | C | -2.669265 | 6.806359 | 7.751301 |
| 21 | 6 | C | -2.554583 | 6.197005 | 5.297218 |
| 22 | 7 | N | -1.636442 | -0.940233 | 4.270951 |
| 23 | 6 | C | 0.54943 | -2.00537 | 5.553973 |
| 24 | 6 | C | 0.091058 | -1.192068 | 8.343863 |
| 25 | 6 | C | -2.977284 | -2.172618 | 2.480456 |
| 26 | 6 | C | -1.787845 | -4.614757 | 1.537938 |
| 27 | 7 | N | -0.415974 | -6.005818 | 3.452093 |
| 28 | 6 | C | 0.784987 | -4.868443 | 5.386131 |
| 29 | 8 | O | 0.573606 | 5.242654 | -0.375691 |
| 30 | 8 | O | 2.025221 | -5.996547 | 7.000729 |
| 31 | 8 | O | -4.979351 | -1.401922 | 1.604393 |
| 32 | 8 | O | -7.067101 | 4.27467 | -9.610403 |
| 33 | 8 | O | -14.002695 | 2.898388 | -3.201508 |
| 34 | 6 | C | -0.198648 | -8.722673 | 3.061056 |
| 35 | 6 | C | -8.695681 | 2.234193 | -10.324043 |
| 36 | 8 | O | -1.606388 | -2.874134 | 9.541443 |
| 37 | 16 | S | 3.55013 | -0.540891 | 4.509567 |
| 38 | 16 | S | -0.003453 | -4.075797 | -1.414212 |
| 39 | 6 | C | 4.055638 | -1.888006 | 1.367196 |

| | | | | | |
|---|---|---|---|---|---|
| 40 | 6 | C | 2.047409 | -1.35658 | -0.666162 |
| 41 | 1 | H | -4.554901 | 1.380146 | 5.612749 |
| 42 | 6 | C | 3.274242 | -0.372512 | -3.175825 |
| 43 | 6 | C | 5.352723 | -2.117608 | -4.159935 |
| 44 | 6 | C | 1.283389 | 0.048708 | -5.238166 |
| 45 | 8 | O | 4.467802 | 1.969684 | -2.561104 |
| 46 | 6 | C | -8.497463 | -5.946294 | -1.299062 |
| 47 | 8 | O | -6.353524 | -6.389756 | -2.047008 |
| 48 | 1 | H | -15.671393 | -0.928506 | -0.592342 |
| 49 | 1 | H | -13.200706 | -4.888665 | 0.254231 |
| 50 | 1 | H | -6.919516 | -1.89851 | -4.027536 |
| 51 | 1 | H | -6.425003 | 2.698249 | -1.110666 |
| 52 | 1 | H | -0.289293 | 6.448621 | -4.961143 |
| 53 | 1 | H | -2.744982 | 6.234103 | -9.001024 |
| 54 | 1 | H | 0.351395 | 3.773564 | 3.706155 |
| 55 | 1 | H | 0.046245 | 2.716818 | 11.551267 |
| 56 | 1 | H | -3.393941 | 8.648341 | 8.336179 |
| 57 | 1 | H | -3.239644 | 7.665483 | 4.015832 |
| 58 | 1 | H | 1.883964 | -1.066972 | 9.388921 |
| 59 | 1 | H | -3.349555 | -5.80313 | 0.86591 |
| 60 | 1 | H | -12.964222 | 4.049744 | -4.196804 |
| 61 | 1 | H | -2.084971 | -9.600141 | 3.113214 |
| 62 | 1 | H | 0.667251 | -9.096654 | 1.205847 |
| 63 | 1 | H | 0.989134 | -9.529704 | 4.550988 |
| 64 | 1 | H | -10.640991 | 2.545384 | -9.665736 |
| 65 | 1 | H | -8.669213 | 2.189824 | -12.400878 |
| 66 | 1 | H | -7.991225 | 0.417892 | -9.592664 |
| 67 | 1 | H | -0.682323 | -4.428154 | 9.870811 |
| 68 | 1 | H | 5.816212 | -0.92175 | 0.833577 |
| 69 | 1 | H | 4.486236 | -3.913542 | 1.529773 |
| 70 | 1 | H | 0.796004 | 0.17167 | -0.03959 |
| 71 | 1 | H | 4.603405 | -4.016571 | -4.556334 |
| 72 | 1 | H | 6.129914 | -1.342843 | -5.928203 |
| 73 | 1 | H | 6.918274 | -2.301959 | -2.805261 |
| 74 | 1 | H | 2.11011 | 1.247257 | -6.723969 |
| 75 | 1 | H | -0.397844 | 0.998163 | -4.485286 |
| 76 | 1 | H | 0.673848 | -1.734663 | -6.113684 |
| 77 | 1 | H | 3.174821 | 3.146812 | -1.971139 |
| 78 | 1 | H | -9.516041 | -7.347641 | -0.097189 |

| **Compd 3**000009_en_ | | Standard Orientation (A.U.) | | | |
|---|---|---|---|---|---|
| Center number | Atomic number | Atomic Type | X | Y | Z |
| 0 | 6 | C | -10.777545 | 0.075644 | -4.656623 |
| 1 | 6 | C | -13.31266 | -0.423815 | -5.378384 |
| 2 | 6 | C | -14.389412 | -2.775216 | -4.835537 |
| 3 | 6 | C | -12.973983 | -4.619738 | -3.60275 |
| 4 | 6 | C | -10.463465 | -4.13306 | -2.904604 |
| 5 | 6 | C | -9.362136 | -1.760941 | -3.443878 |
| 6 | 8 | O | -9.924324 | 2.430131 | -5.322656 |
| 7 | 6 | C | -7.457031 | 3.110897 | -4.810891 |
| 8 | 6 | C | -6.812776 | 3.803233 | -2.359777 |
| 9 | 6 | C | -4.34459 | 4.528118 | -1.761661 |
| 10 | 6 | C | -2.530674 | 4.644554 | -3.691709 |
| 11 | 6 | C | -3.189153 | 4.028768 | -6.153462 |
| 12 | 6 | C | -5.644517 | 3.208829 | -6.769774 |
| 13 | 6 | C | -3.729735 | 4.993949 | 0.927068 |
| 14 | 8 | O | -1.269558 | 4.55526 | 1.416233 |
| 15 | 6 | C | -0.355461 | 4.580573 | 3.984408 |
| 16 | 6 | C | -1.526944 | 2.402947 | 5.547805 |
| 17 | 6 | C | -0.240766 | 2.126443 | 8.075127 |
| 18 | 6 | C | 0.392683 | 3.889824 | 9.776819 |
| 19 | 8 | O | 0.005475 | 6.421193 | 9.718701 |
| 20 | 6 | C | -0.572682 | 7.803985 | 7.61547 |
| 21 | 6 | C | -0.71621 | 7.118472 | 5.183653 |
| 22 | 7 | N | -1.12054 | -0.093895 | 4.383838 |
| 23 | 6 | C | 0.796355 | -1.545119 | 5.728629 |
| 24 | 6 | C | 0.392846 | -0.626648 | 8.489149 |
| 25 | 6 | C | -2.672351 | -1.088227 | 2.620869 |
| 26 | 6 | C | -1.90953 | -3.669922 | 1.657951 |
| 27 | 7 | N | -0.858986 | -5.298023 | 3.57985 |
| 28 | 6 | C | 0.561502 | -4.410734 | 5.511082 |
| 29 | 8 | O | -5.250602 | 5.633427 | 2.525434 |
| 30 | 8 | O | 1.680516 | -5.772121 | 7.017264 |
| 31 | 8 | O | -4.544202 | -0.01062 | 1.777355 |
| 32 | 8 | O | -6.037049 | 2.658257 | -9.225384 |
| 33 | 8 | O | -14.542369 | 1.441971 | -6.588987 |
| 34 | 6 | C | -1.017703 | -8.012078 | 3.126667 |
| 35 | 6 | C | -8.085883 | 1.117548 | -10.077919 |
| 36 | 8 | O | -1.576665 | -1.992886 | 9.674171 |
| 37 | 16 | S | 4.040085 | -0.670267 | 4.73808 |
| 38 | 16 | S | -0.035589 | -3.386792 | -1.279539 |
| 39 | 6 | C | 4.326201 | -2.303538 | 1.70826 |

| | | | | | |
|---|---|---|---|---|---|
| 40 | 6 | C | 2.672419 | -1.322262 | -0.477453 |
| 41 | 1 | H | -3.570717 | 2.726081 | 5.708485 |
| 42 | 6 | C | 4.270084 | -0.970348 | -2.937696 |
| 43 | 6 | C | 5.445078 | -3.445574 | -3.867729 |
| 44 | 6 | C | 2.731461 | 0.296874 | -5.031972 |
| 45 | 8 | O | 6.226969 | 0.737313 | -2.16515 |
| 46 | 6 | C | -9.019402 | -6.120095 | -1.636446 |
| 47 | 8 | O | -6.816949 | -5.947458 | -0.949334 |
| 48 | 1 | H | -16.343552 | -3.131123 | -5.403048 |
| 49 | 1 | H | -13.815478 | -6.458685 | -3.183083 |
| 50 | 1 | H | -7.410969 | -1.401049 | -2.885886 |
| 51 | 1 | H | -8.253997 | 3.698005 | -0.88926 |
| 52 | 1 | H | -0.602443 | 5.224329 | -3.256992 |
| 53 | 1 | H | -1.801226 | 4.117503 | -7.677984 |
| 54 | 1 | H | 1.659675 | 4.145315 | 3.739505 |
| 55 | 1 | H | 1.314582 | 3.354381 | 11.545924 |
| 56 | 1 | H | -0.862367 | 9.772418 | 8.163914 |
| 57 | 1 | H | -1.121655 | 8.66285 | 3.874893 |
| 58 | 1 | H | 2.090082 | -0.921223 | 9.641154 |
| 59 | 1 | H | -3.643573 | -4.538466 | 0.929413 |
| 60 | 1 | H | -16.250299 | 0.911538 | -7.006432 |
| 61 | 1 | H | 0.45149 | -8.628019 | 1.784189 |
| 62 | 1 | H | -0.766809 | -9.023805 | 4.916463 |
| 63 | 1 | H | -2.883374 | -8.450783 | 2.3261 |
| 64 | 1 | H | -8.153558 | -0.680846 | -9.03362 |
| 65 | 1 | H | -9.910075 | 2.096492 | -9.923311 |
| 66 | 1 | H | -7.683798 | 0.71995 | -12.07555 |
| 67 | 1 | H | -3.17358 | -1.574599 | 8.872618 |
| 68 | 1 | H | 6.315667 | -1.933383 | 1.243387 |
| 69 | 1 | H | 4.143085 | -4.352498 | 1.992573 |
| 70 | 1 | H | 1.923055 | 0.556649 | -0.004653 |
| 71 | 1 | H | 6.602212 | -4.345711 | -2.393189 |
| 72 | 1 | H | 3.992663 | -4.80297 | -4.477589 |
| 73 | 1 | H | 6.674981 | -3.055067 | -5.502828 |
| 74 | 1 | H | 2.009106 | 2.123293 | -4.362582 |
| 75 | 1 | H | 1.126235 | -0.86078 | -5.664133 |
| 76 | 1 | H | 3.960135 | 0.641938 | -6.677859 |
| 77 | 1 | H | 7.388398 | 0.919288 | -3.570985 |
| 78 | 1 | H | -10.084881 | -7.909819 | -1.313337 |

**Table S3**. Gibbs free energy[a] and equilibrium proporlation[b] of low-energy conformers of compound **5**.

| Conformers | ΔG(a.u.) | P(%)/100 | G(a.u.) |
|---|---|---|---|
| **Compd** 5000001_tddft_ | 0.00017 | 33.67 | -2971.64108 |
| **Compd** 5000002_tddft_ | 0.00301 | 1.66 | -2971.638241 |
| **Compd** 5000003_tddft_ | 0.00077 | 17.88 | -2971.640482 |
| **Compd** 5000005_tddft_ | 0.0 | 40.33 | -2971.641251 |
| **Compd** 5000006_tddft_ | 0.00232 | 3.45 | -2971.63893 |
| **Compd** 5000007_tddft_ | 0.00409 | 0.53 | -2971.637166 |
| **Compd** 5000009_tddft_ | 0.00263 | 2.48 | -2971.638616 |

[a]wB97M-V/def2-TZVP, in a.u.

[b]From ΔG values at 298.15K.

**Table S4.** Cartesian coordinates for the low-energy reoptimized random research conformers of **Compd 5** at B3LYP-D3(BJ)/6-31G* level of theory in methanol.

| **Compd** 5000001_en_ | | Standard Orientation (A.U.) | | | |
|---|---|---|---|---|---|
| Center number | Atomic number | Atomic Type | X | Y | Z |
| 0 | 7 | N | 9.449356 | 1.36274 | 1.181723 |
| 1 | 6 | C | 7.389954 | 1.284991 | 2.710141 |
| 2 | 6 | C | 7.916494 | 0.244997 | 5.333004 |
| 3 | 16 | S | 7.434185 | -3.216966 | 5.37084 |
| 4 | 6 | C | 9.01758 | -4.410523 | 2.534531 |
| 5 | 6 | C | 12.021461 | 1.114275 | 2.087074 |
| 6 | 6 | C | 11.899078 | -4.619517 | 2.30776 |
| 7 | 16 | S | 13.6132 | -1.842814 | 1.045924 |
| 8 | 6 | C | 13.416846 | 3.256017 | 0.596885 |
| 9 | 6 | C | 11.981187 | 3.265031 | -1.865895 |
| 10 | 6 | C | 9.343462 | 2.287907 | -1.433947 |
| 11 | 6 | C | 12.344602 | 1.521598 | 4.921104 |
| 12 | 7 | N | 10.300172 | 1.075564 | 6.380099 |
| 13 | 8 | O | 6.379351 | -1.101495 | -2.620232 |
| 14 | 6 | C | 4.139713 | 0.121455 | -2.774783 |
| 15 | 8 | O | 3.900536 | 2.203797 | -3.69707 |
| 16 | 6 | C | -13.641054 | -1.687216 | 1.117843 |
| 17 | 8 | O | 14.394256 | 2.247869 | 5.739011 |
| 18 | 6 | C | 10.453842 | 1.290581 | 9.117819 |
| 19 | 8 | O | 13.117695 | 5.598253 | 1.82873 |
| 20 | 6 | C | -11.61697 | -4.034562 | -2.457037 |
| 21 | 6 | C | -9.501876 | -4.595568 | -3.907623 |
| 22 | 6 | C | -7.160278 | -3.458333 | -3.387457 |
| 23 | 6 | C | -6.957119 | -1.763256 | -1.334553 |
| 24 | 6 | C | -9.077772 | -1.202083 | 0.10371 |
| 25 | 6 | C | -11.424243 | -2.318673 | -0.439558 |
| 26 | 6 | C | -0.404405 | -0.375725 | -1.782741 |
| 27 | 6 | C | -2.435689 | -1.691647 | -0.787789 |
| 28 | 6 | C | -2.070936 | -4.101883 | 0.320358 |
| 29 | 6 | C | 0.363591 | -5.147151 | 0.331435 |
| 30 | 6 | C | 2.400453 | -3.813679 | -0.680857 |
| 31 | 6 | C | 2.038892 | -1.412935 | -1.732155 |
| 32 | 8 | O | -4.132873 | -5.220858 | 1.295751 |
| 33 | 8 | O | -4.751104 | -0.516213 | -0.781109 |
| 34 | 6 | C | -3.937271 | -7.699905 | 2.306585 |
| 35 | 8 | O | -5.073716 | -3.918495 | -4.768711 |
| 36 | 1 | H | 7.879801 | 3.755054 | -1.55823 |
| 37 | 8 | O | -15.750792 | -2.553812 | 0.82214 |

| | | | | | |
|---|---|---|---|---|---|
| 38 | 6 | C | 13.105867 | 3.965373 | -4.022346 |
| 39 | 8 | O | 12.166382 | 4.01978 | -6.401253 |
| 40 | 6 | C | 10.108506 | 2.70163 | -7.221656 |
| 41 | 6 | C | 8.617272 | 1.047861 | -6.016376 |
| 42 | 6 | C | 8.679714 | 0.15974 | -3.328558 |
| 43 | 6 | C | 13.327335 | -5.950802 | 4.508921 |
| 44 | 6 | C | 13.755719 | -4.298889 | 6.841064 |
| 45 | 8 | O | 11.71781 | -8.035981 | 5.127143 |
| 46 | 6 | C | 15.864689 | -6.966118 | 3.543382 |
| 47 | 8 | O | 5.265 | 1.893441 | 2.05459 |
| 48 | 1 | H | 6.393565 | 0.938878 | 6.556919 |
| 49 | 1 | H | 8.239909 | -3.455251 | 0.867741 |
| 50 | 1 | H | 8.292816 | -6.35582 | 2.500042 |
| 51 | 1 | H | 12.122639 | -5.895766 | 0.676257 |
| 52 | 1 | H | 15.42916 | 2.78847 | 0.340364 |
| 53 | 1 | H | -13.252236 | -0.29678 | 2.663603 |
| 54 | 1 | H | 9.79884 | -0.47619 | 9.999947 |
| 55 | 1 | H | 9.257096 | 2.858649 | 9.785178 |
| 56 | 1 | H | 12.422037 | 1.642954 | 9.651812 |
| 57 | 1 | H | 14.111161 | 5.488917 | 3.372653 |
| 58 | 1 | H | -13.44011 | -4.908832 | -2.867574 |
| 59 | 1 | H | -9.625471 | -5.915943 | -5.494165 |
| 60 | 1 | H | -8.881686 | 0.118601 | 1.679367 |
| 61 | 1 | H | -0.723177 | 1.484837 | -2.609991 |
| 62 | 1 | H | 0.682592 | -7.004268 | 1.1621 |
| 63 | 1 | H | 4.279406 | -4.651971 | -0.636662 |
| 64 | 1 | H | -2.650309 | -7.754162 | 3.945168 |
| 65 | 1 | H | -5.847569 | -8.227529 | 2.920354 |
| 66 | 1 | H | -3.278885 | -9.054322 | 0.86581 |
| 67 | 1 | H | -5.457571 | -5.138851 | -6.088376 |
| 68 | 1 | H | 15.051366 | 4.660248 | -4.00724 |
| 69 | 1 | H | 9.79628 | 3.114562 | -9.219883 |
| 70 | 1 | H | 7.151716 | 0.199782 | -7.195913 |
| 71 | 1 | H | 10.103642 | -1.33261 | -3.084383 |
| 72 | 1 | H | 14.918367 | -2.636147 | 6.399335 |
| 73 | 1 | H | 14.765812 | -5.41036 | 8.285744 |
| 74 | 1 | H | 11.949184 | -3.680748 | 7.641629 |
| 75 | 1 | H | 12.515201 | -8.951892 | 6.49919 |
| 76 | 1 | H | 16.816166 | -8.036196 | 5.05641 |
| 77 | 1 | H | 15.571821 | -8.24017 | 1.923641 |
| 78 | 1 | H | 17.136517 | -5.427307 | 2.958969 |

| **Compd** 5000002_en_ | | Standard Orientation (A.U.) | | | |
|---|---|---|---|---|---|
| Center number | Atomic number | Atomic Type | X | Y | Z |
| 0 | 7 | N | 9.388998 | 1.340249 | 1.245154 |
| 1 | 6 | C | 7.33103 | 1.335923 | 2.773014 |
| 2 | 6 | C | 7.859258 | 0.377788 | 5.422887 |
| 3 | 16 | S | 7.431099 | -3.101698 | 5.518471 |
| 4 | 6 | C | 9.020944 | -4.331889 | 2.697078 |
| 5 | 6 | C | 11.969334 | 1.180841 | 2.170341 |
| 6 | 6 | C | 11.904012 | -4.520784 | 2.442947 |
| 7 | 16 | S | 13.602213 | -1.753775 | 1.126345 |
| 8 | 6 | C | 13.237917 | 3.351949 | 0.667238 |
| 9 | 6 | C | 11.931442 | 3.140285 | -1.856637 |
| 10 | 6 | C | 9.289295 | 2.179981 | -1.405032 |
| 11 | 6 | C | 12.321561 | 1.564948 | 5.012555 |
| 12 | 7 | N | 10.226141 | 1.252454 | 6.459084 |
| 13 | 8 | O | 6.314206 | -1.222101 | -2.503301 |
| 14 | 6 | C | 4.089367 | 0.017096 | -2.716357 |
| 15 | 8 | O | 3.87792 | 2.066587 | -3.716619 |
| 16 | 6 | C | -13.762212 | -1.631833 | 0.915253 |
| 17 | 8 | O | 14.408497 | 2.090528 | 5.847279 |
| 18 | 6 | C | 10.384625 | 1.44295 | 9.197048 |
| 19 | 8 | O | 12.540757 | 5.611606 | 1.926298 |
| 20 | 6 | C | -11.665958 | -4.153149 | -2.495737 |
| 21 | 6 | C | -9.524012 | -4.778675 | -3.878937 |
| 22 | 6 | C | -7.196914 | -3.603347 | -3.379301 |
| 23 | 6 | C | -7.037089 | -1.801646 | -1.415403 |
| 24 | 6 | C | -9.184215 | -1.175772 | -0.045217 |
| 25 | 6 | C | -11.515912 | -2.332223 | -0.568726 |
| 26 | 6 | C | -0.475497 | -0.428272 | -1.797534 |
| 27 | 6 | C | -2.529268 | -1.689889 | -0.777974 |
| 28 | 6 | C | -2.188541 | -4.03415 | 0.470326 |
| 29 | 6 | C | 0.245683 | -5.073667 | 0.594066 |
| 30 | 6 | C | 2.304557 | -3.796783 | -0.445624 |
| 31 | 6 | C | 1.965703 | -1.458474 | -1.636248 |
| 32 | 8 | O | -4.270426 | -5.100356 | 1.462406 |
| 33 | 8 | O | -4.843878 | -0.518686 | -0.892777 |
| 34 | 6 | C | -4.097316 | -7.52098 | 2.610001 |
| 35 | 8 | O | -5.084025 | -4.125021 | -4.697147 |
| 36 | 1 | H | 7.828345 | 3.64397 | -1.578556 |
| 37 | 8 | O | -15.862174 | -2.525481 | 0.630764 |
| 38 | 6 | C | 13.13526 | 3.681608 | -4.017008 |
| 39 | 8 | O | 12.28167 | 3.584584 | -6.426562 |

| | | | | | |
|---|---|---|---|---|---|
| 40 | 6 | C | 10.184288 | 2.309442 | -7.213389 |
| 41 | 6 | C | 8.615108 | 0.774895 | -5.950687 |
| 42 | 6 | C | 8.632936 | -0.006635 | -3.228735 |
| 43 | 6 | C | 13.3545 | -5.835017 | 4.640444 |
| 44 | 6 | C | 13.764684 | -4.184475 | 6.975297 |
| 45 | 8 | O | 11.771051 | -7.942001 | 5.257008 |
| 46 | 6 | C | 15.902225 | -6.819819 | 3.670657 |
| 47 | 8 | O | 5.20787 | 1.926636 | 2.091857 |
| 48 | 1 | H | 6.317468 | 1.066807 | 6.625418 |
| 49 | 1 | H | 8.21576 | -3.428254 | 1.015871 |
| 50 | 1 | H | 8.323913 | -6.287527 | 2.71424 |
| 51 | 1 | H | 12.114445 | -5.81378 | 0.822927 |
| 52 | 1 | H | 15.304197 | 3.128073 | 0.555631 |
| 53 | 1 | H | -13.406775 | -0.158901 | 2.390989 |
| 54 | 1 | H | 9.151265 | 2.97372 | 9.883721 |
| 55 | 1 | H | 12.346658 | 1.839475 | 9.723361 |
| 56 | 1 | H | 9.781723 | -0.346081 | 10.074432 |
| 57 | 1 | H | 12.898645 | 7.001762 | 0.786544 |
| 58 | 1 | H | -13.477857 | -5.057487 | -2.89034 |
| 59 | 1 | H | -9.614804 | -6.182237 | -5.394698 |
| 60 | 1 | H | -9.020868 | 0.226622 | 1.462051 |
| 61 | 1 | H | -0.775428 | 1.382442 | -2.734982 |
| 62 | 1 | H | 0.54567 | -6.88024 | 1.535622 |
| 63 | 1 | H | 4.182703 | -4.627019 | -0.310832 |
| 64 | 1 | H | -2.848002 | -7.484532 | 4.2779 |
| 65 | 1 | H | -6.020975 | -8.01702 | 3.208097 |
| 66 | 1 | H | -3.406383 | -8.950244 | 1.259724 |
| 67 | 1 | H | -5.43839 | -5.418448 | -5.953879 |
| 68 | 1 | H | 15.085805 | 4.361333 | -3.978102 |
| 69 | 1 | H | 9.912379 | 2.63723 | -9.233141 |
| 70 | 1 | H | 7.127971 | -0.068117 | -7.106243 |
| 71 | 1 | H | 10.042749 | -1.498868 | -2.911834 |
| 72 | 1 | H | 14.923351 | -2.518145 | 6.542204 |
| 73 | 1 | H | 14.764525 | -5.297037 | 8.426569 |
| 74 | 1 | H | 11.951601 | -3.566047 | 7.762294 |
| 75 | 1 | H | 12.578361 | -8.845175 | 6.631569 |
| 76 | 1 | H | 16.868544 | -7.879071 | 5.18199 |
| 77 | 1 | H | 15.622213 | -8.097246 | 2.051207 |
| 78 | 1 | H | 17.154694 | -5.265675 | 3.085526 |

| **Compd** 5000003_en_ | | Standard Orientation (A.U.) | | | |
|---|---|---|---|---|---|
| Center number | Atomic number | Atomic Type | X | Y | Z |
| 0 | 7 | N | 8.135638 | -0.090524 | 3.412086 |
| 1 | 6 | C | 6.591268 | -2.096936 | 3.803775 |
| 2 | 6 | C | 7.972603 | -4.482806 | 4.587781 |
| 3 | 16 | S | 8.90208 | -6.294099 | 1.738695 |
| 4 | 6 | C | 10.395009 | -4.025168 | -0.415428 |
| 5 | 6 | C | 10.726137 | 0.039998 | 4.312293 |
| 6 | 6 | C | 13.034692 | -2.90966 | 0.063455 |
| 7 | 16 | S | 13.061594 | 0.199582 | 1.692764 |
| 8 | 6 | C | 10.751155 | 2.682816 | 5.57278 |
| 9 | 6 | C | 9.109474 | 4.178827 | 3.785515 |
| 10 | 6 | C | 7.184472 | 2.40958 | 2.656195 |
| 11 | 6 | C | 11.522495 | -2.00481 | 6.182634 |
| 12 | 7 | N | 9.99383 | -4.04426 | 6.367141 |
| 13 | 8 | O | 5.601545 | 0.698709 | -1.365886 |
| 14 | 6 | C | 3.081624 | 0.574957 | -0.972994 |
| 15 | 8 | O | 1.887457 | 2.209717 | 0.098326 |
| 16 | 6 | C | -10.16112 | -0.243873 | 3.186948 |
| 17 | 8 | O | 13.496902 | -1.745829 | 7.370051 |
| 18 | 6 | C | 10.757068 | -6.18863 | 7.910276 |
| 19 | 8 | O | 9.564076 | 2.354886 | 7.952104 |
| 20 | 6 | C | -9.419499 | 1.144307 | -1.277787 |
| 21 | 6 | C | -8.220275 | 0.857756 | -3.596523 |
| 22 | 6 | C | -6.479999 | -1.111756 | -3.968177 |
| 23 | 6 | C | -5.955139 | -2.784007 | -1.955661 |
| 24 | 6 | C | -7.15062 | -2.489714 | 0.357582 |
| 25 | 6 | C | -8.902951 | -0.52754 | 0.720369 |
| 26 | 6 | C | -0.640725 | -2.116769 | -1.785349 |
| 27 | 6 | C | -1.804755 | -4.281649 | -2.712594 |
| 28 | 6 | C | -0.367493 | -6.133537 | -3.999116 |
| 29 | 6 | C | 2.249485 | -5.767892 | -4.22423 |
| 30 | 6 | C | 3.421999 | -3.619139 | -3.264487 |
| 31 | 6 | C | 1.969941 | -1.760131 | -2.05644 |
| 32 | 8 | O | -1.329332 | -8.288962 | -4.96525 |
| 33 | 8 | O | -4.325719 | -4.774002 | -2.327804 |
| 34 | 6 | C | -3.758361 | -8.307678 | -6.150664 |
| 35 | 8 | O | -5.265911 | -1.50748 | -6.165536 |
| 36 | 1 | H | 5.272351 | 2.650156 | 3.426854 |
| 37 | 8 | O | -11.695351 | 1.388985 | 3.702017 |
| 38 | 6 | C | 9.53696 | 6.633919 | 3.337081 |
| 39 | 8 | O | 8.295968 | 8.243333 | 1.784068 |

| | | | | | |
|---|---|---|---|---|---|
| 40 | 6 | C | 6.733973 | 7.547617 | -0.145983 |
| 41 | 6 | C | 6.175939 | 5.259242 | -1.075403 |
| 42 | 6 | C | 7.061075 | 2.69999 | -0.248954 |
| 43 | 6 | C | 15.175457 | -4.719332 | 0.990339 |
| 44 | 6 | C | 17.760665 | -3.455434 | 0.633166 |
| 45 | 8 | O | 14.864427 | -5.414071 | 3.565552 |
| 46 | 6 | C | 15.09853 | -7.182717 | -0.525796 |
| 47 | 8 | O | 4.309539 | -2.063745 | 3.466818 |
| 48 | 1 | H | 6.568624 | -5.724315 | 5.474442 |
| 49 | 1 | H | 9.045597 | -2.509787 | -0.842994 |
| 50 | 1 | H | 10.513087 | -5.17883 | -2.14198 |
| 51 | 1 | H | 13.633527 | -2.265234 | -1.824912 |
| 52 | 1 | H | 12.678537 | 3.443206 | 5.764326 |
| 53 | 1 | H | -9.593193 | -1.687087 | 4.624262 |
| 54 | 1 | H | 12.238021 | -5.57408 | 9.218432 |
| 55 | 1 | H | 11.509619 | -7.701224 | 6.695377 |
| 56 | 1 | H | 9.118601 | -6.898381 | 8.975309 |
| 57 | 1 | H | 9.165077 | 4.022964 | 8.598604 |
| 58 | 1 | H | -10.778287 | 2.669151 | -0.983328 |
| 59 | 1 | H | -8.621445 | 2.145673 | -5.162428 |
| 60 | 1 | H | -6.707288 | -3.806392 | 1.885429 |
| 61 | 1 | H | -1.753658 | -0.692067 | -0.797017 |
| 62 | 1 | H | 3.336221 | -7.22438 | -5.202263 |
| 63 | 1 | H | 5.45577 | -3.37641 | -3.469324 |
| 64 | 1 | H | -5.269743 | -8.701877 | -4.779664 |
| 65 | 1 | H | -4.153816 | -6.501238 | -7.101674 |
| 66 | 1 | H | -3.698995 | -9.831174 | -7.563478 |
| 67 | 1 | H | -5.759337 | -0.233115 | -7.396193 |
| 68 | 1 | H | 11.045351 | 7.628265 | 4.339236 |
| 69 | 1 | H | 5.941974 | 9.242974 | -1.017143 |
| 70 | 1 | H | 4.920329 | 5.282597 | -2.712641 |
| 71 | 1 | H | 8.97073 | 2.319121 | -0.969794 |
| 72 | 1 | H | 19.254061 | -4.75605 | 1.269225 |
| 73 | 1 | H | 17.897058 | -1.709637 | 1.756213 |
| 74 | 1 | H | 18.107184 | -2.974009 | -1.36174 |
| 75 | 1 | H | 15.210729 | -3.962979 | 4.635884 |
| 76 | 1 | H | 15.182362 | -6.810138 | -2.570099 |
| 77 | 1 | H | 16.734286 | -8.357671 | -0.005004 |
| 78 | 1 | H | 13.379548 | -8.267667 | -0.106701 |

| **Compd** 5000005_en_ | | Standard Orientation (A.U.) | | | |
|---|---|---|---|---|---|
| Center number | Atomic number | Atomic Type | X | Y | Z |
| 0 | 7 | N | 9.426048 | 1.497806 | 1.118055 |
| 1 | 6 | C | 7.344022 | 1.472278 | 2.617195 |
| 2 | 6 | C | 7.83118 | 0.524715 | 5.282569 |
| 3 | 16 | S | 7.340142 | -2.93188 | 5.436045 |
| 4 | 6 | C | 8.996538 | -4.237382 | 2.690394 |
| 5 | 6 | C | 11.984998 | 1.289035 | 2.070518 |
| 6 | 6 | C | 11.883943 | -4.440446 | 2.53269 |
| 7 | 16 | S | 13.596837 | -1.702815 | 1.167766 |
| 8 | 6 | C | 13.39857 | 3.377502 | 0.523052 |
| 9 | 6 | C | 11.999908 | 3.29085 | -1.95968 |
| 10 | 6 | C | 9.358192 | 2.322364 | -1.531625 |
| 11 | 6 | C | 12.262316 | 1.797948 | 4.892578 |
| 12 | 7 | N | 10.199375 | 1.389398 | 6.33604 |
| 13 | 8 | O | 6.438393 | -1.136939 | -2.625177 |
| 14 | 6 | C | 4.186756 | 0.052096 | -2.852982 |
| 15 | 8 | O | 3.93249 | 2.090795 | -3.864029 |
| 16 | 6 | C | -13.506997 | -1.639557 | 1.454524 |
| 17 | 8 | O | 14.296198 | 2.561478 | 5.716518 |
| 18 | 6 | C | 10.311287 | 1.700778 | 9.066507 |
| 19 | 8 | O | 13.077791 | 5.76341 | 1.661891 |
| 20 | 6 | C | -11.592175 | -4.130456 | -2.083075 |
| 21 | 6 | C | -9.523851 | -4.748549 | -3.577301 |
| 22 | 6 | C | -7.170265 | -3.586022 | -3.182584 |
| 23 | 6 | C | -6.906433 | -1.807223 | -1.209151 |
| 24 | 6 | C | -8.980827 | -1.187158 | 0.270998 |
| 25 | 6 | C | -11.340237 | -2.330019 | -0.147603 |
| 26 | 6 | C | -0.35071 | -0.432526 | -1.823794 |
| 27 | 6 | C | -2.37138 | -1.720375 | -0.770876 |
| 28 | 6 | C | -1.990405 | -4.095029 | 0.407574 |
| 29 | 6 | C | 0.445798 | -5.136087 | 0.424663 |
| 30 | 6 | C | 2.471403 | -3.82953 | -0.643064 |
| 31 | 6 | C | 2.09568 | -1.461149 | -1.760561 |
| 32 | 8 | O | -4.037158 | -5.187022 | 1.443792 |
| 33 | 8 | O | -4.684295 | -0.540794 | -0.776972 |
| 34 | 6 | C | -3.824261 | -7.634452 | 2.52577 |
| 35 | 8 | O | -5.126731 | -4.099999 | -4.608486 |
| 36 | 1 | H | 7.891802 | 3.77799 | -1.735133 |
| 37 | 8 | O | -15.62103 | -2.525655 | 1.267209 |
| 38 | 6 | C | 13.157165 | 3.906088 | -4.124966 |
| 39 | 8 | O | 12.254753 | 3.864962 | -6.518531 |

| | | | | | |
|---|---|---|---|---|---|
| 40 | 6 | C | 10.20759 | 2.517223 | -7.317573 |
| 41 | 6 | C | 8.701684 | 0.906772 | -6.072461 |
| 42 | 6 | C | 8.735104 | 0.119009 | -3.353005 |
| 43 | 6 | C | 13.283522 | -5.686585 | 4.818392 |
| 44 | 6 | C | 13.610289 | -3.957623 | 7.099646 |
| 45 | 8 | O | 11.768954 | -7.763384 | 5.655164 |
| 46 | 6 | C | 15.863317 | -6.673933 | 3.94833 |
| 47 | 8 | O | 5.229356 | 2.058199 | 1.909952 |
| 48 | 1 | H | 6.290649 | 1.261379 | 6.458942 |
| 49 | 1 | H | 8.253335 | -3.355893 | 0.96818 |
| 50 | 1 | H | 8.265707 | -6.181115 | 2.718834 |
| 51 | 1 | H | 12.146863 | -5.774529 | 0.95091 |
| 52 | 1 | H | 15.415055 | 2.904511 | 0.313513 |
| 53 | 1 | H | -13.073849 | -0.182776 | 2.925479 |
| 54 | 1 | H | 12.273633 | 2.055228 | 9.62011 |
| 55 | 1 | H | 9.625418 | -0.027159 | 9.999675 |
| 56 | 1 | H | 9.119369 | 3.302593 | 9.658385 |
| 57 | 1 | H | 14.042579 | 5.711208 | 3.226874 |
| 58 | 1 | H | -13.424499 | -5.025473 | -2.396226 |
| 59 | 1 | H | -9.696109 | -6.13723 | -5.099364 |
| 60 | 1 | H | -8.736879 | 0.198098 | 1.783229 |
| 61 | 1 | H | -0.679678 | 1.403175 | -2.701025 |
| 62 | 1 | H | 0.775134 | -6.967182 | 1.307188 |
| 63 | 1 | H | 4.352245 | -4.662642 | -0.589978 |
| 64 | 1 | H | -5.72587 | -8.146237 | 3.178582 |
| 65 | 1 | H | -3.181084 | -9.030189 | 1.118032 |
| 66 | 1 | H | -2.517136 | -7.637288 | 4.149158 |
| 67 | 1 | H | -5.545734 | -5.381354 | -5.857811 |
| 68 | 1 | H | 15.101607 | 4.603709 | -4.107615 |
| 69 | 1 | H | 9.921499 | 2.857215 | -9.333327 |
| 70 | 1 | H | 7.250581 | 0.015605 | -7.237632 |
| 71 | 1 | H | 10.168482 | -1.349926 | -3.033984 |
| 72 | 1 | H | 14.715919 | -2.271989 | 6.608569 |
| 73 | 1 | H | 14.625329 | -4.991918 | 8.592526 |
| 74 | 1 | H | 11.768065 | -3.377175 | 7.845311 |
| 75 | 1 | H | 11.859025 | -9.071666 | 4.373219 |
| 76 | 1 | H | 16.796084 | -7.66766 | 5.519854 |
| 77 | 1 | H | 15.649079 | -8.008565 | 2.360241 |
| 78 | 1 | H | 17.104028 | -5.126287 | 3.324735 |

| **Compd** 5000006_en_ | | Standard Orientation (A.U.) | | | |
|---|---|---|---|---|---|
| Center number | Atomic number | Atomic Type | X | Y | Z |
| 0 | 7 | N | 7.154968 | 1.147034 | 2.836018 |
| 1 | 6 | C | 4.766328 | 0.236803 | 2.645569 |
| 2 | 6 | C | 4.437599 | -2.402435 | 3.704591 |
| 3 | 16 | S | 5.131466 | -4.766668 | 1.20948 |
| 4 | 6 | C | 8.042835 | -3.692043 | -0.33515 |
| 5 | 6 | C | 9.032267 | 0.113008 | 4.553701 |
| 6 | 6 | C | 10.668789 | -3.931373 | 0.874208 |
| 7 | 16 | S | 11.789497 | -1.256791 | 2.858608 |
| 8 | 6 | C | 10.010057 | 2.554133 | 5.854101 |
| 9 | 6 | C | 9.941772 | 4.43223 | 3.712787 |
| 10 | 6 | C | 7.863719 | 3.67253 | 1.921033 |
| 11 | 6 | C | 8.050048 | -1.756816 | 6.531402 |
| 12 | 7 | N | 5.76413 | -2.818262 | 6.048511 |
| 13 | 8 | O | 7.212875 | 2.311783 | -2.493673 |
| 14 | 6 | C | 4.858595 | 3.173695 | -3.008255 |
| 15 | 8 | O | 4.120531 | 5.267517 | -2.465979 |
| 16 | 6 | C | -1.902612 | -6.238957 | 3.185766 |
| 17 | 8 | O | 9.331704 | -2.228645 | 8.391456 |
| 18 | 6 | C | 4.715643 | -4.716388 | 7.73919 |
| 19 | 8 | O | 8.184092 | 3.164539 | 7.719675 |
| 20 | 6 | C | -6.225746 | -4.766333 | 1.953478 |
| 21 | 6 | C | -7.799907 | -3.427296 | 0.328238 |
| 22 | 6 | C | -6.798825 | -2.114553 | -1.746793 |
| 23 | 6 | C | -4.159336 | -2.150569 | -2.179353 |
| 24 | 6 | C | -2.592279 | -3.499327 | -0.560635 |
| 25 | 6 | C | -3.61128 | -4.818484 | 1.513911 |
| 26 | 6 | C | 0.703715 | 1.217086 | -3.865985 |
| 27 | 6 | C | -0.773236 | -0.772184 | -4.698844 |
| 28 | 6 | C | 0.308307 | -2.843042 | -5.999036 |
| 29 | 6 | C | 2.925194 | -2.849518 | -6.425309 |
| 30 | 6 | C | 4.418301 | -0.87715 | -5.512155 |
| 31 | 6 | C | 3.329643 | 1.165725 | -4.231059 |
| 32 | 8 | O | -1.289725 | -4.69281 | -6.679063 |
| 33 | 8 | O | -3.329682 | -0.777382 | -4.210676 |
| 34 | 6 | C | -0.298728 | -6.914022 | -7.820276 |
| 35 | 8 | O | -8.231747 | -0.790518 | -3.376905 |
| 36 | 1 | H | 6.184879 | 4.88926 | 2.000749 |
| 37 | 8 | O | -2.53246 | -7.351233 | 5.098171 |
| 38 | 6 | C | 11.605179 | 6.334473 | 3.553883 |
| 39 | 8 | O | 11.801876 | 8.17618 | 1.786369 |

| | | | | | |
|---|---|---|---|---|---|
| 40 | 6 | C | 10.711007 | 8.128521 | -0.5505 |
| 41 | 6 | C | 9.484301 | 6.266691 | -1.751243 |
| 42 | 6 | C | 8.878211 | 3.657799 | -0.831623 |
| 43 | 6 | C | 11.426775 | -6.587462 | 1.929356 |
| 44 | 6 | C | 10.277661 | -7.223166 | 4.492803 |
| 45 | 8 | O | 10.482471 | -8.437837 | 0.199098 |
| 46 | 6 | C | 14.312963 | -6.789331 | 2.058919 |
| 47 | 8 | O | 3.031793 | 1.35781 | 1.618498 |
| 48 | 1 | H | 2.407744 | -2.658145 | 4.039186 |
| 49 | 1 | H | 7.759431 | -1.770874 | -1.053963 |
| 50 | 1 | H | 8.046061 | -4.943985 | -1.991507 |
| 51 | 1 | H | 11.927377 | -3.632108 | -0.761492 |
| 52 | 1 | H | 11.904084 | 2.297856 | 6.678513 |
| 53 | 1 | H | 0.113334 | -6.245388 | 2.55476 |
| 54 | 1 | H | 6.011935 | -4.976575 | 9.331373 |
| 55 | 1 | H | 4.481656 | -6.5251 | 6.734563 |
| 56 | 1 | H | 2.851899 | -4.1028 | 8.434929 |
| 57 | 1 | H | 8.378842 | 4.945551 | 8.10697 |
| 58 | 1 | H | -7.000649 | -5.789802 | 3.568582 |
| 59 | 1 | H | -9.843565 | -3.374715 | 0.638406 |
| 60 | 1 | H | -0.553276 | -3.52272 | -0.870106 |
| 61 | 1 | H | -0.171007 | 2.75637 | -2.813468 |
| 62 | 1 | H | 3.812768 | -4.414723 | -7.426066 |
| 63 | 1 | H | 6.458517 | -0.945702 | -5.790234 |
| 64 | 1 | H | -1.916184 | -8.170615 | -8.149599 |
| 65 | 1 | H | 0.620123 | -6.488222 | -9.641895 |
| 66 | 1 | H | 1.066289 | -7.86319 | -6.563025 |
| 67 | 1 | H | -9.995568 | -0.901218 | -2.871201 |
| 68 | 1 | H | 13.032723 | 6.600409 | 5.0233 |
| 69 | 1 | H | 11.042872 | 9.935753 | -1.490508 |
| 70 | 1 | H | 8.869947 | 6.72231 | -3.666505 |
| 71 | 1 | H | 10.573445 | 2.459651 | -0.842466 |
| 72 | 1 | H | 8.209237 | -7.215177 | 4.388927 |
| 73 | 1 | H | 10.889364 | -5.872783 | 5.942238 |
| 74 | 1 | H | 10.897446 | -9.127037 | 5.059192 |
| 75 | 1 | H | 11.472097 | -8.319661 | -1.340759 |
| 76 | 1 | H | 15.117255 | -5.416305 | 3.397083 |
| 77 | 1 | H | 14.852195 | -8.70045 | 2.679178 |
| 78 | 1 | H | 15.163255 | -6.439941 | 0.187162 |

| **Compd** 5000007_en_ | | Standard Orientation (A.U.) | | | |
|---|---|---|---|---|---|
| Center number | Atomic number | Atomic Type | X | Y | Z |
| 0 | 7 | N | 7.223072 | 1.02635 | 2.78653 |
| 1 | 6 | C | 4.796658 | 0.2102 | 2.661022 |
| 2 | 6 | C | 4.38582 | -2.395719 | 3.77333 |
| 3 | 16 | S | 4.939442 | -4.82715 | 1.306776 |
| 4 | 6 | C | 7.870702 | -3.905777 | -0.297571 |
| 5 | 6 | C | 9.089403 | -0.044609 | 4.493687 |
| 6 | 6 | C | 10.502971 | -4.227246 | 0.878679 |
| 7 | 16 | S | 11.76113 | -1.555774 | 2.783361 |
| 8 | 6 | C | 10.182007 | 2.383268 | 5.725547 |
| 9 | 6 | C | 10.149848 | 4.217825 | 3.545744 |
| 10 | 6 | C | 8.011757 | 3.503346 | 1.806641 |
| 11 | 6 | C | 8.071385 | -1.836545 | 6.524346 |
| 12 | 7 | N | 5.739746 | -2.820894 | 6.099981 |
| 13 | 8 | O | 7.230871 | 2.075021 | -2.565734 |
| 14 | 6 | C | 4.900825 | 3.012206 | -3.060024 |
| 15 | 8 | O | 4.250013 | 5.143861 | -2.555033 |
| 16 | 6 | C | -2.076295 | -6.03268 | 3.464768 |
| 17 | 8 | O | 9.366515 | -2.321241 | 8.371861 |
| 18 | 6 | C | 4.649327 | -4.641022 | 7.848894 |
| 19 | 8 | O | 8.410889 | 3.102171 | 7.605569 |
| 20 | 6 | C | -6.372419 | -4.473717 | 2.247003 |
| 21 | 6 | C | -7.928586 | -3.124982 | 0.612774 |
| 22 | 6 | C | -6.916366 | -1.878116 | -1.497113 |
| 23 | 6 | C | -4.283697 | -1.992 | -1.956879 |
| 24 | 6 | C | -2.734853 | -3.351271 | -0.329415 |
| 25 | 6 | C | -3.765141 | -4.602766 | 1.781233 |
| 26 | 6 | C | 0.663902 | 1.182182 | -3.794905 |
| 27 | 6 | C | -0.894967 | -0.773499 | -4.555107 |
| 28 | 6 | C | 0.090544 | -2.907945 | -5.828756 |
| 29 | 6 | C | 2.696399 | -3.01156 | -6.306487 |
| 30 | 6 | C | 4.272435 | -1.071637 | -5.46622 |
| 31 | 6 | C | 3.279002 | 1.033518 | -4.208985 |
| 32 | 8 | O | -1.582154 | -4.717021 | -6.435392 |
| 33 | 8 | O | -3.440728 | -0.679326 | -4.022341 |
| 34 | 6 | C | -0.689854 | -6.992601 | -7.550973 |
| 35 | 8 | O | -8.332936 | -0.548081 | -3.136552 |
| 36 | 1 | H | 6.381709 | 4.784354 | 1.890609 |
| 37 | 8 | O | -2.714826 | -7.090668 | 5.404963 |
| 38 | 6 | C | 11.884508 | 6.047861 | 3.316275 |
| 39 | 8 | O | 12.121476 | 7.842838 | 1.50587 |

| | | | | | |
|---|---|---|---|---|---|
| 40 | 6 | C | 10.984623 | 7.791237 | -0.808926 |
| 41 | 6 | C | 9.663177 | 5.955326 | -1.947556 |
| 42 | 6 | C | 8.974769 | 3.39159 | -0.961833 |
| 43 | 6 | C | 11.17211 | -6.889352 | 1.977785 |
| 44 | 6 | C | 10.020732 | -7.439636 | 4.559609 |
| 45 | 8 | O | 10.147559 | -8.734023 | 0.286906 |
| 46 | 6 | C | 14.049893 | -7.194231 | 2.091364 |
| 47 | 8 | O | 3.087225 | 1.382422 | 1.648954 |
| 48 | 1 | H | 2.354674 | -2.565789 | 4.150079 |
| 49 | 1 | H | 7.655589 | -1.989936 | -1.052676 |
| 50 | 1 | H | 7.799319 | -5.191123 | -1.926702 |
| 51 | 1 | H | 11.74691 | -4.01515 | -0.781385 |
| 52 | 1 | H | 12.078345 | 2.072143 | 6.525614 |
| 53 | 1 | H | -0.067227 | -6.10138 | 2.816435 |
| 54 | 1 | H | 4.370551 | -6.475281 | 6.903697 |
| 55 | 1 | H | 2.801884 | -3.960923 | 8.525611 |
| 56 | 1 | H | 5.941271 | -4.882319 | 9.447603 |
| 57 | 1 | H | 8.684561 | 4.88014 | 7.957083 |
| 58 | 1 | H | -7.155907 | -5.44548 | 3.889671 |
| 59 | 1 | H | -9.966752 | -3.012936 | 0.94217 |
| 60 | 1 | H | -0.701049 | -3.436524 | -0.661941 |
| 61 | 1 | H | -0.138243 | 2.772355 | -2.760406 |
| 62 | 1 | H | 3.511682 | -4.626046 | -7.290552 |
| 63 | 1 | H | 6.303333 | -1.214688 | -5.782201 |
| 64 | 1 | H | 0.207802 | -6.63333 | -9.397334 |
| 65 | 1 | H | 0.6658 | -7.964236 | -6.300683 |
| 66 | 1 | H | -2.355175 | -8.198792 | -7.825577 |
| 67 | 1 | H | -10.093439 | -0.605515 | -2.610664 |
| 68 | 1 | H | 13.348207 | 6.287678 | 4.75443 |
| 69 | 1 | H | 11.3683 | 9.564777 | -1.792434 |
| 70 | 1 | H | 9.029066 | 6.396853 | -3.859662 |
| 71 | 1 | H | 10.623318 | 2.129859 | -0.974879 |
| 72 | 1 | H | 7.952703 | -7.377992 | 4.465542 |
| 73 | 1 | H | 10.677811 | -6.078057 | 5.978474 |
| 74 | 1 | H | 10.59172 | -9.347647 | 5.162567 |
| 75 | 1 | H | 11.127715 | -8.67815 | -1.262558 |
| 76 | 1 | H | 14.912184 | -5.834044 | 3.406271 |
| 77 | 1 | H | 14.522364 | -9.115372 | 2.734494 |
| 78 | 1 | H | 14.900855 | -6.900781 | 0.210296 |

| **Compd** 5000009_en_ | | Standard Orientation (A.U.) | | | |
|---|---|---|---|---|---|
| Center number | Atomic number | Atomic Type | X | Y | Z |
| 0 | 7 | N | 8.68867 | 1.349565 | 2.556127 |
| 1 | 6 | C | 7.007868 | 0.102342 | 4.038549 |
| 2 | 6 | C | 8.21613 | -1.687081 | 5.933714 |
| 3 | 16 | S | 8.506336 | -4.87398 | 4.500349 |
| 4 | 6 | C | 9.782808 | -4.343052 | 1.325356 |
| 5 | 6 | C | 11.37255 | 1.492697 | 3.087631 |
| 6 | 6 | C | 12.565222 | -3.668675 | 0.892799 |
| 7 | 16 | S | 13.363758 | -0.261625 | 0.77852 |
| 8 | 6 | C | 11.962809 | 4.342874 | 2.554229 |
| 9 | 6 | C | 10.140087 | 4.918307 | 0.440123 |
| 10 | 6 | C | 7.908593 | 3.159441 | 0.598556 |
| 11 | 6 | C | 12.09074 | 0.835072 | 5.799322 |
| 12 | 7 | N | 10.49788 | -0.709305 | 7.063658 |
| 13 | 8 | O | 5.612747 | -0.167658 | -1.713596 |
| 14 | 6 | C | 3.166584 | 0.336005 | -1.180489 |
| 15 | 8 | O | 2.294343 | 2.451073 | -1.104896 |
| 16 | 6 | C | -7.842025 | -1.646936 | -7.628157 |
| 17 | 8 | O | 14.026152 | 1.732656 | 6.715185 |
| 18 | 6 | C | 11.069044 | -1.519221 | 9.630638 |
| 19 | 8 | O | 11.379712 | 5.833887 | 4.682301 |
| 20 | 6 | C | -10.690236 | -1.554062 | -3.855015 |
| 21 | 6 | C | -11.085619 | -1.843791 | -1.271967 |
| 22 | 6 | C | -9.068149 | -2.514322 | 0.304817 |
| 23 | 6 | C | -6.64121 | -2.879166 | -0.745952 |
| 24 | 6 | C | -6.241745 | -2.612236 | -3.320566 |
| 25 | 6 | C | -8.278208 | -1.937351 | -4.895734 |
| 26 | 6 | C | -0.877385 | -1.772138 | -0.181177 |
| 27 | 6 | C | -2.359875 | -3.890959 | 0.290793 |
| 28 | 6 | C | -1.306145 | -6.342134 | 0.187527 |
| 29 | 6 | C | 1.289732 | -6.550738 | -0.368359 |
| 30 | 6 | C | 2.767118 | -4.437043 | -0.837383 |
| 31 | 6 | C | 1.690405 | -2.010132 | -0.755915 |
| 32 | 8 | O | -2.521301 | -8.523593 | 0.650289 |
| 33 | 8 | O | -4.851626 | -3.566535 | 1.005221 |
| 34 | 6 | C | -5.194472 | -8.786204 | 0.388074 |
| 35 | 8 | O | -9.436109 | -2.826206 | 2.799128 |
| 36 | 1 | H | 6.157922 | 4.08726 | 1.21893 |
| 37 | 8 | O | -9.450913 | -1.07313 | -9.167921 |
| 38 | 6 | C | 10.670684 | 6.695972 | -1.281523 |
| 39 | 8 | O | 9.327983 | 7.422464 | -3.334163 |

| | | | | | |
|---|---|---|---|---|---|
| 40 | 6 | C | 7.498261 | 6.022019 | -4.489315 |
| 41 | 6 | C | 6.656453 | 3.683207 | -4.007176 |
| 42 | 6 | C | 7.405561 | 1.860645 | -1.97345 |
| 43 | 6 | C | 14.583323 | -5.324655 | 2.302085 |
| 44 | 6 | C | 15.214066 | -4.440718 | 4.978374 |
| 45 | 8 | O | 13.652478 | -7.861453 | 2.36544 |
| 46 | 6 | C | 16.985869 | -5.409201 | 0.700979 |
| 47 | 8 | O | 4.718242 | 0.28817 | 3.845676 |
| 48 | 1 | H | 6.818943 | -1.988026 | 7.435721 |
| 49 | 1 | H | 8.54749 | -3.036973 | 0.295302 |
| 50 | 1 | H | 9.488124 | -6.218877 | 0.482302 |
| 51 | 1 | H | 12.81799 | -4.11106 | -1.126908 |
| 52 | 1 | H | 13.948089 | 4.589729 | 1.978252 |
| 53 | 1 | H | -5.846505 | -2.001133 | -8.233338 |
| 54 | 1 | H | 12.983798 | -0.902992 | 10.117225 |
| 55 | 1 | H | 10.952558 | -3.592468 | 9.755828 |
| 56 | 1 | H | 9.706231 | -0.688072 | 10.967887 |
| 57 | 1 | H | 12.632271 | 5.403477 | 5.958437 |
| 58 | 1 | H | -12.245384 | -1.030579 | -5.105774 |
| 59 | 1 | H | -12.947504 | -1.56472 | -0.426392 |
| 60 | 1 | H | -4.369897 | -2.923954 | -4.129906 |
| 61 | 1 | H | -1.736925 | 0.098634 | -0.071296 |
| 62 | 1 | H | 2.102077 | -8.446558 | -0.428991 |
| 63 | 1 | H | 4.764941 | -4.667408 | -1.275229 |
| 64 | 1 | H | -5.856237 | -8.001046 | -1.4222 |
| 65 | 1 | H | -5.560415 | -10.829767 | 0.405953 |
| 66 | 1 | H | -6.222723 | -7.89699 | 1.958183 |
| 67 | 1 | H | -7.819927 | -3.22803 | 3.58469 |
| 68 | 1 | H | 12.383423 | 7.838044 | -1.108007 |
| 69 | 1 | H | 6.742645 | 7.086823 | -6.087917 |
| 70 | 1 | H | 5.225546 | 2.999965 | -5.327739 |
| 71 | 1 | H | 9.146067 | 0.855757 | -2.497199 |
| 72 | 1 | H | 13.541617 | -4.50248 | 6.201153 |
| 73 | 1 | H | 15.97141 | -2.509362 | 4.989342 |
| 74 | 1 | H | 16.649608 | -5.711839 | 5.78623 |
| 75 | 1 | H | 12.518228 | -7.99692 | 3.79841 |
| 76 | 1 | H | 16.582014 | -6.24381 | -1.162465 |
| 77 | 1 | H | 17.758187 | -3.499365 | 0.416284 |
| 78 | 1 | H | 18.431251 | -6.569582 | 1.64444 |

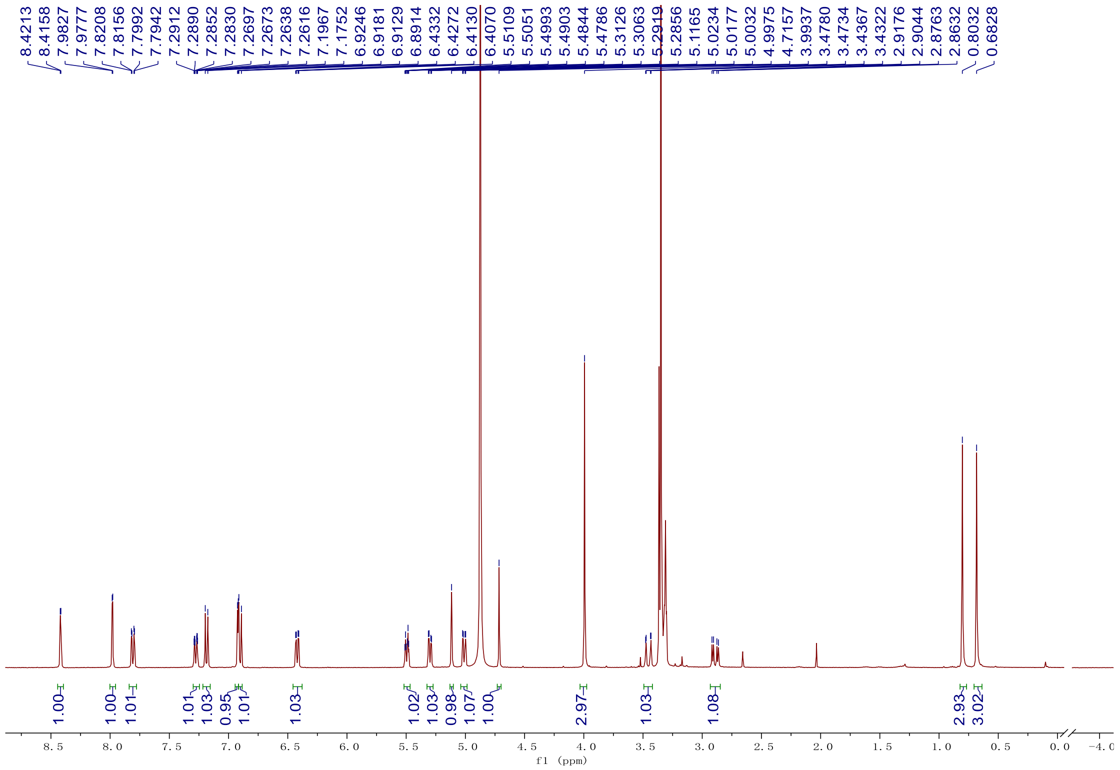

**Figure S3.** $^{1}$H NMR (400 MHz) spectrum of **1** recorded in $CD_3OD$

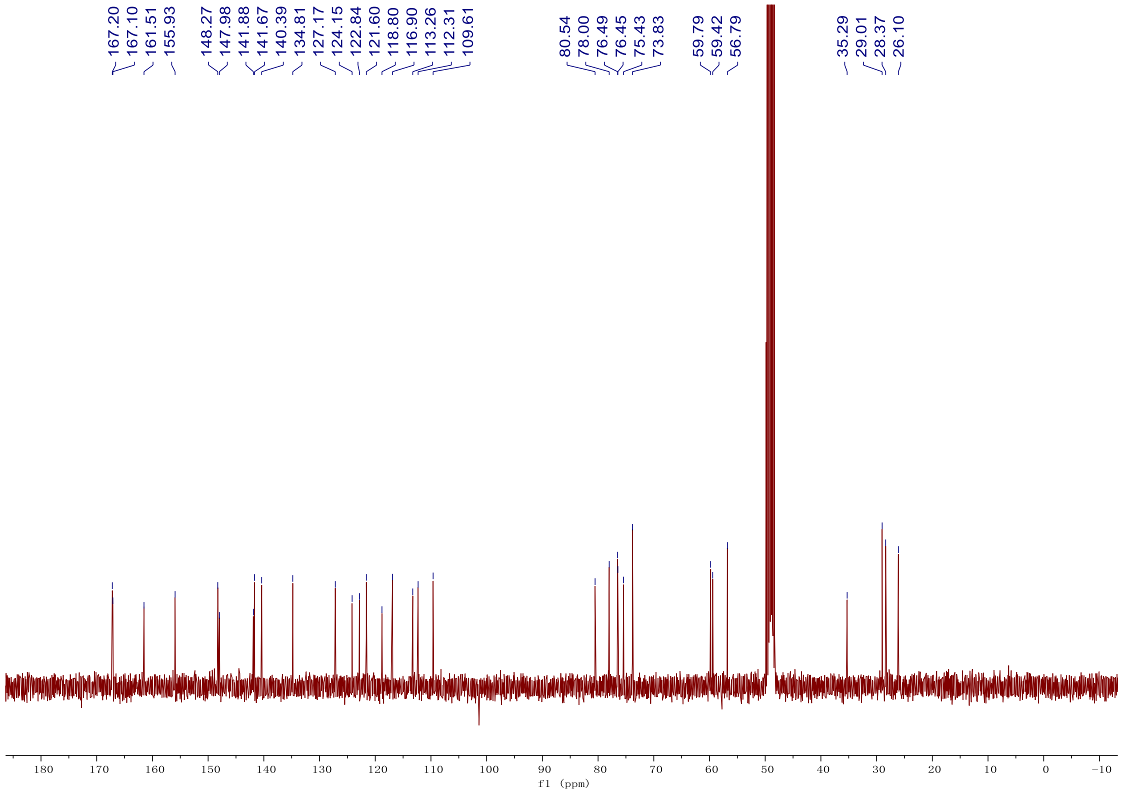

**Figure S4**. $^{13}$C NMR (100 MHz) spectrum of **1** recorded in $CD_3OD$

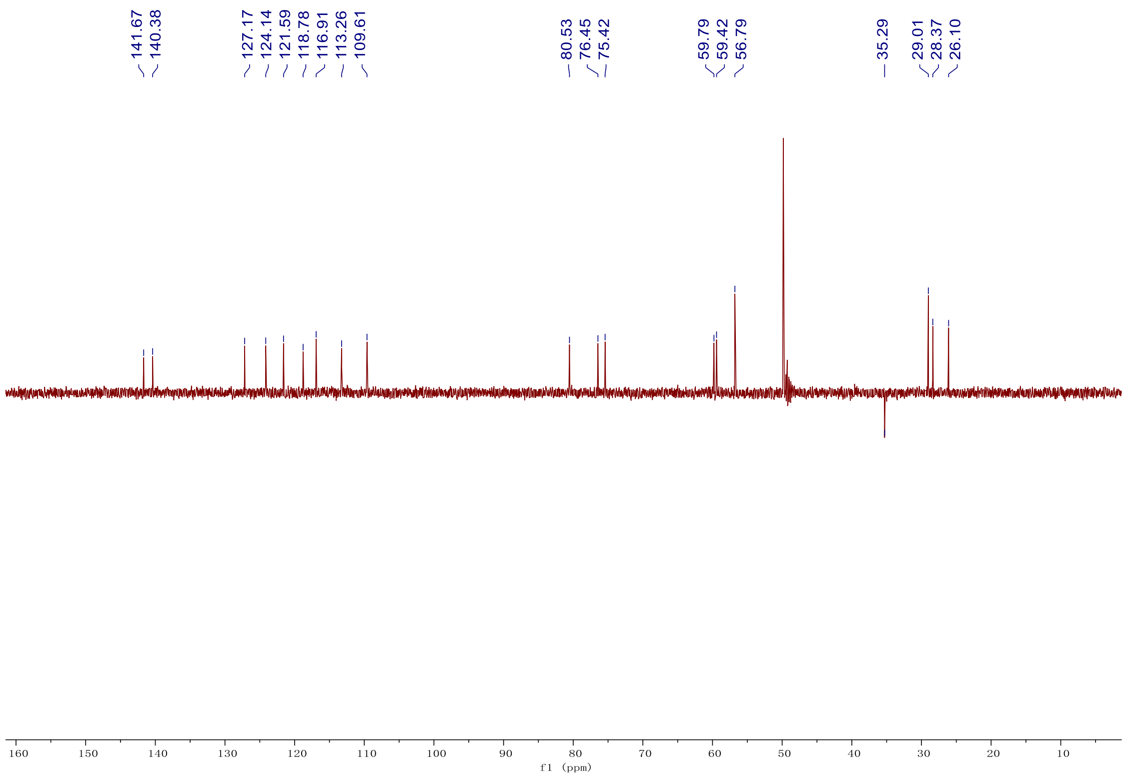


**Figure S5.** DEPT NMR spectrum of **1** recorded in $CD_3OD$

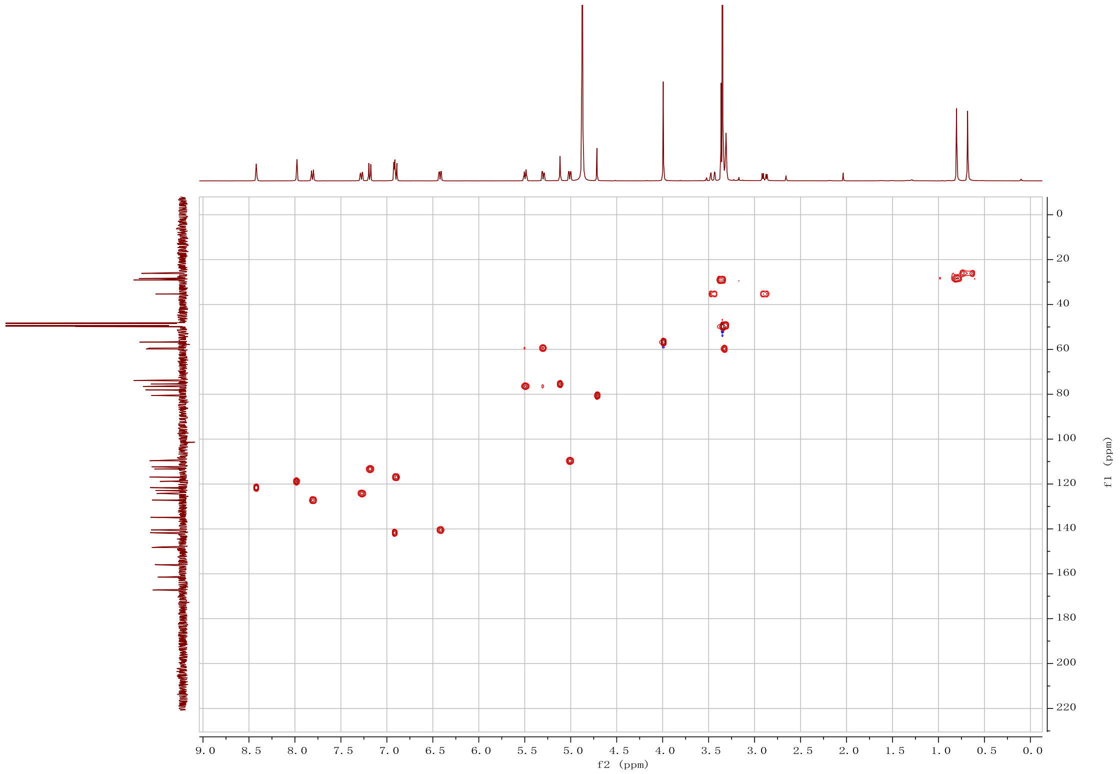


**Figure S6.** HSQC spectrum of **1** recorded in $CD_3OD$

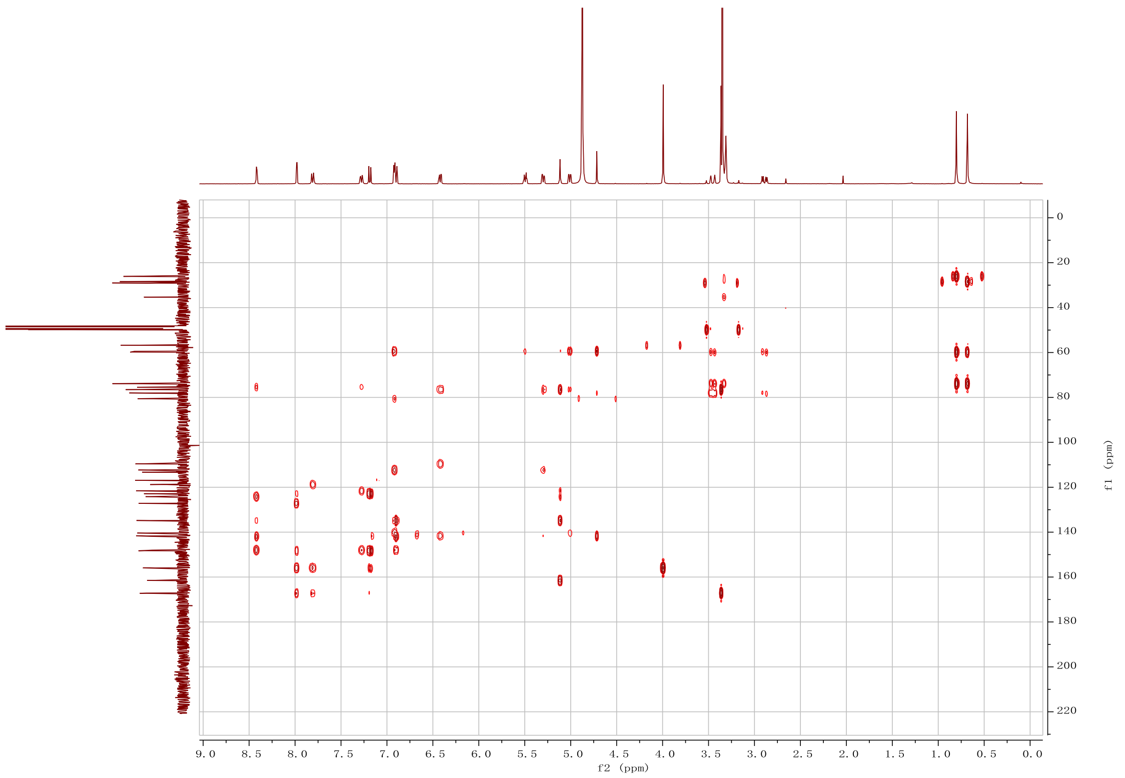


**Figure S7.** HMBC spectrum of **1** recorded in $CD_3OD$

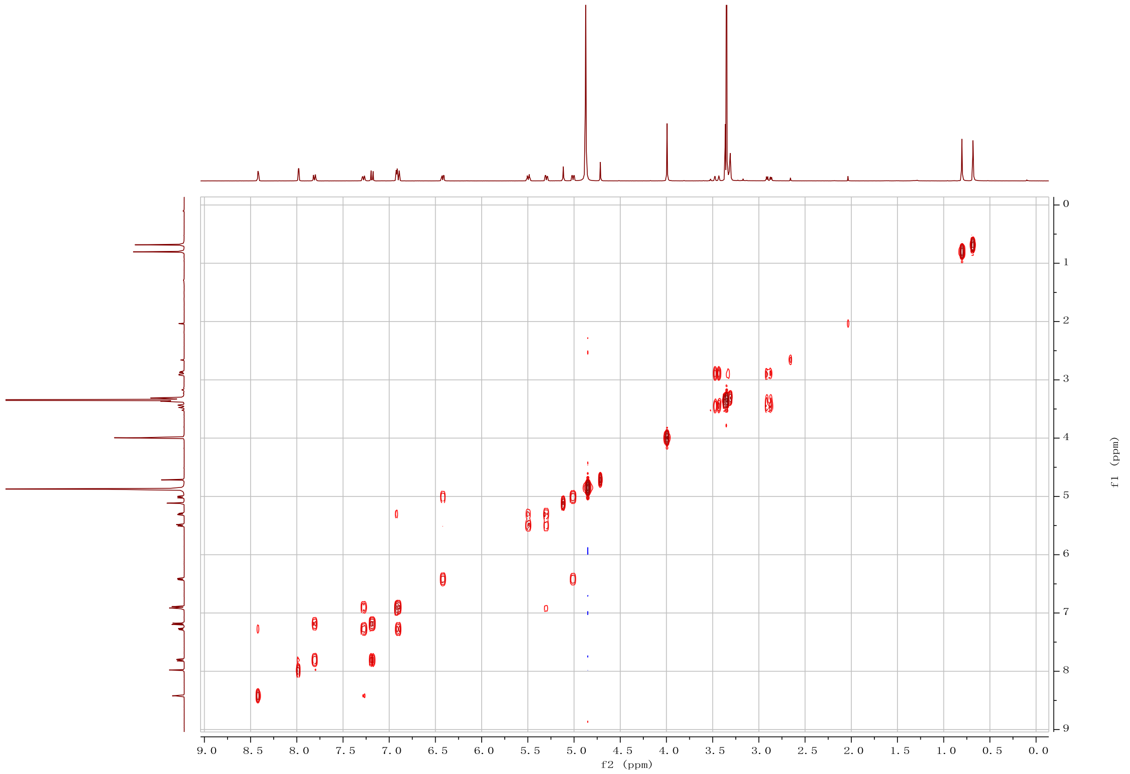


**Figure S8.** $^1H$–$^1H$ COSY spectrum of **1** recorded in $CD_3OD$

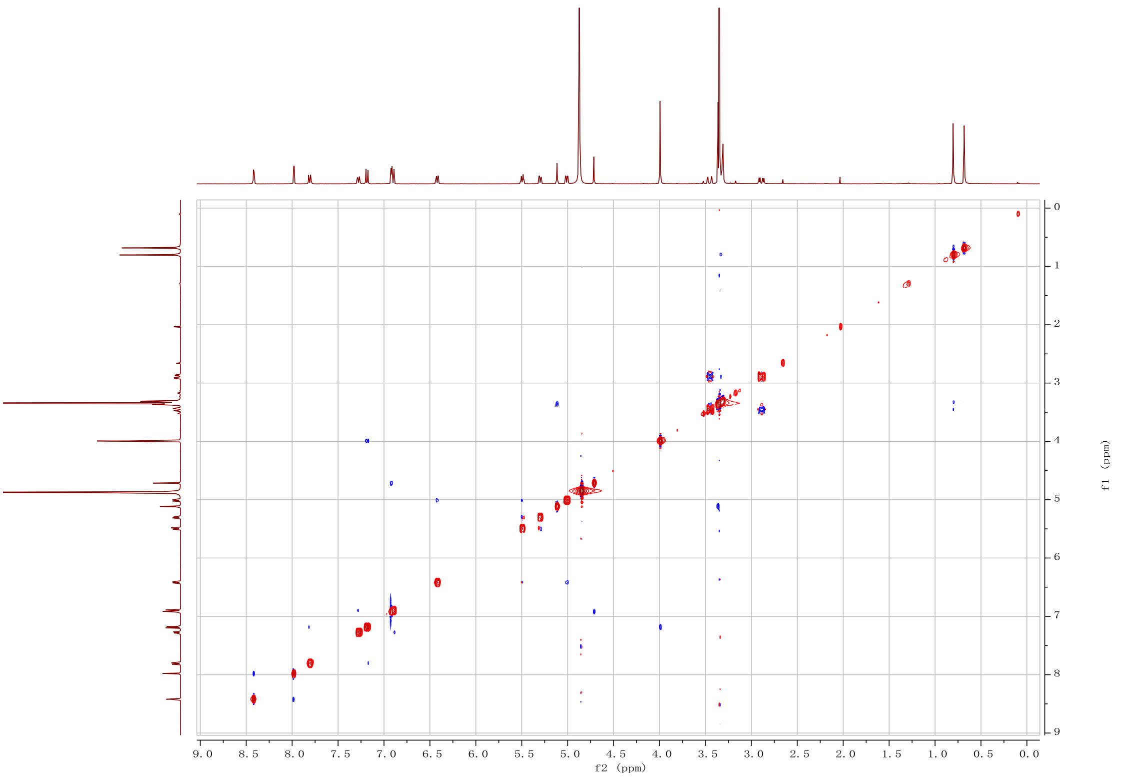


**Figure S9.** NOESY spectrum of **1** recorded in $CD_3OD$

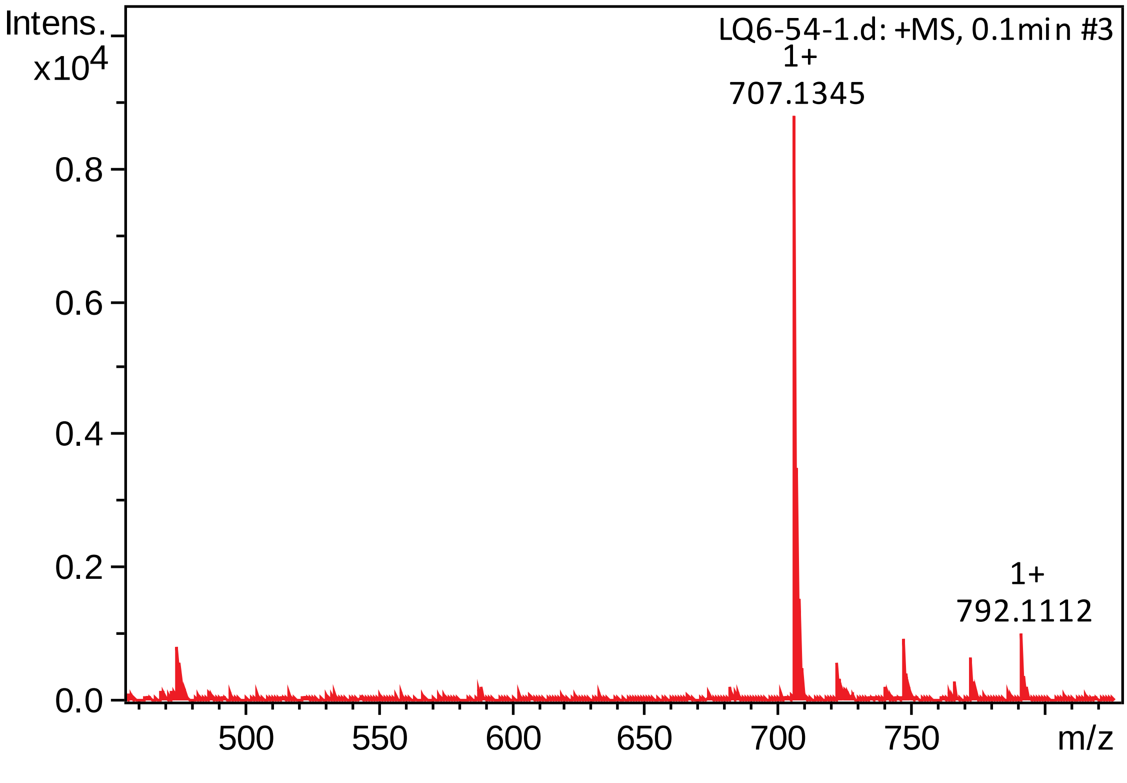


**Figure S10.** HRESIMS spectrum of **1**

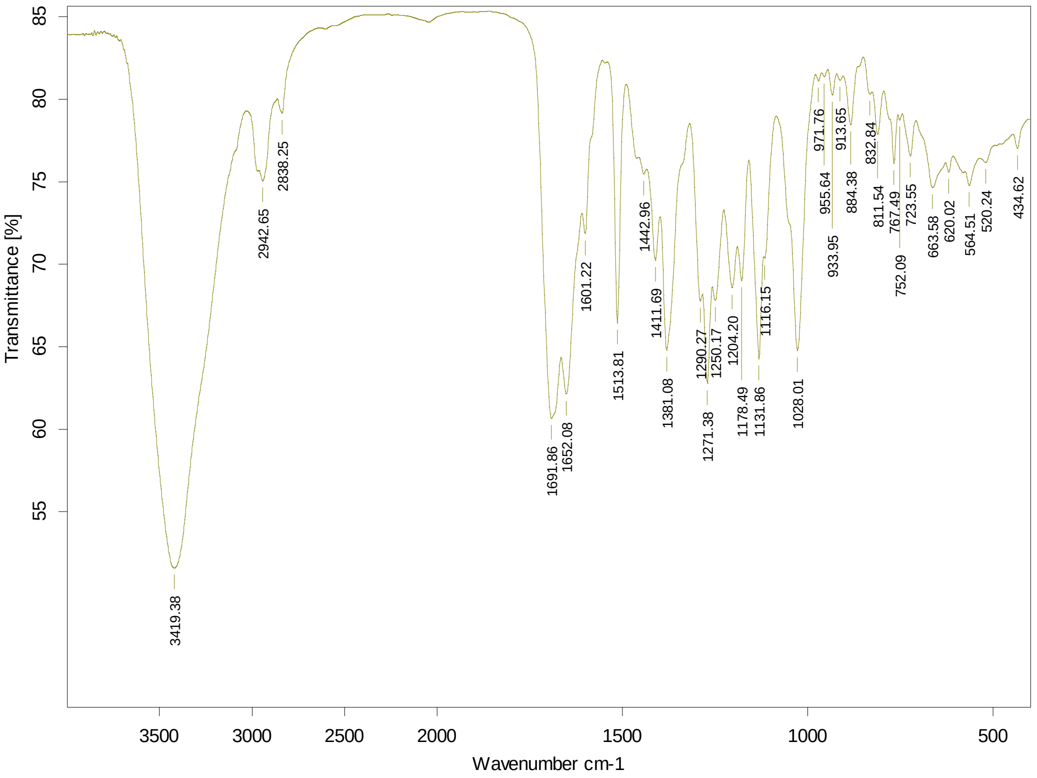


**Figure S11.** IR spectrum of **1**

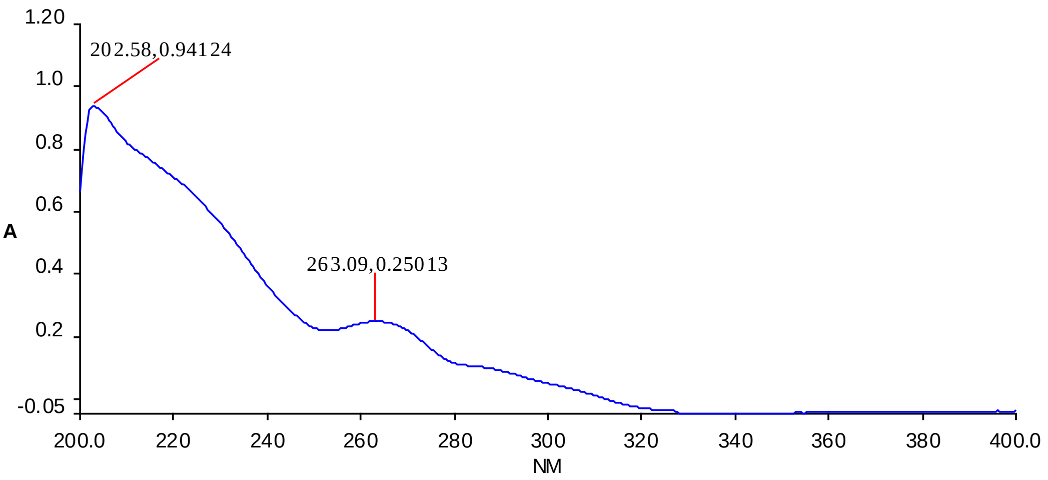


**Figure S12.** UV spectrum of **1**

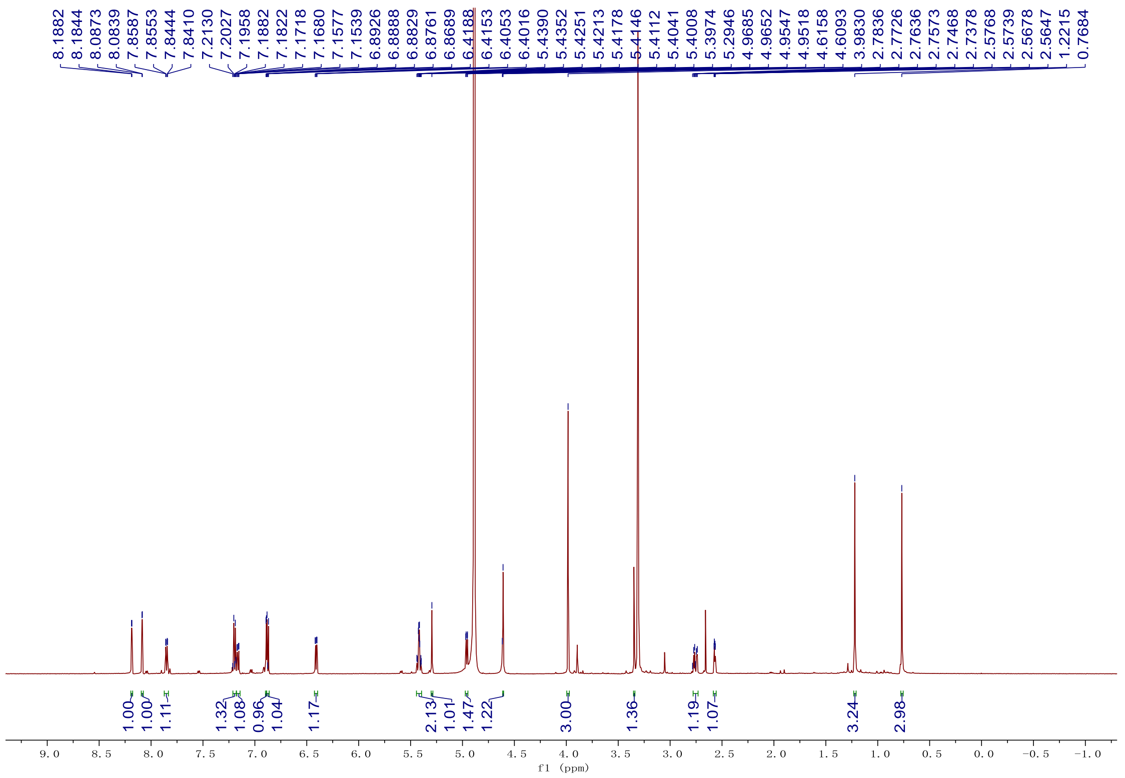


**Figure S13.** $^{1}H$ NMR (600 MHz) spectrum of **2** recorded in $CD_3OD$

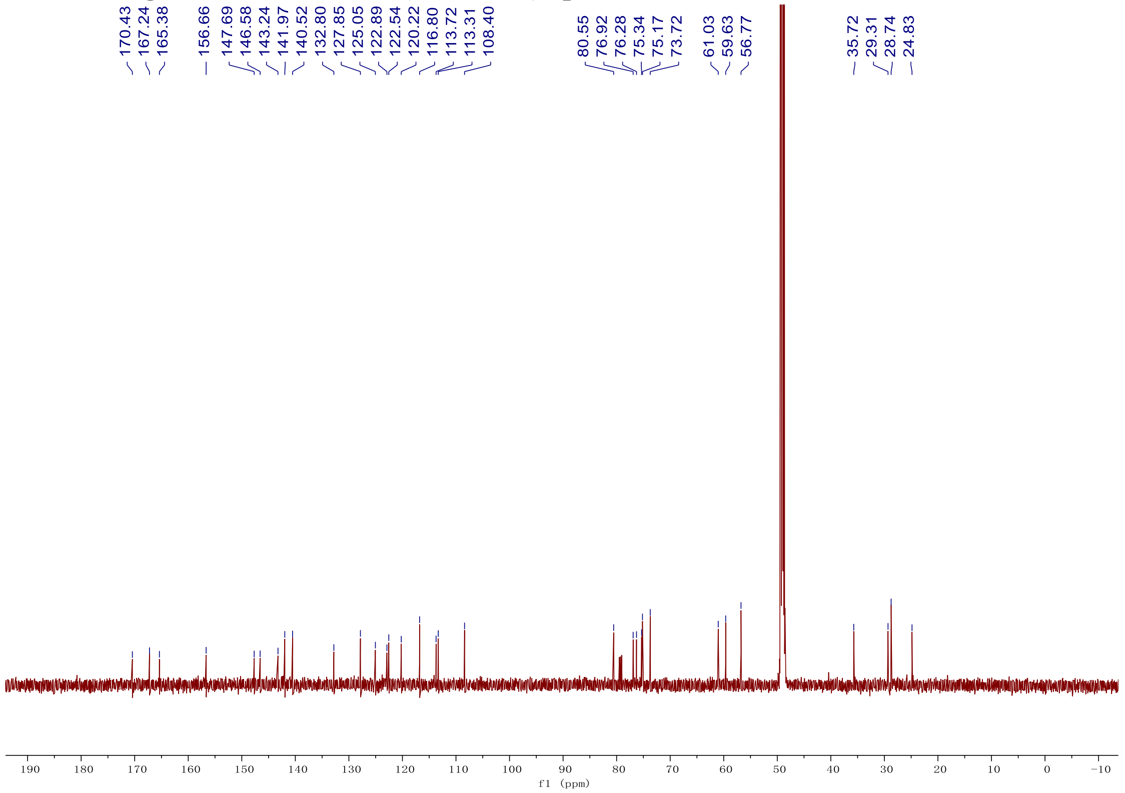


**Figure S14.** $^{13}C$ NMR (150 MHz) spectrum of **2** recorded in $CD_3OD$

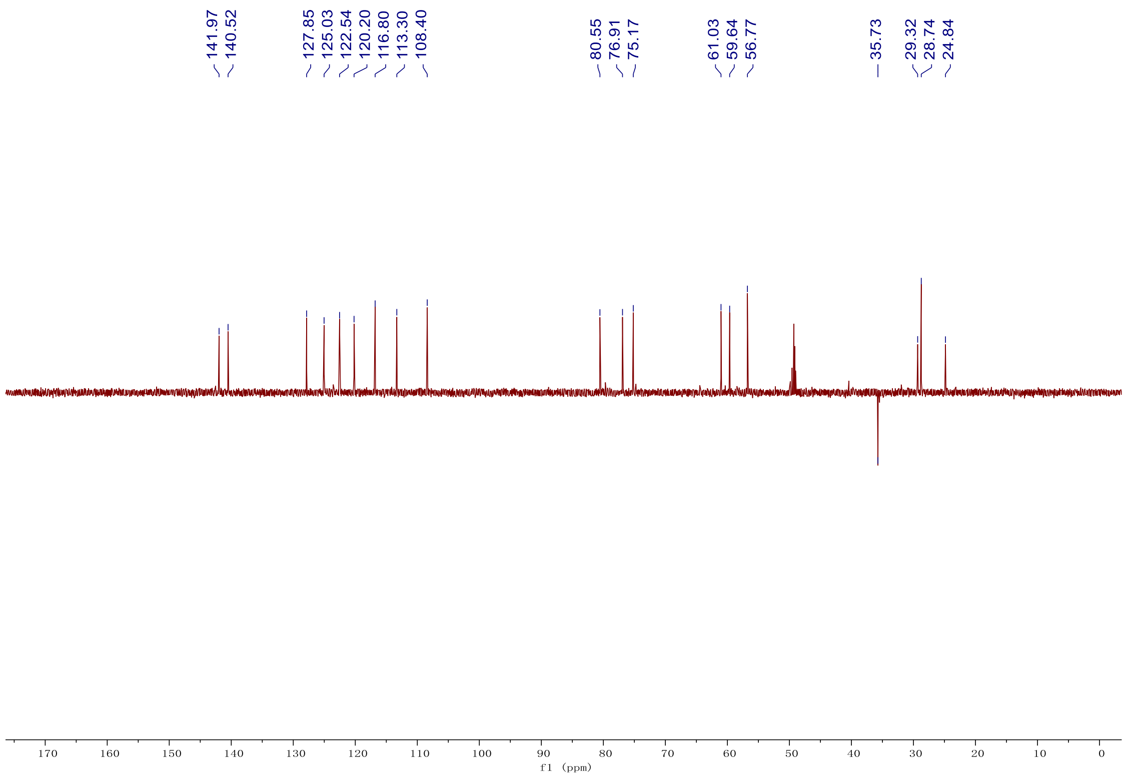


**Figure S15.** DEPT NMR spectrum of **2** recorded in $CD_3OD$

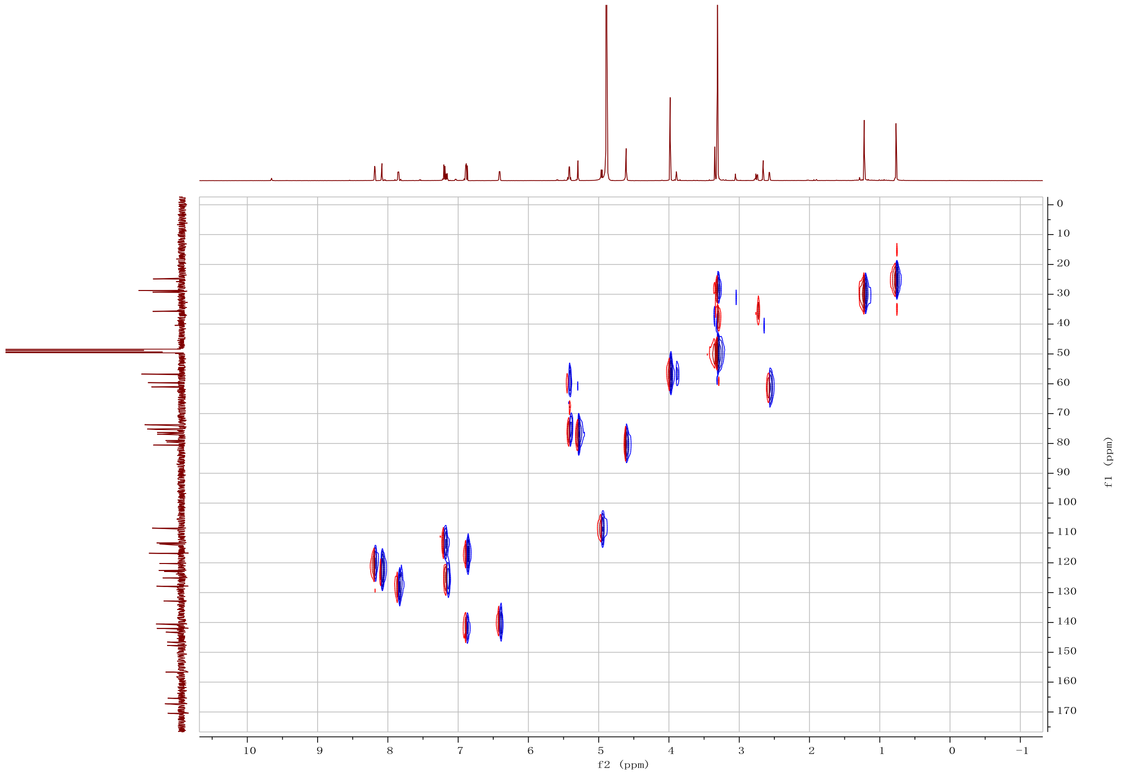


**Figure S16.** HSQC spectrum of **2** recorded in $CD_3OD$

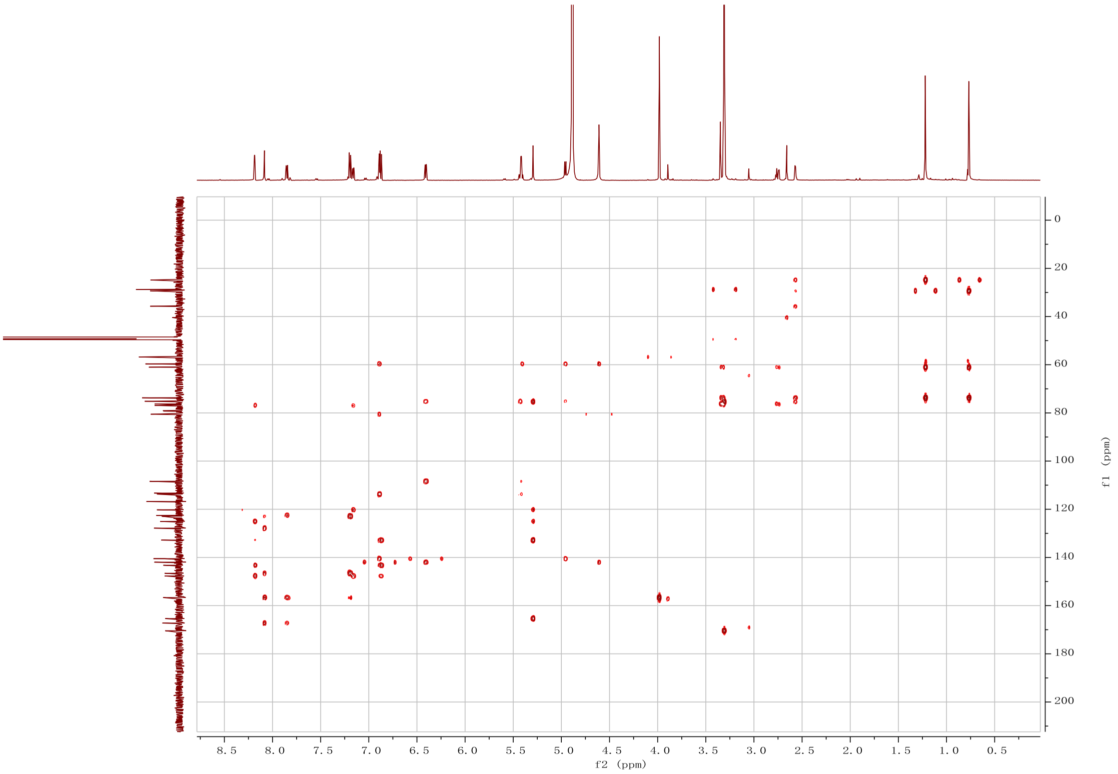


**Figure S17.** HMBC spectrum of **2** recorded in $CD_3OD$

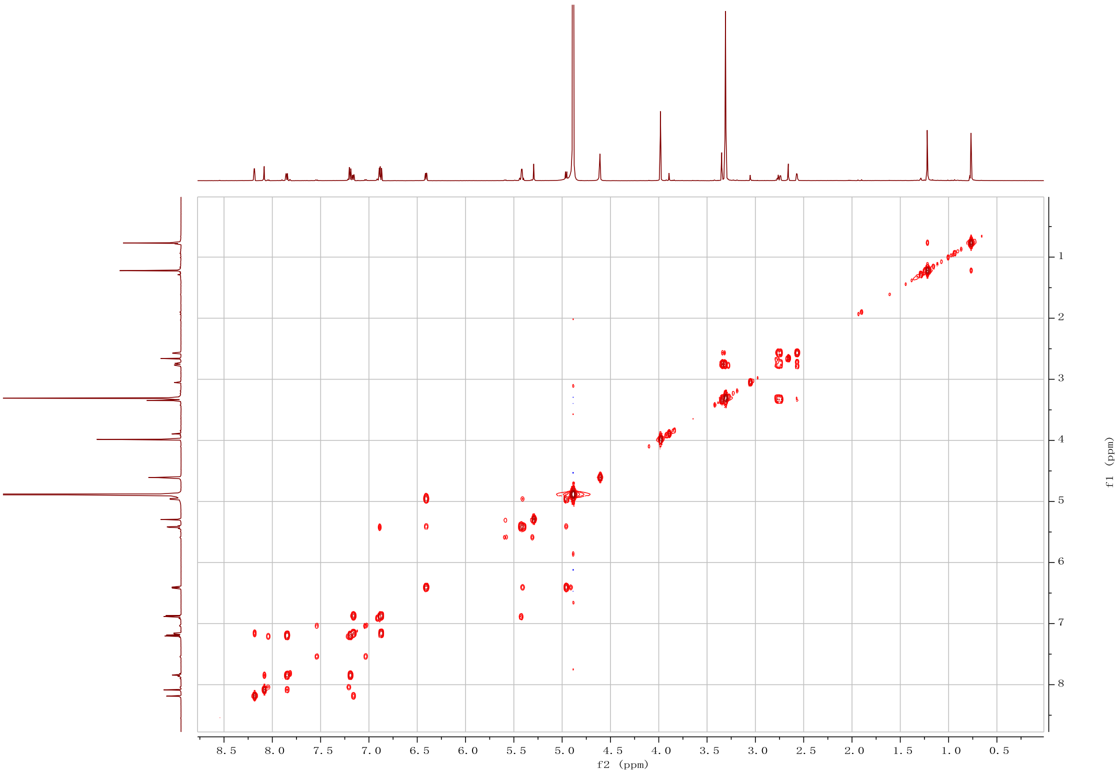


**Figure S18.** $^1H$–$^1H$ COSY spectrum of **2** recorded in $CD_3OD$

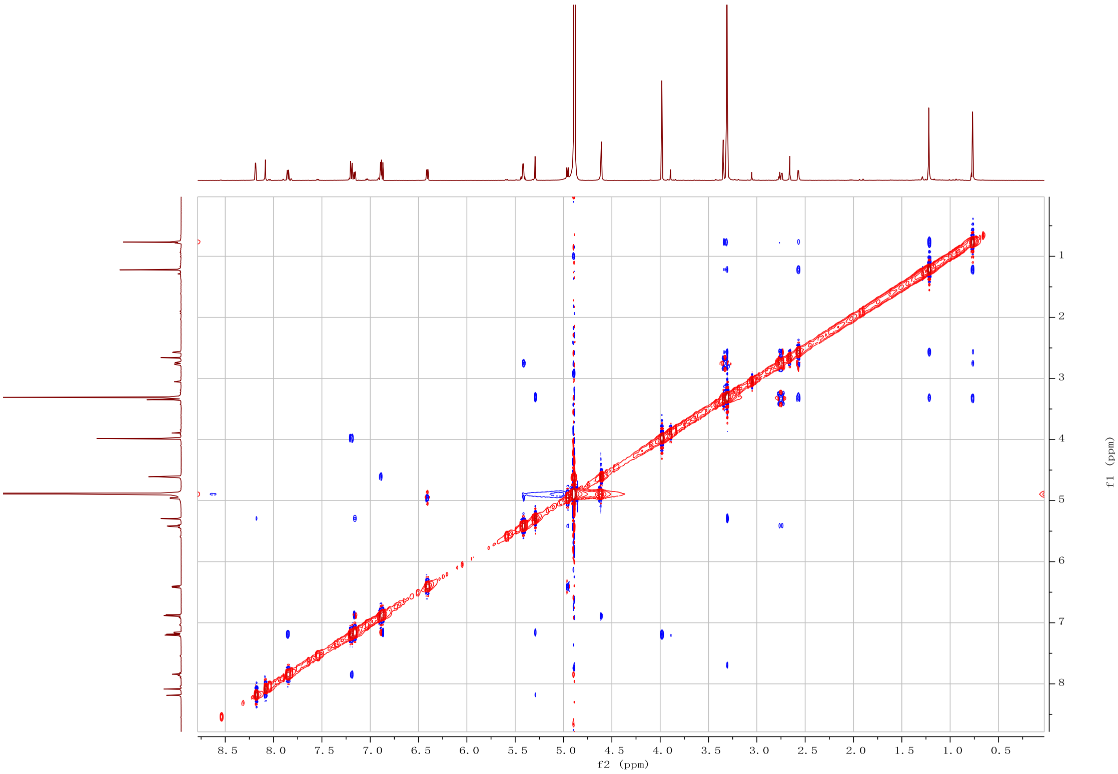


**Figure S 19.** NOESY spectrum of **2** recorded in $CD_3OD$

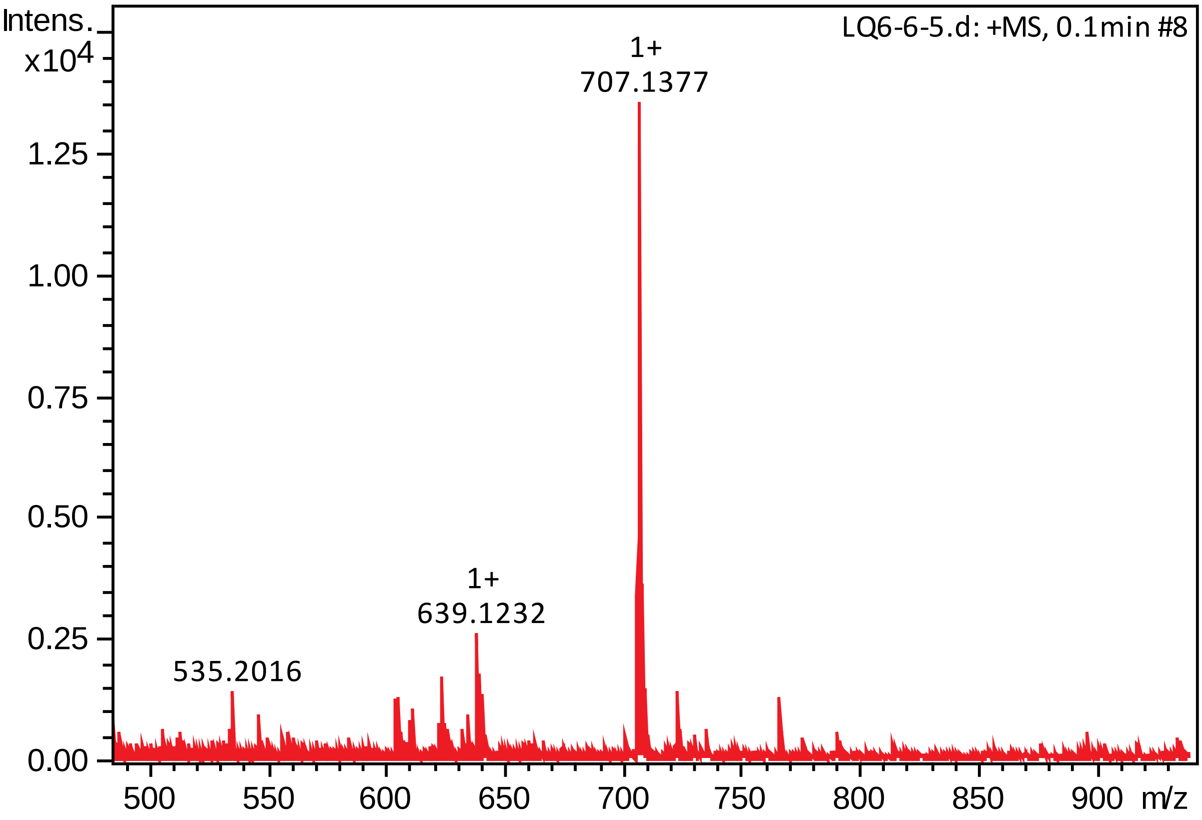


**Figure S20.** HRESIMS spectrum of **2**

E:\20181008\20181008李琴\lq6-6-5.0 16:03:49 2018-10-8

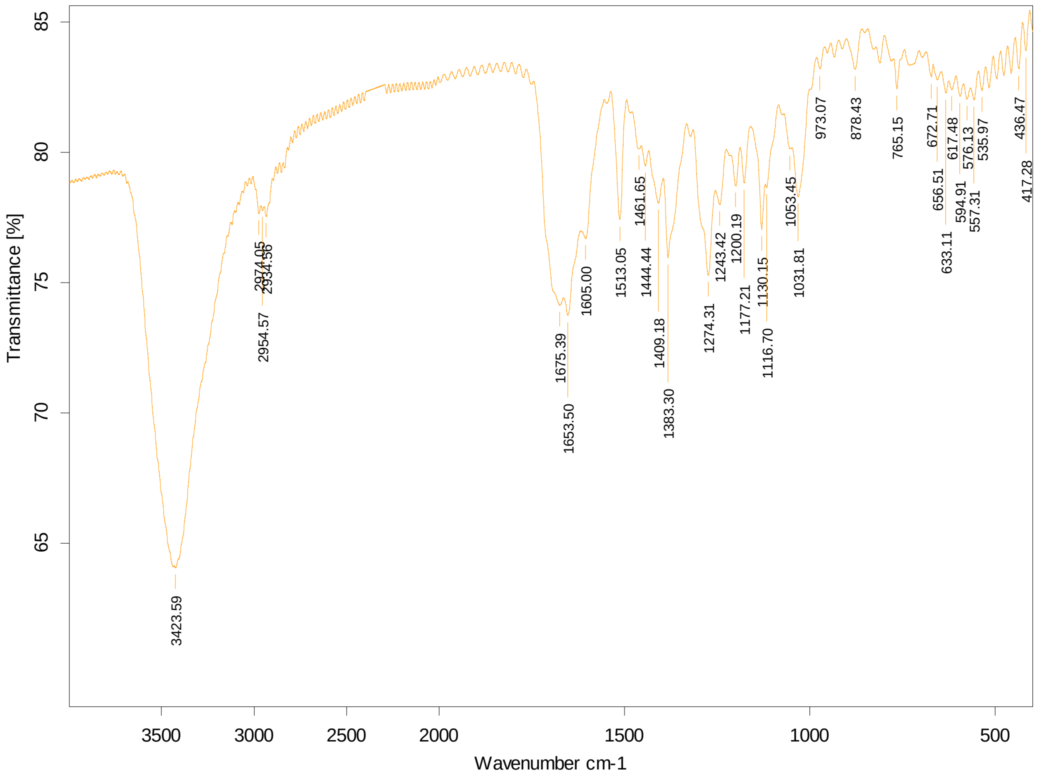


**Figure S21.** IR spectrum of **2**

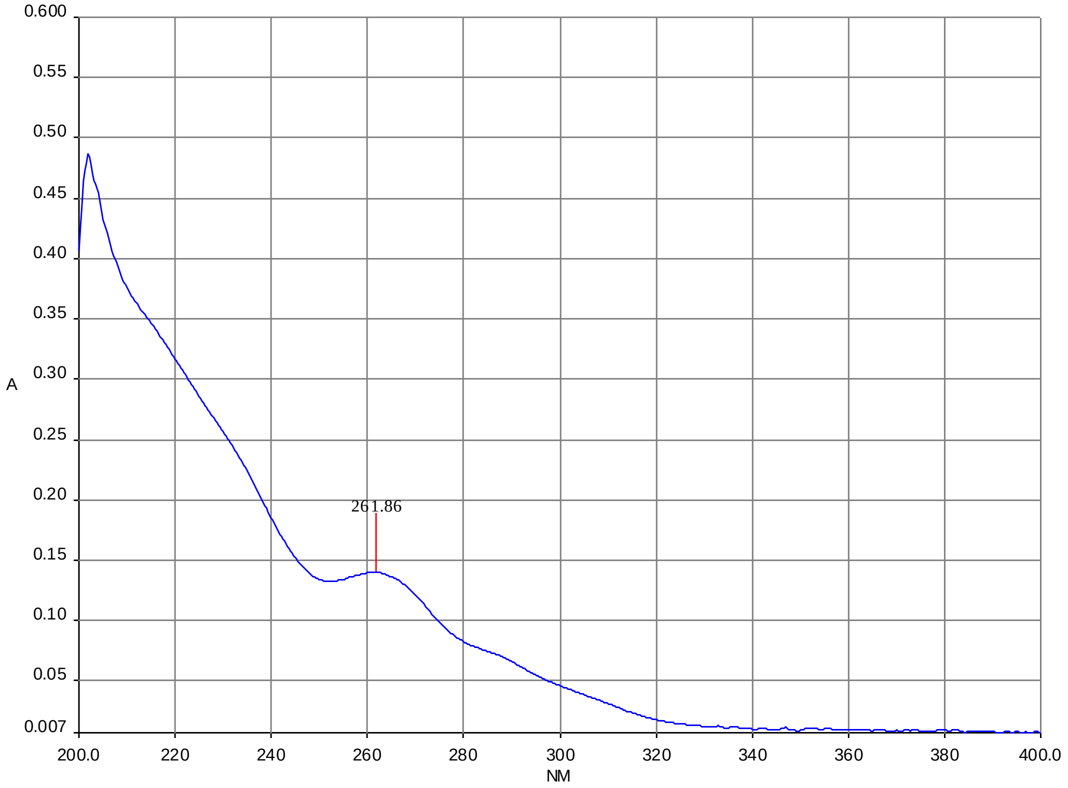


**Figure S22.** UV spectrum of **2**

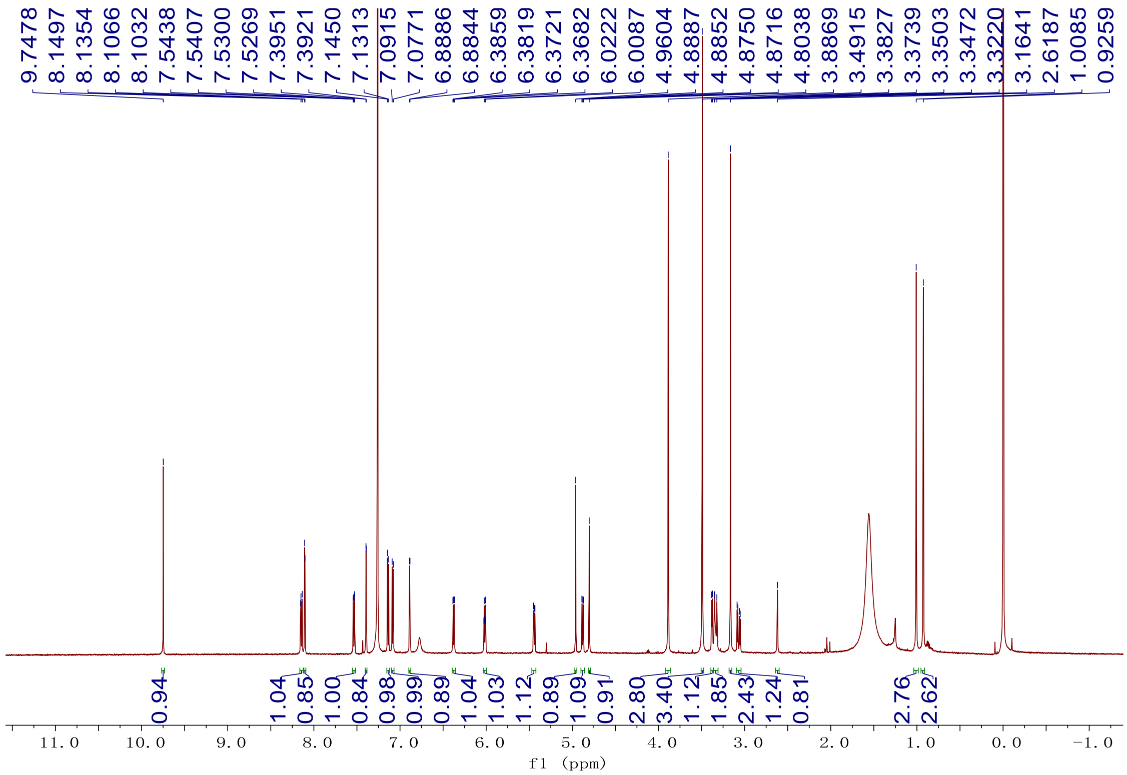


**Figure S23.** $^{1}$H NMR (600 MHz) spectrum of **3** recorded in $CDCl_3$

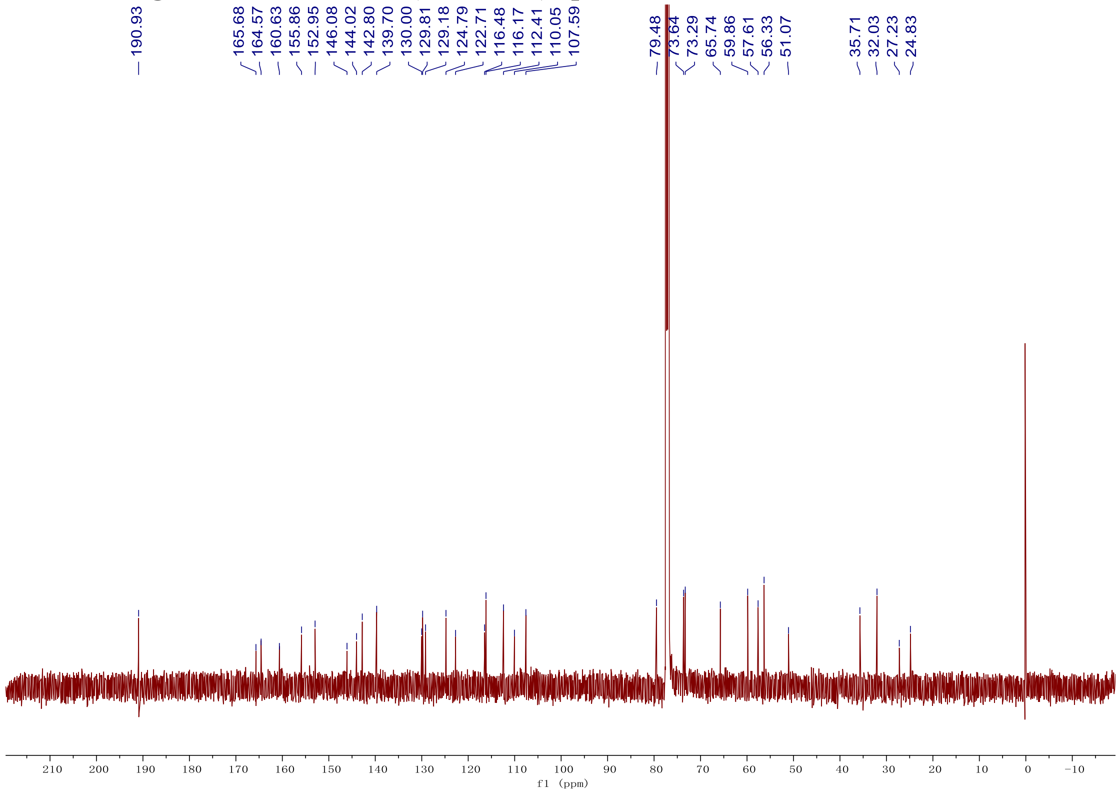


**Figure S24.** $^{13}$C NMR (150 MHz) spectrum of **3** recorded in $CDCl_3$

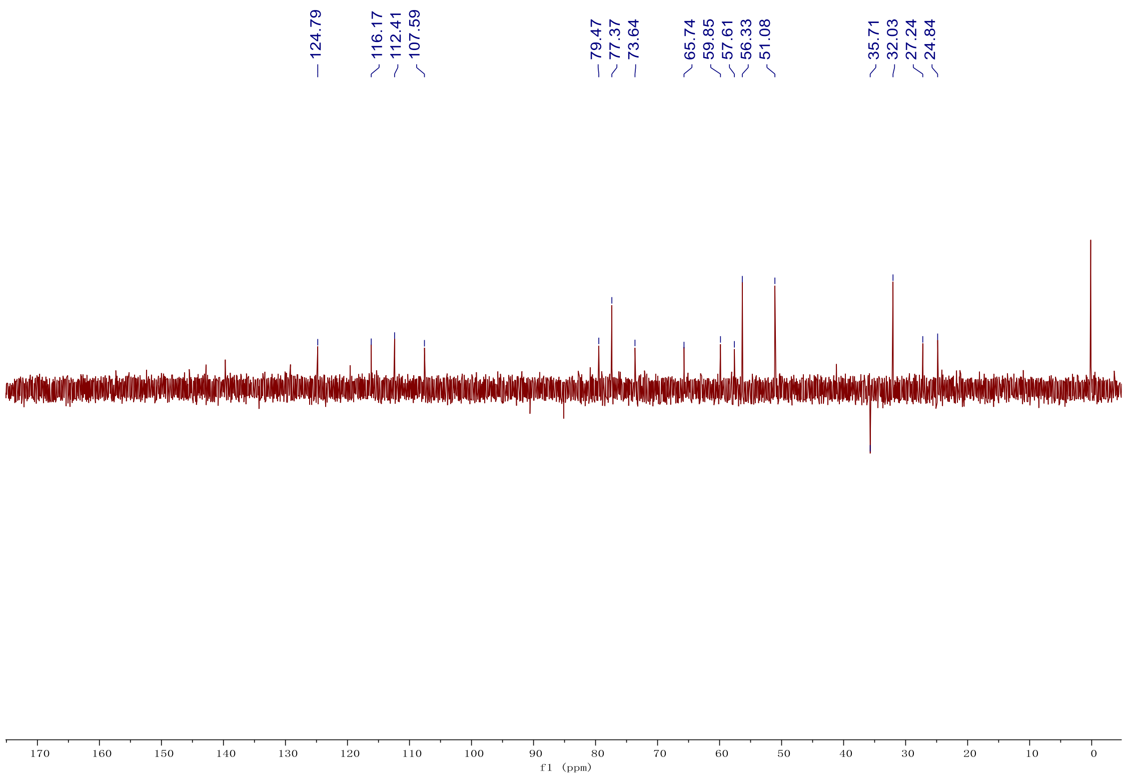


**Figure S25.** DEPT NMR spectrum of **3** recorded in $CDCl_3$

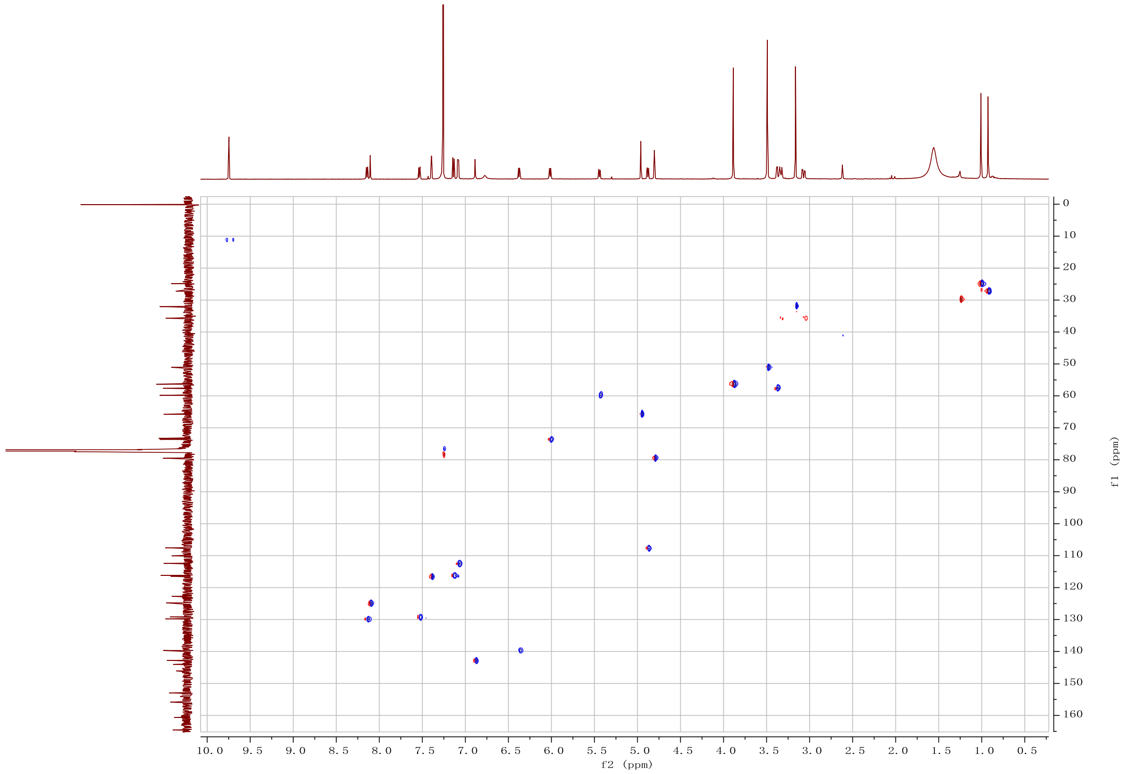


**Figure S26.** HSQC spectrum of **3** recorded in $CDCl_3$

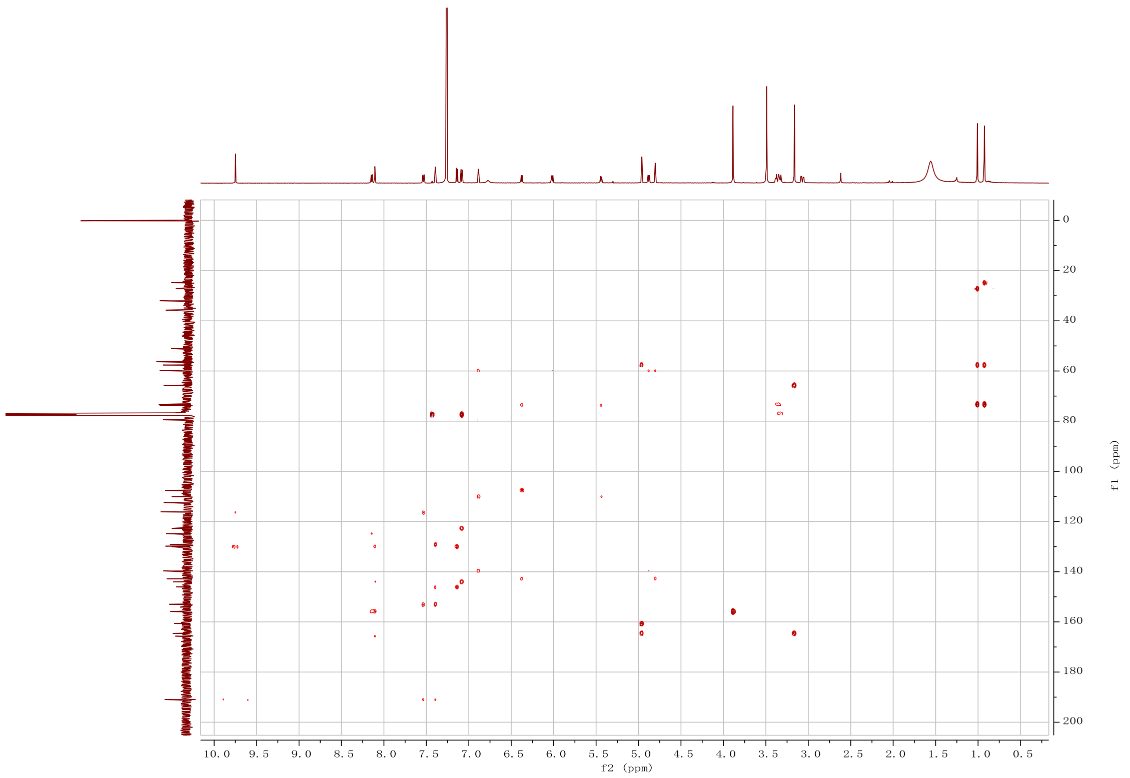


**Figure S27.** HMBC spectrum of **3** recorded in $CDCl_3$

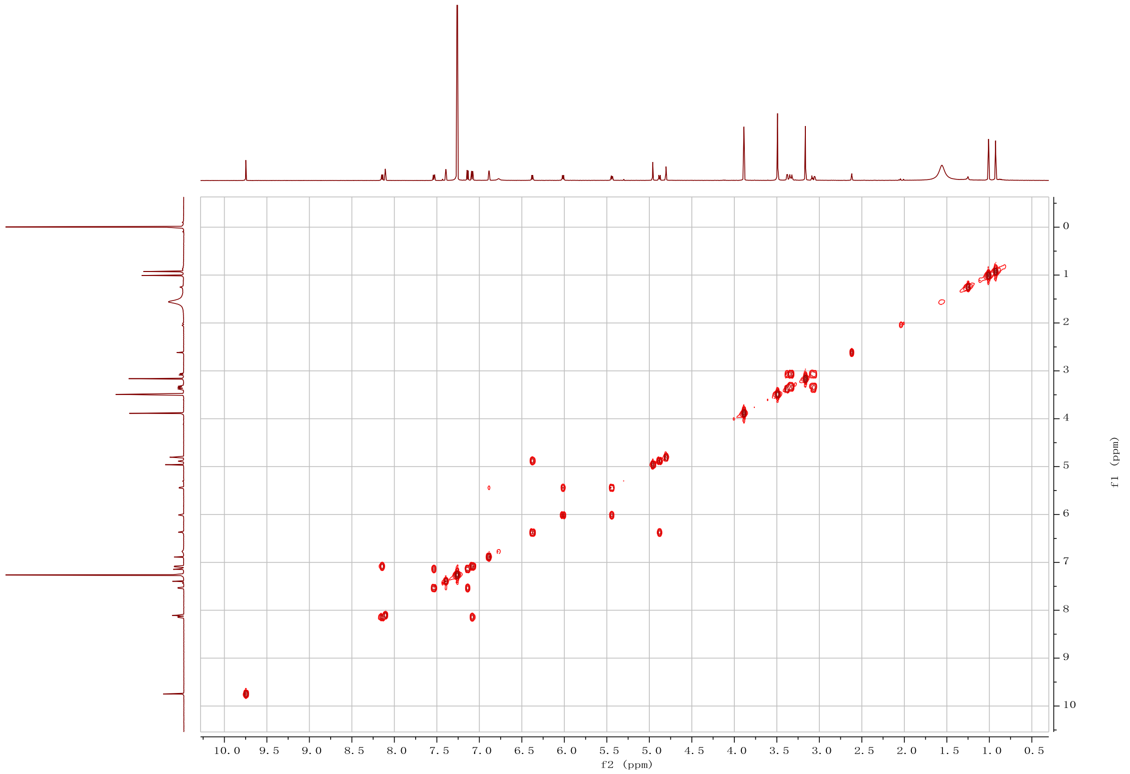


**Figure S28.** $^1H$–$^1H$ COSY spectrum of **3** recorded in $CDCl_3$

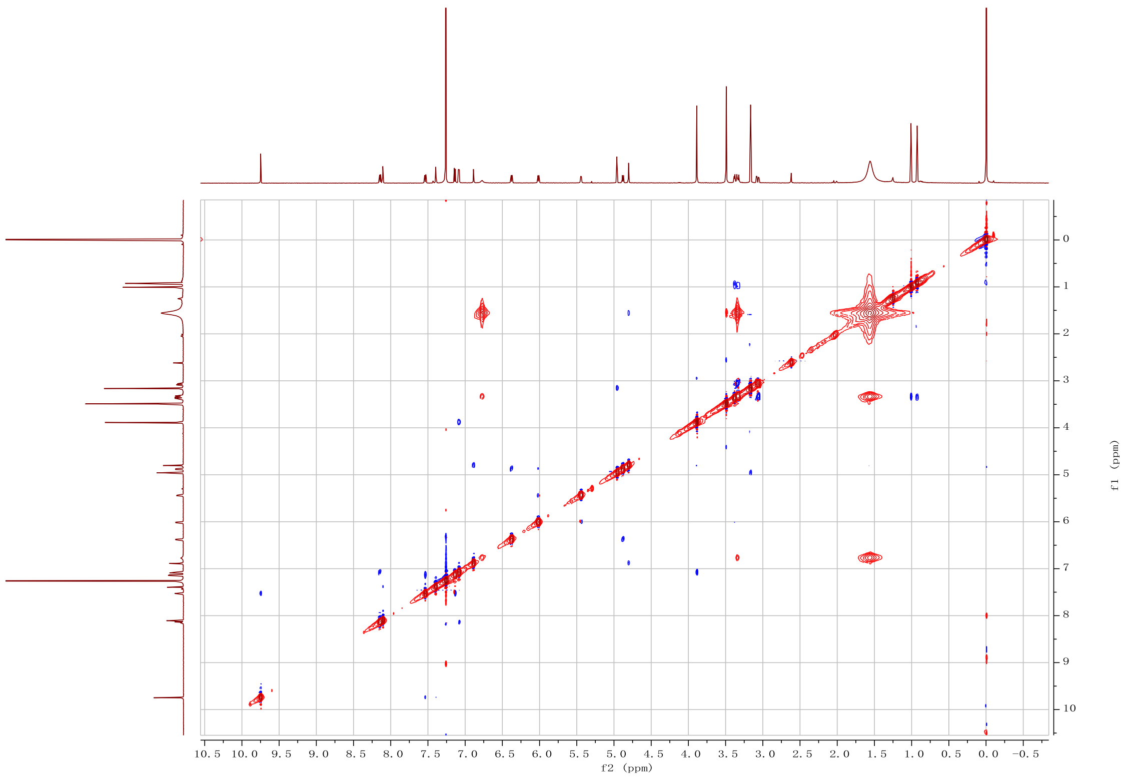


**Figure S29.** NOESY spectrum of **3** recorded in $CDCl_3$

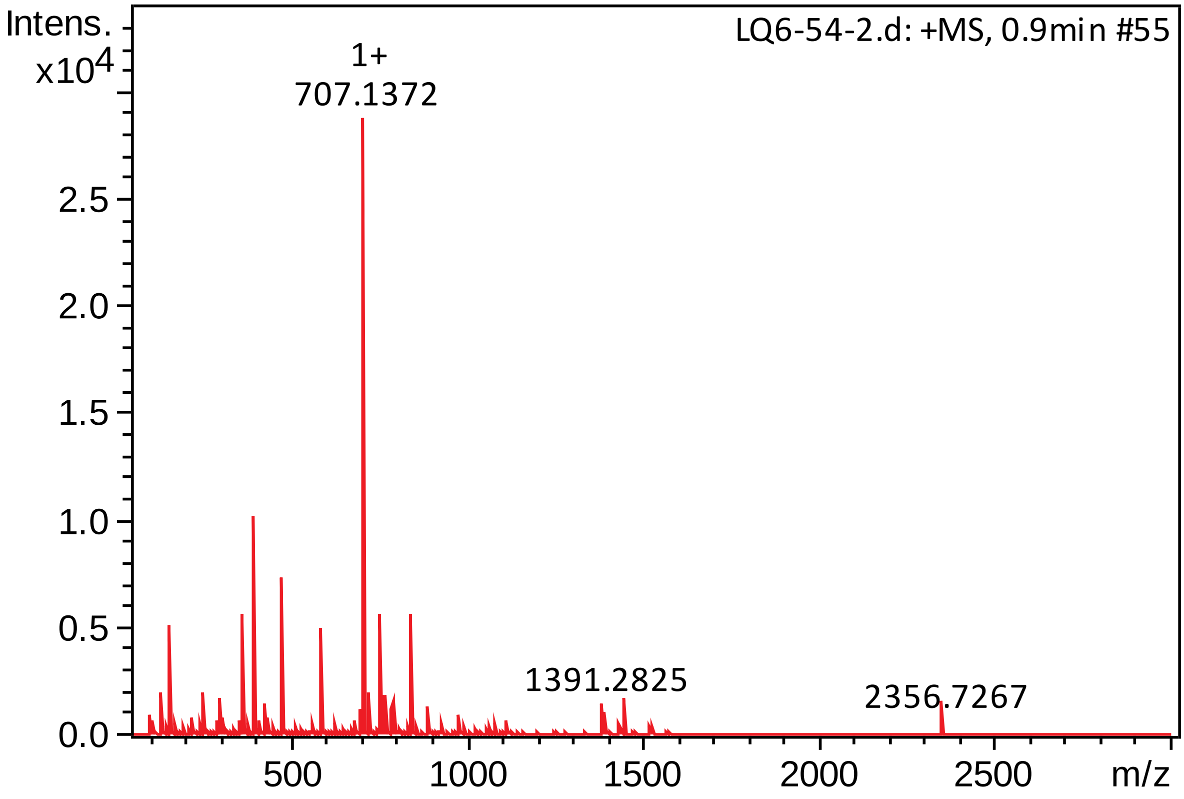


**Figure S30.** HRESIMS spectrum of **3**

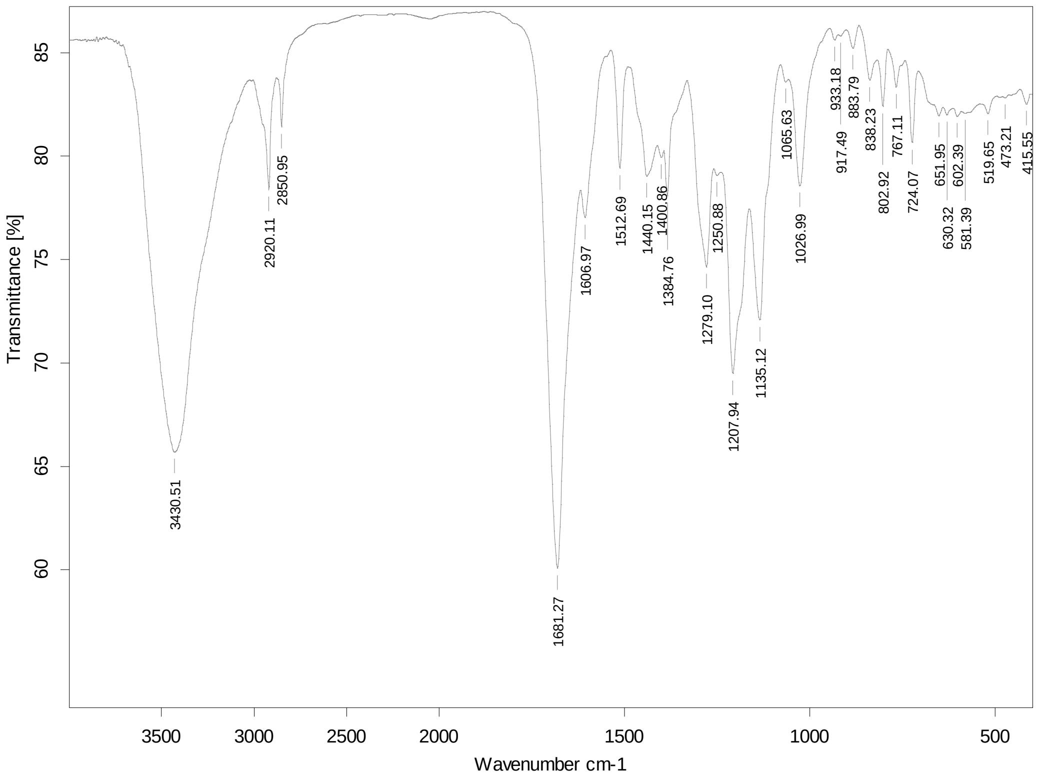


**Figure S31.** IR spectrum of **3**

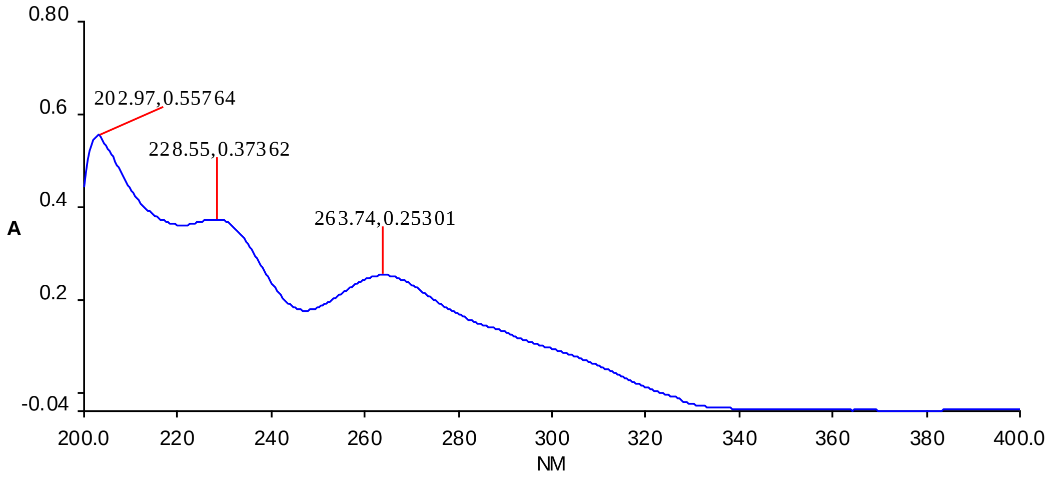


**Figure S32.** UV spectrum of **3**

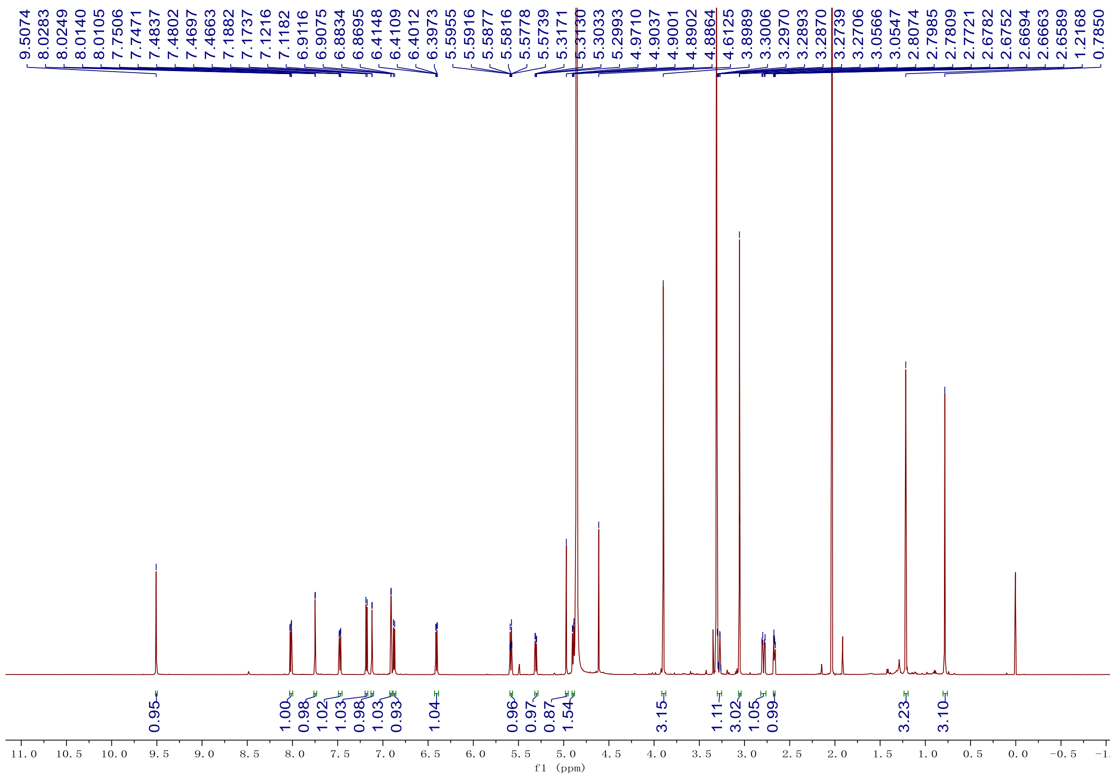

**Figure S33.** $^{1}H$ NMR (600 MHz) spectrum of **4** recorded in $CD_3OD$

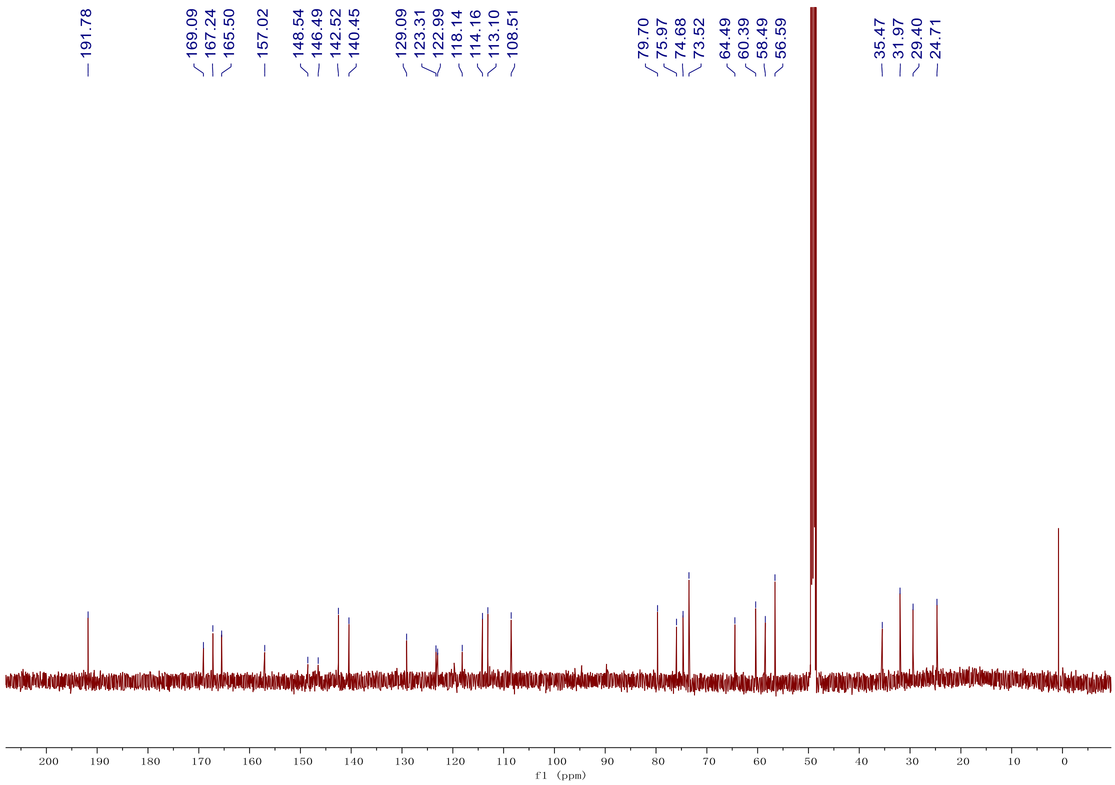

**Figure S34.** $^{13}C\{^{1}H\}$ NMR (150 MHz) spectrum of **4** recorded in $CD_3OD$

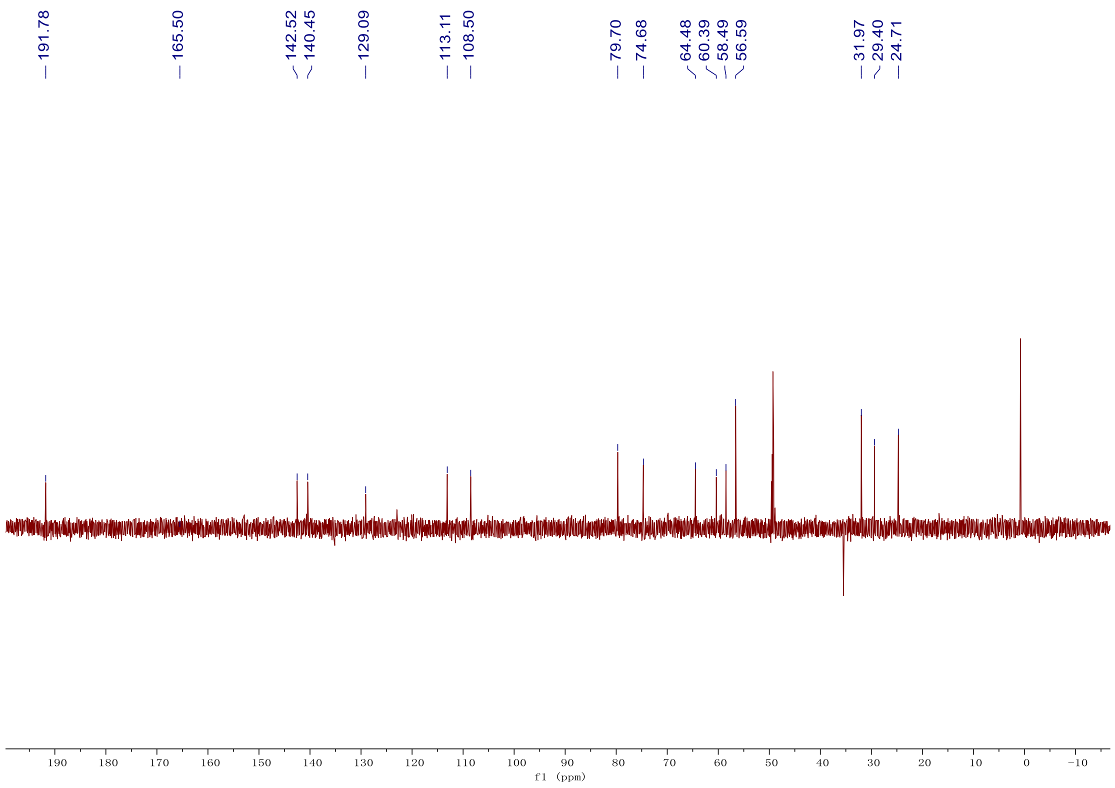


**Figure S35.** DEPT NMR spectrum of **4** recorded in $CD_3OD$

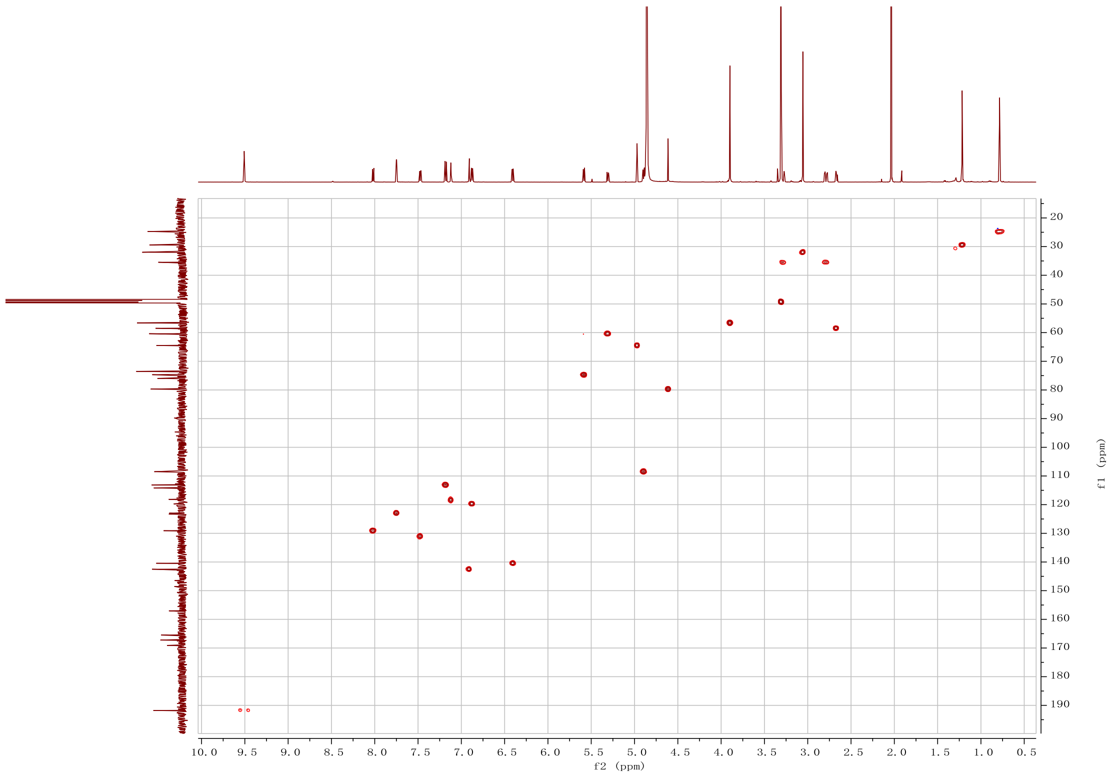


**Figure S36.** HSQC spectrum of **4** recorded in $CD_3OD$

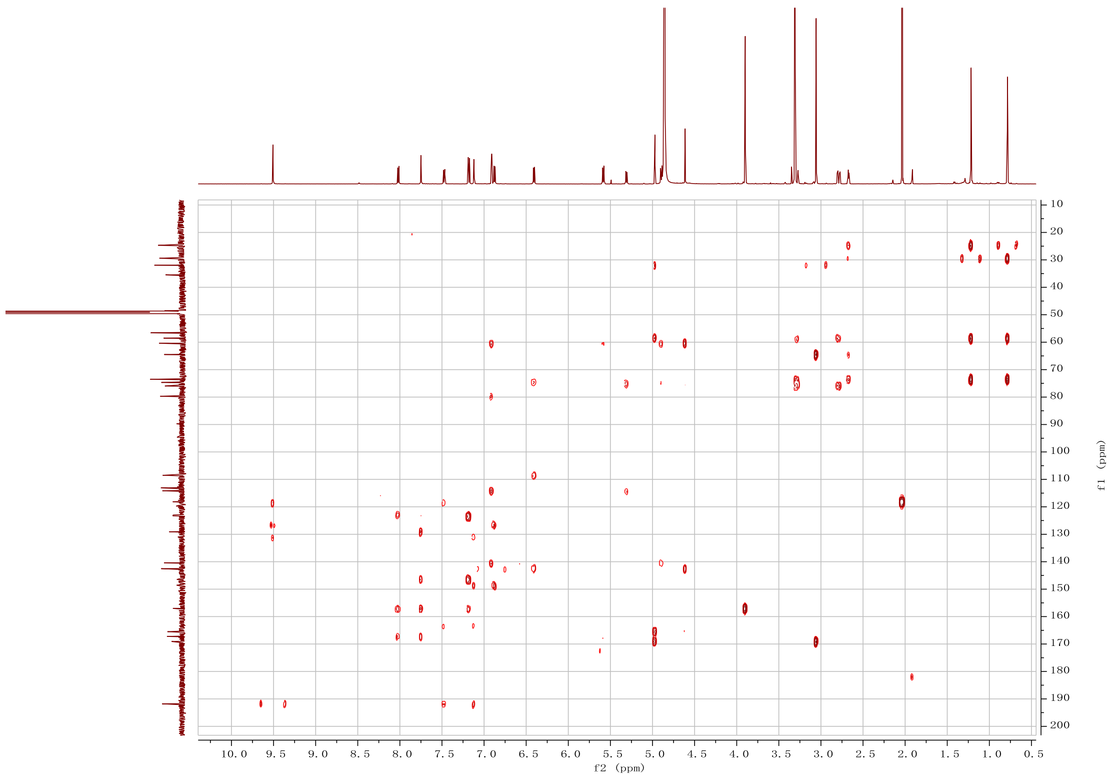


**Figure S37.** HMBC spectrum of **4** recorded in $CD_3OD$

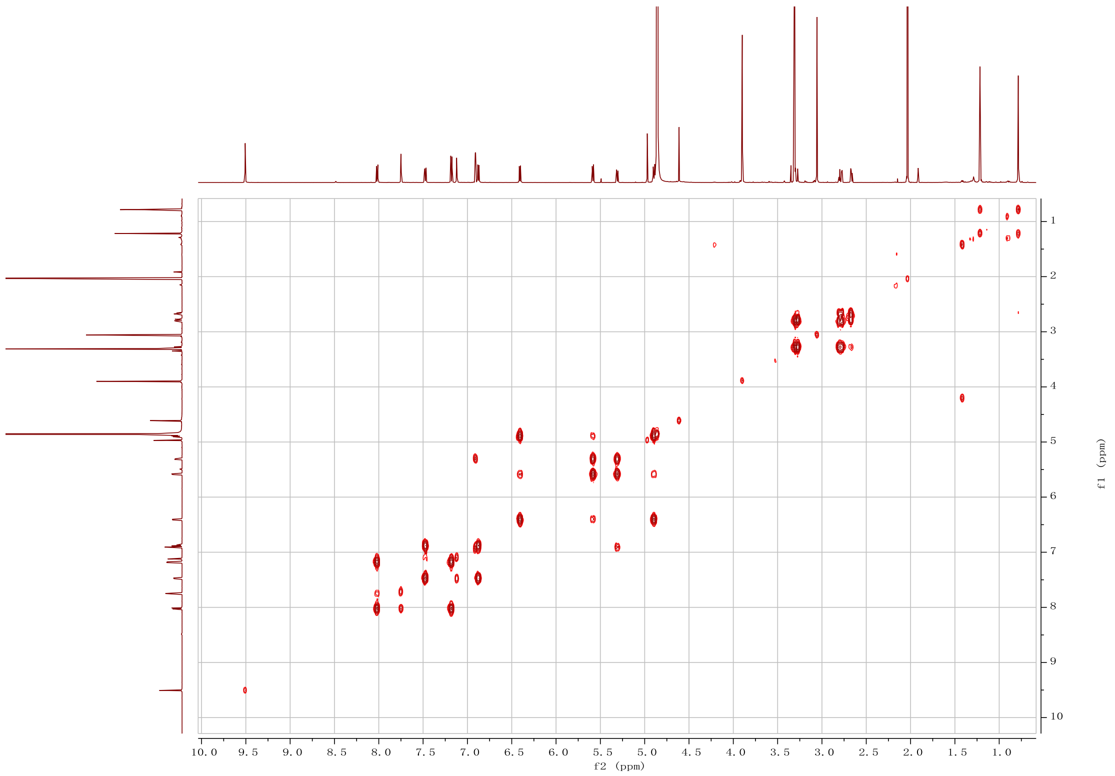


**Figure S38.** $^1H$–$^1H$ COSY spectrum of **4** recorded in $CD_3OD$

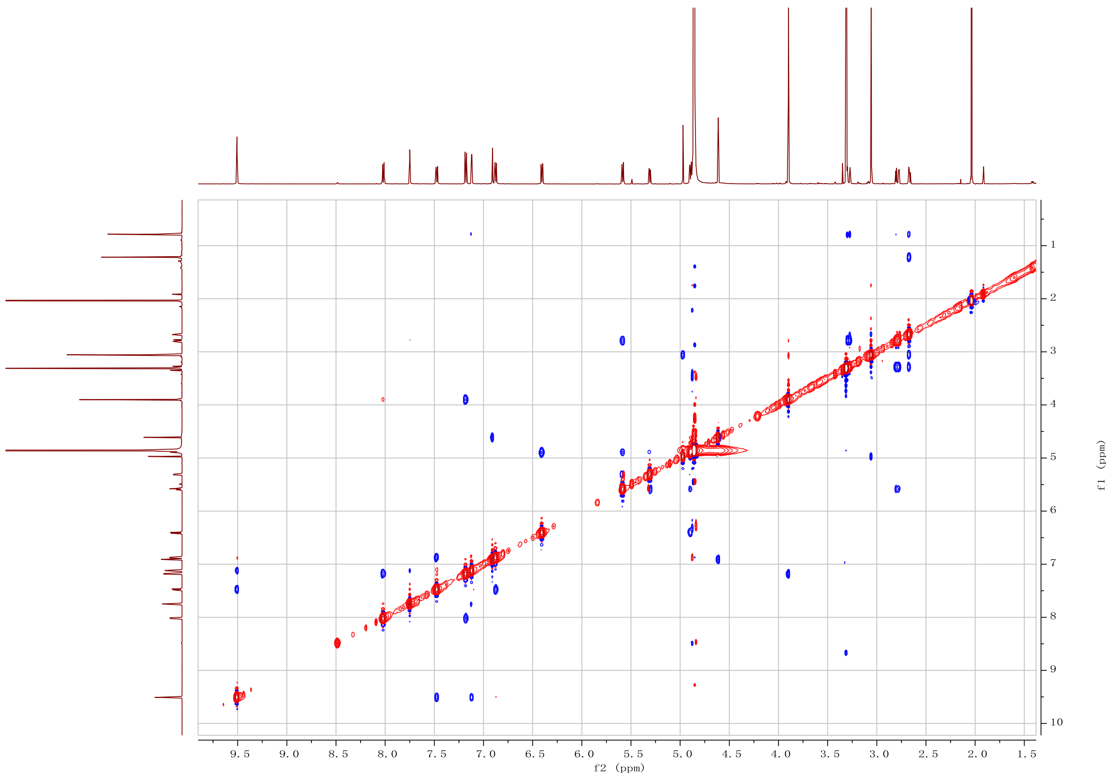


**Figure S39.** NOESY spectrum of **4** recorded in $CD_3OD$

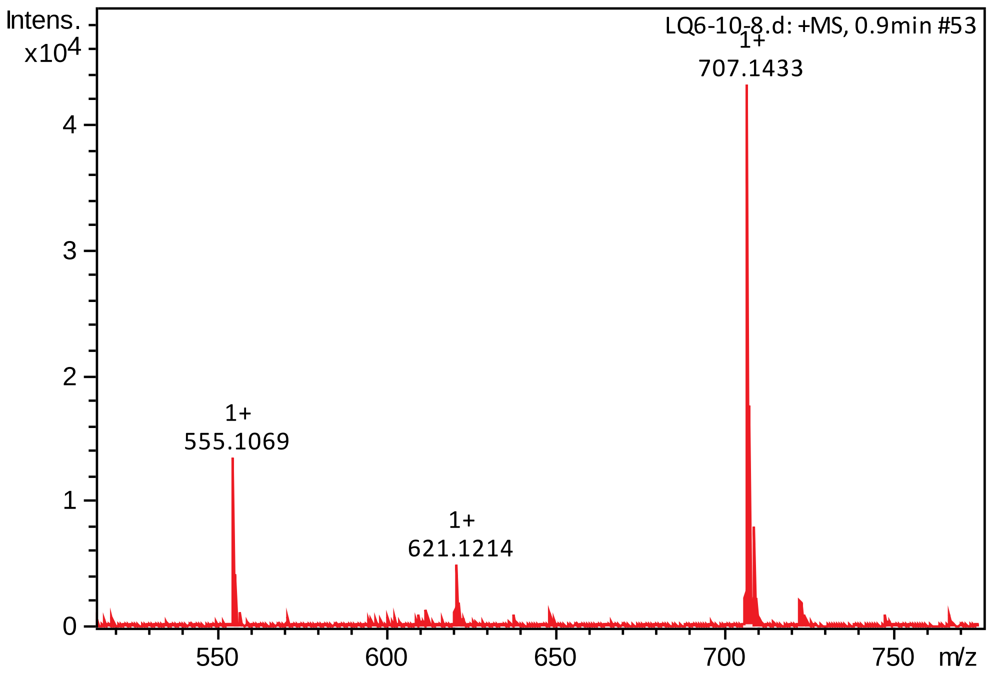


**Figure S40.** HRESIMS spectrum of **4**

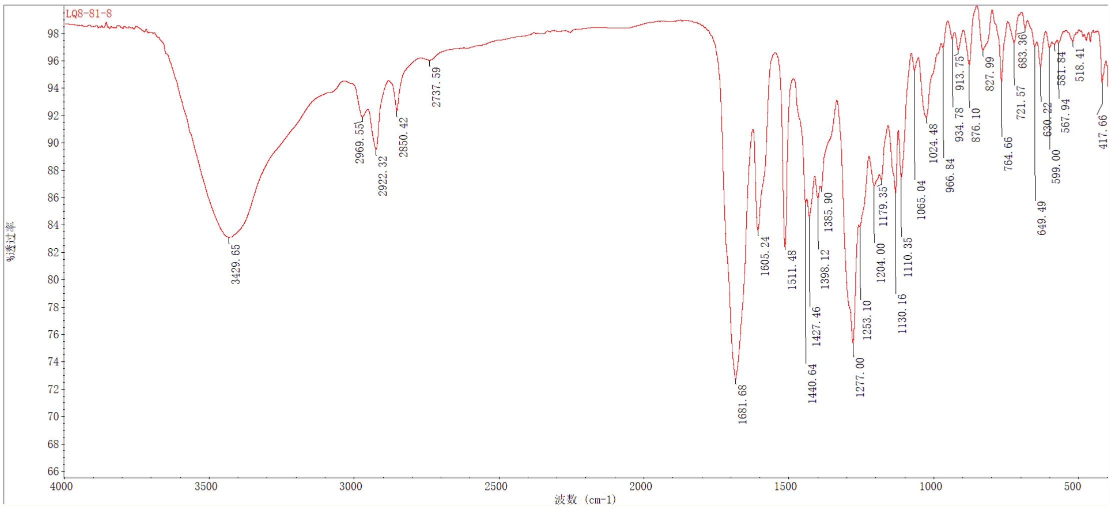


**Figure S41.** IR spectrum of **4**

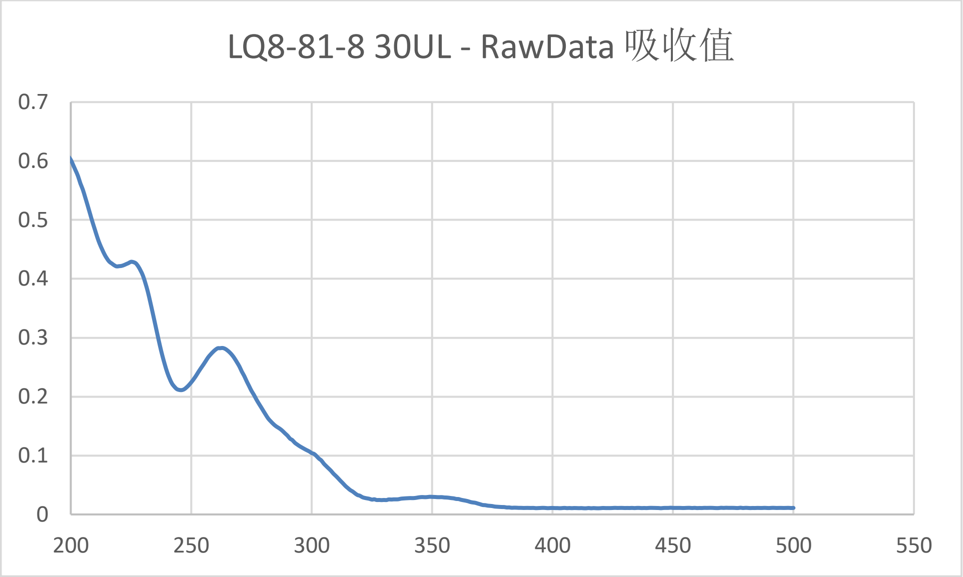


**Figure S42.** UV spectrum of **4**

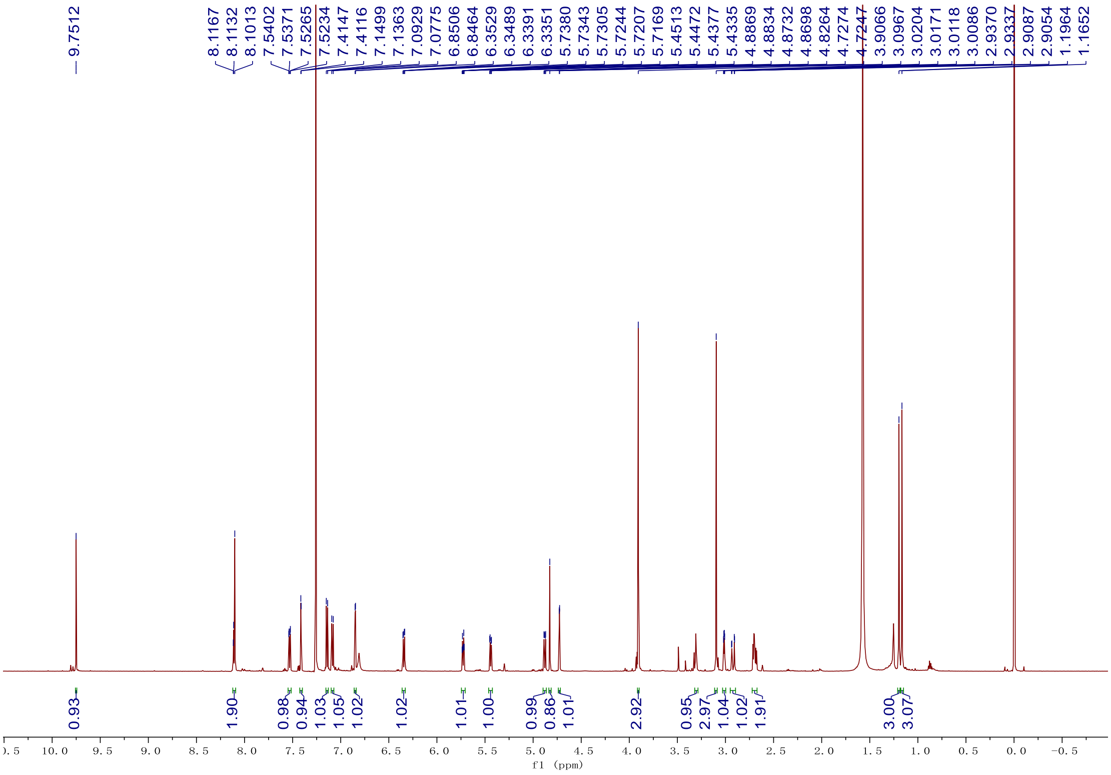


**Figure S43.** $^{1}$H NMR (600 MHz) spectrum of **5** recorded in $CDCl_3$

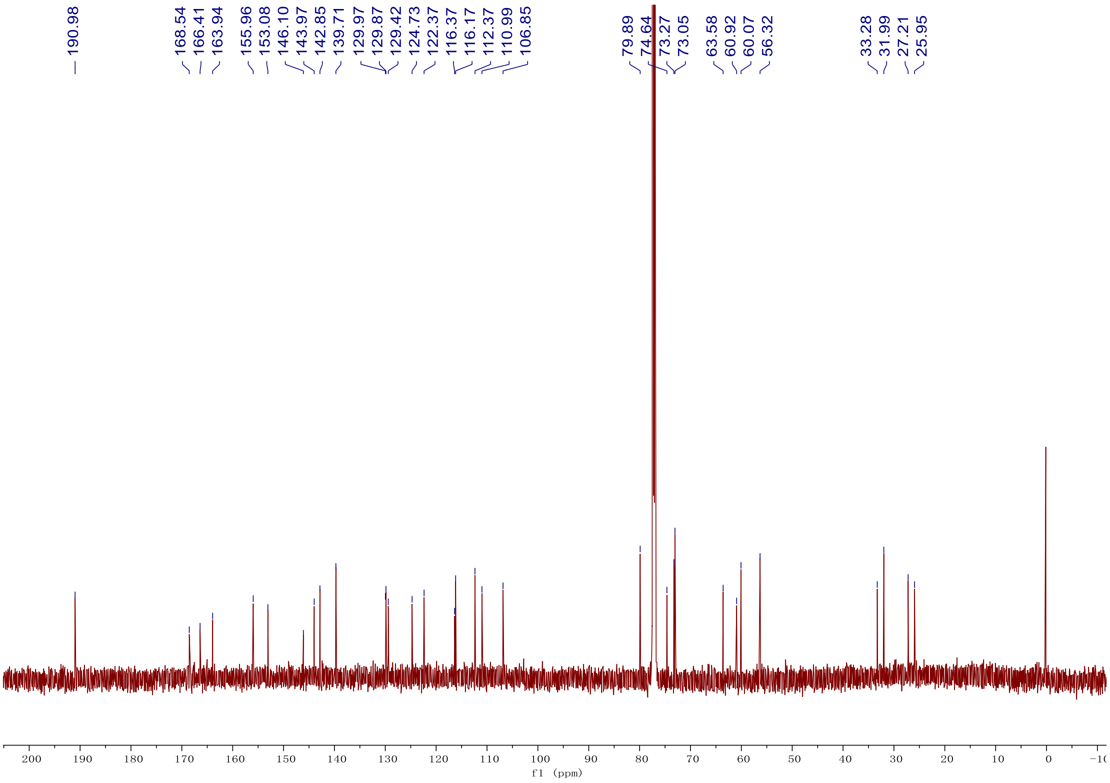


**Figure S44.** $^{13}$C NMR (150 MHz) spectrum of **5** recorded in $CDCl_3$

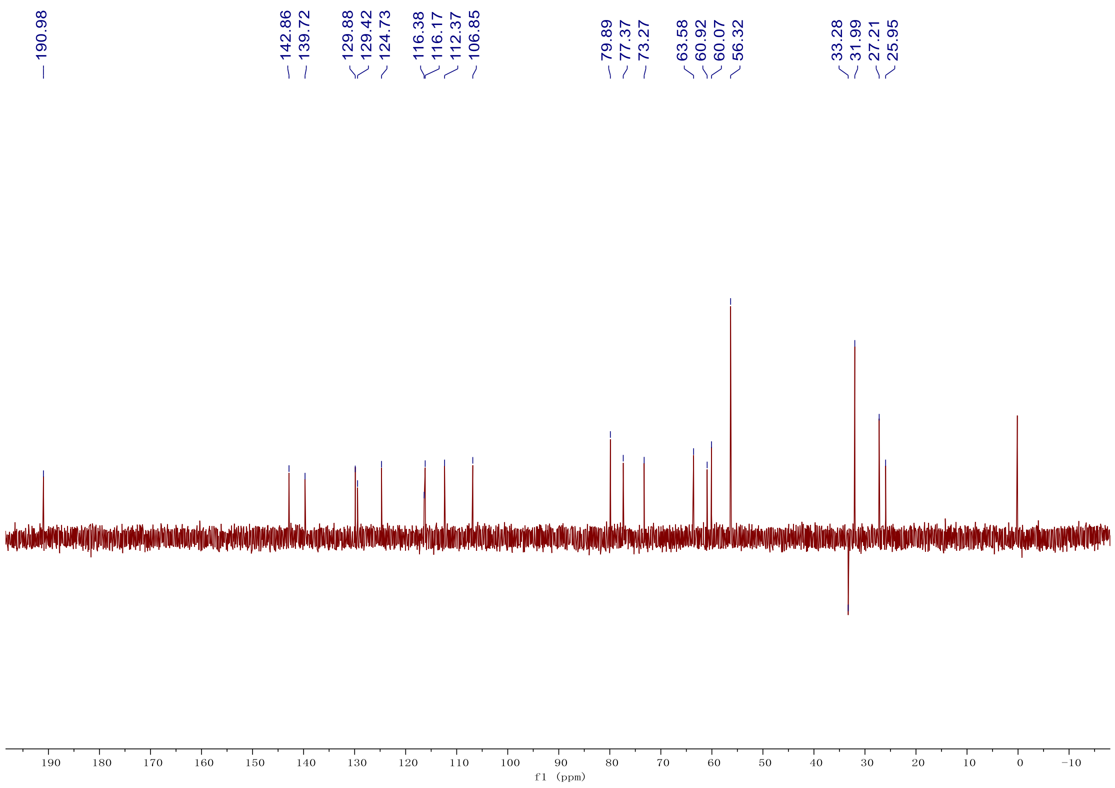


**Figure S45.** DEPT NMR spectrum of **5** recorded in $CDCl_3$

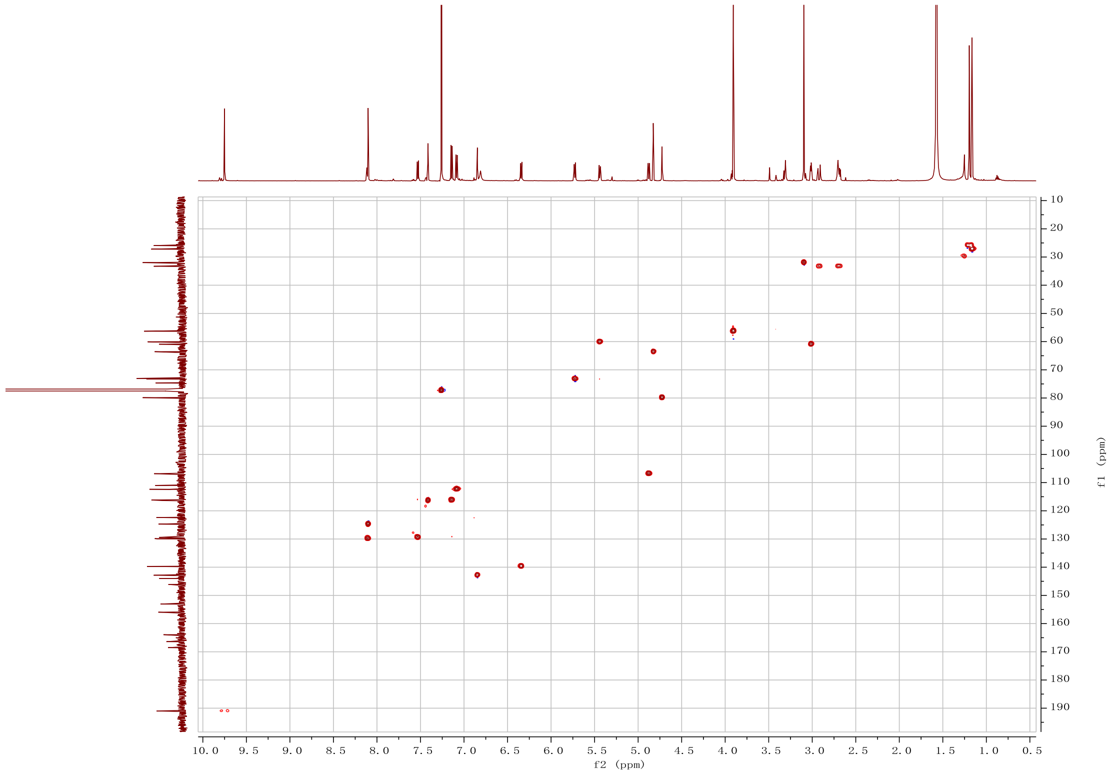


**Figure S46.** HSQC spectrum of **5** recorded in $CDCl_3$

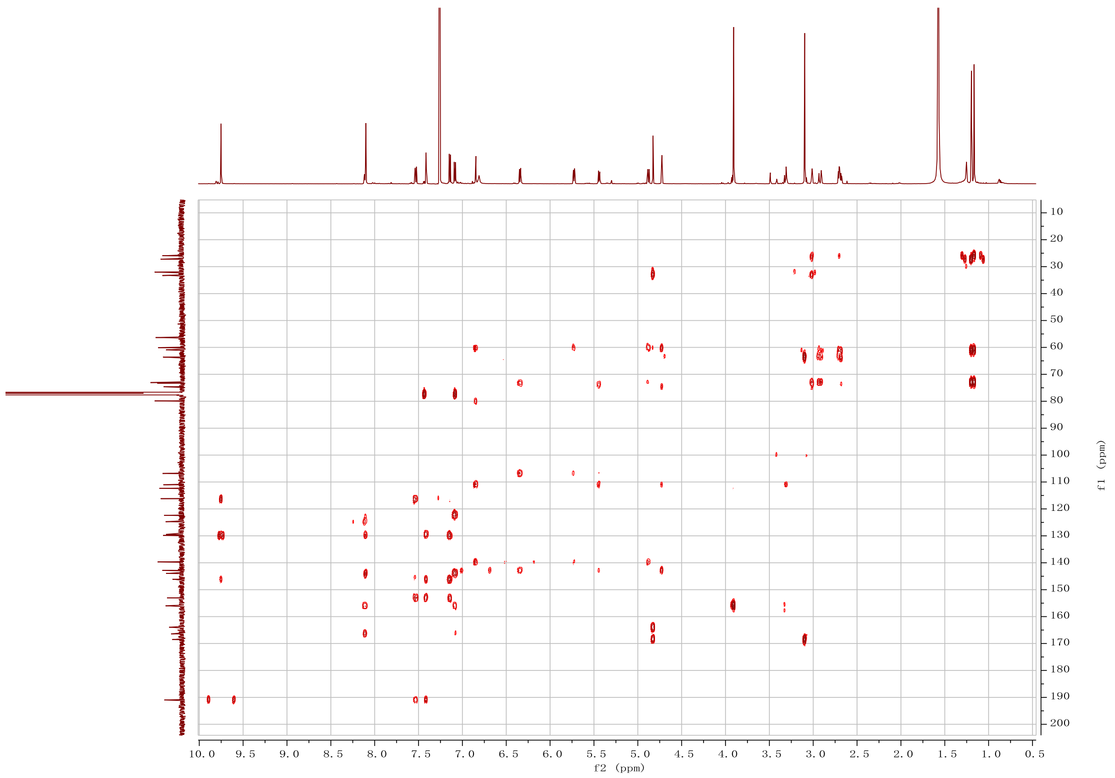


**Figure S47.** HMBC spectrum of **5** recorded in $CDCl_3$

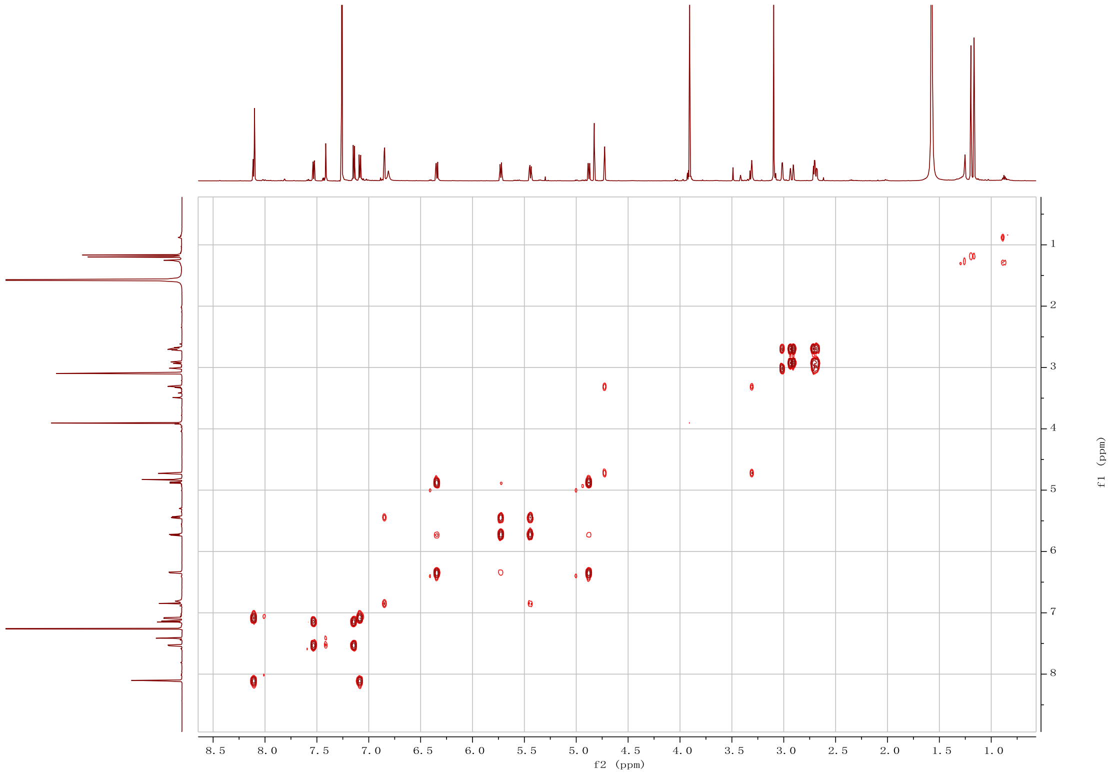


**Figure S48.** $^1H$–$^1H$ COSY spectrum of **5** recorded in $CDCl_3$

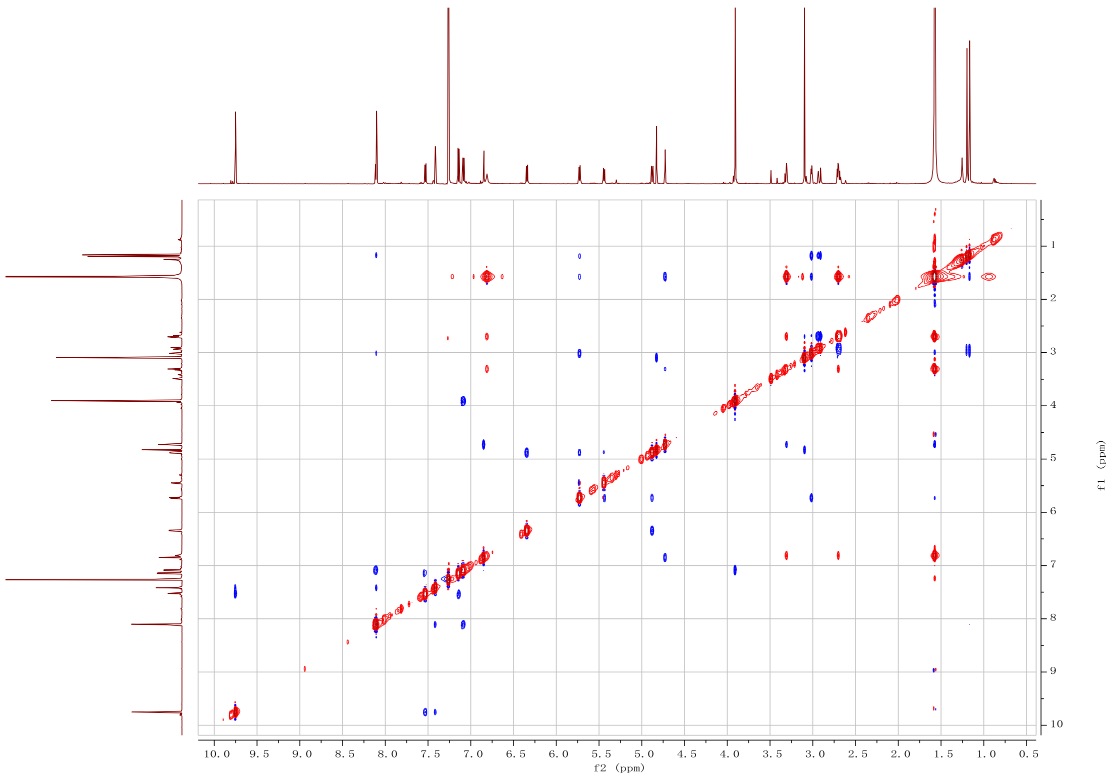


**Figure S49.** NOESY spectrum of **5** recorded in $CDCl_3$

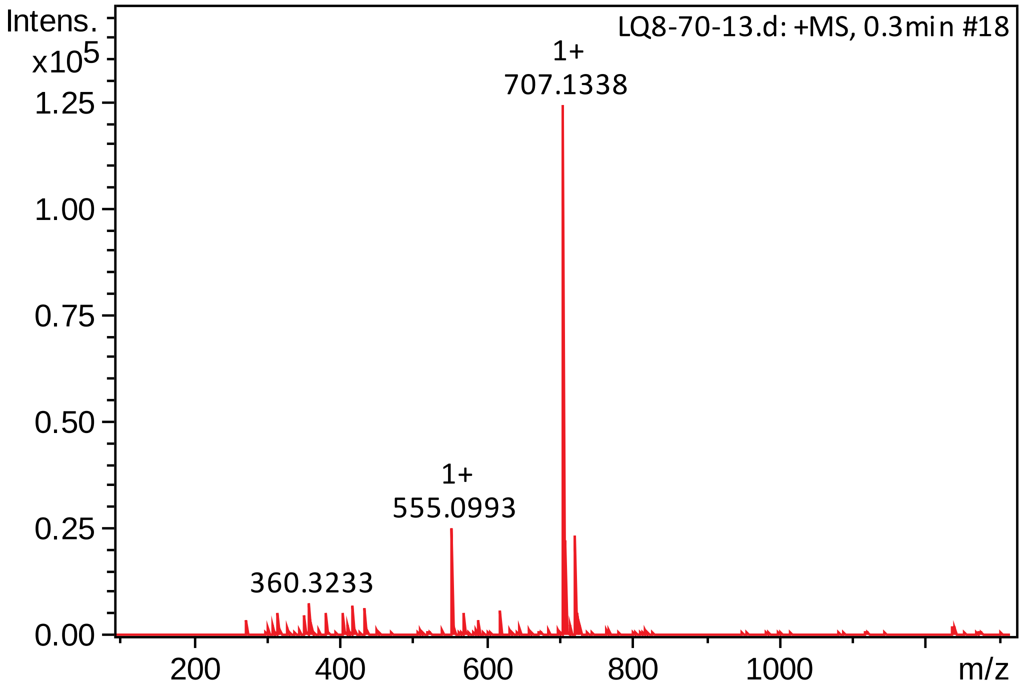


**Figure S50.** HRESIMS spectrum of **5**

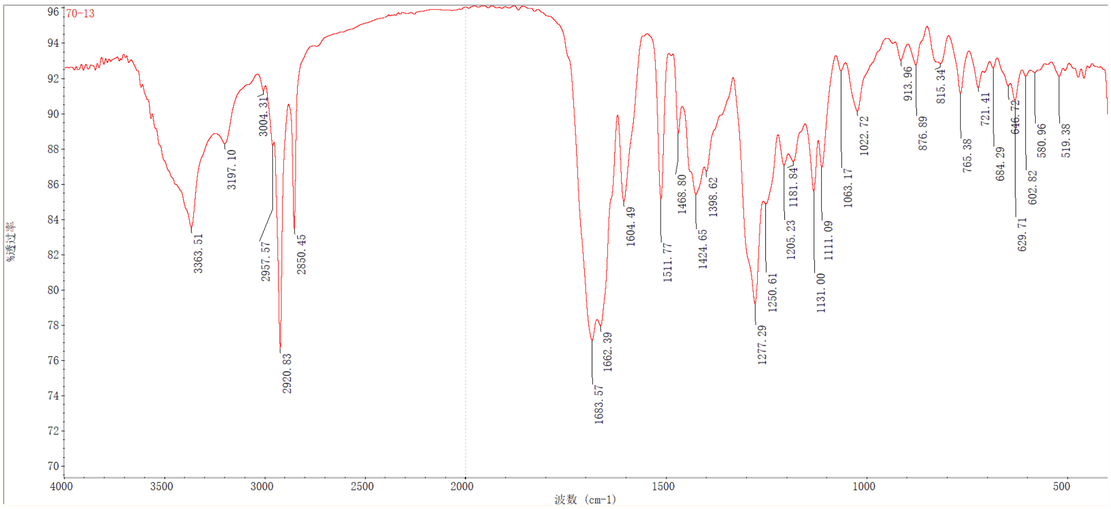


**Figure S51.** IR spectrum of **5**

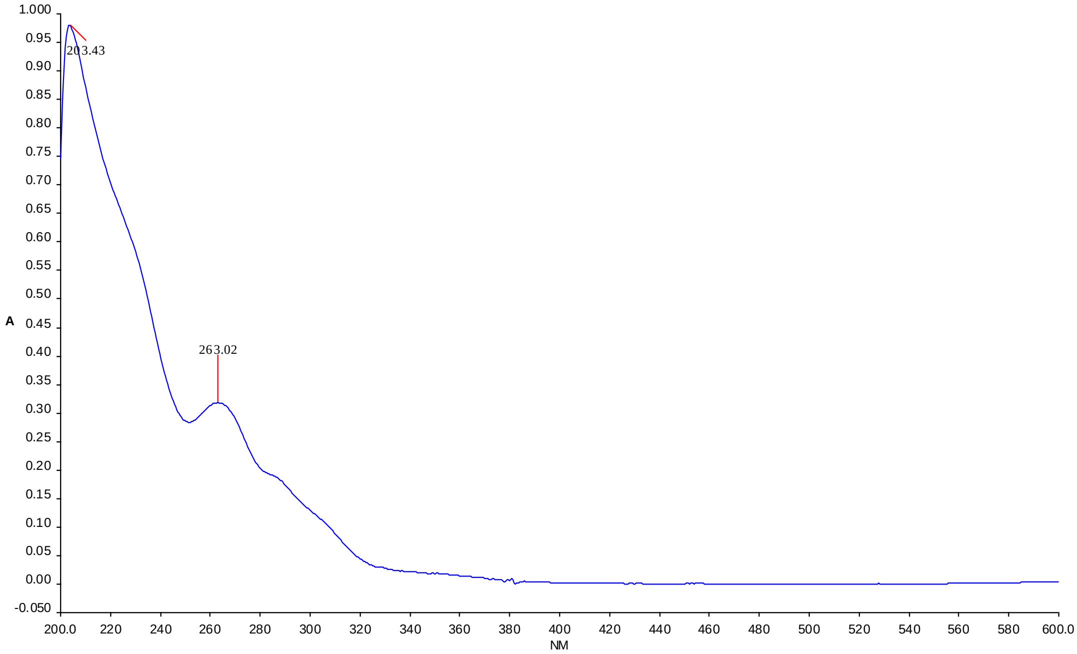


**Figure S52.** UV spectrum of **5**

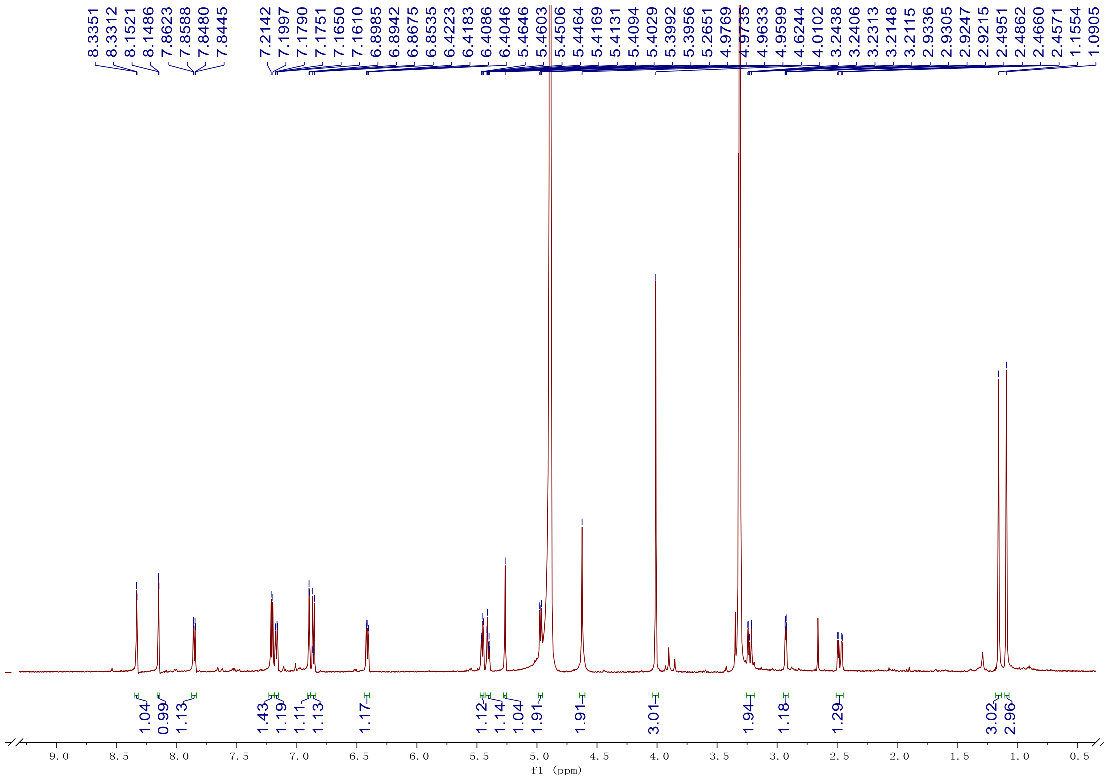

**Figure S53.** $^{1}$H NMR (400 MHz) spectrum of **6** recorded in $CD_3OD$

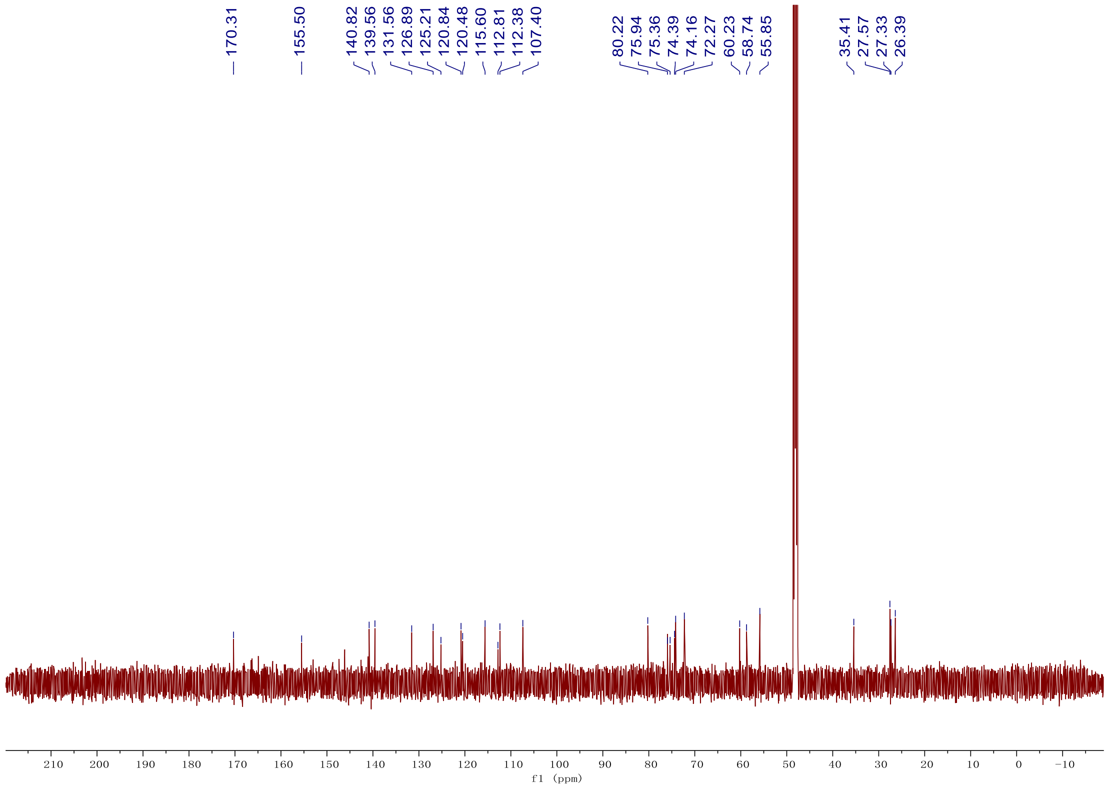

**Figure S54.** $^{13}$C NMR (100 MHz) spectrum of **6** recorded in $CD_3OD$

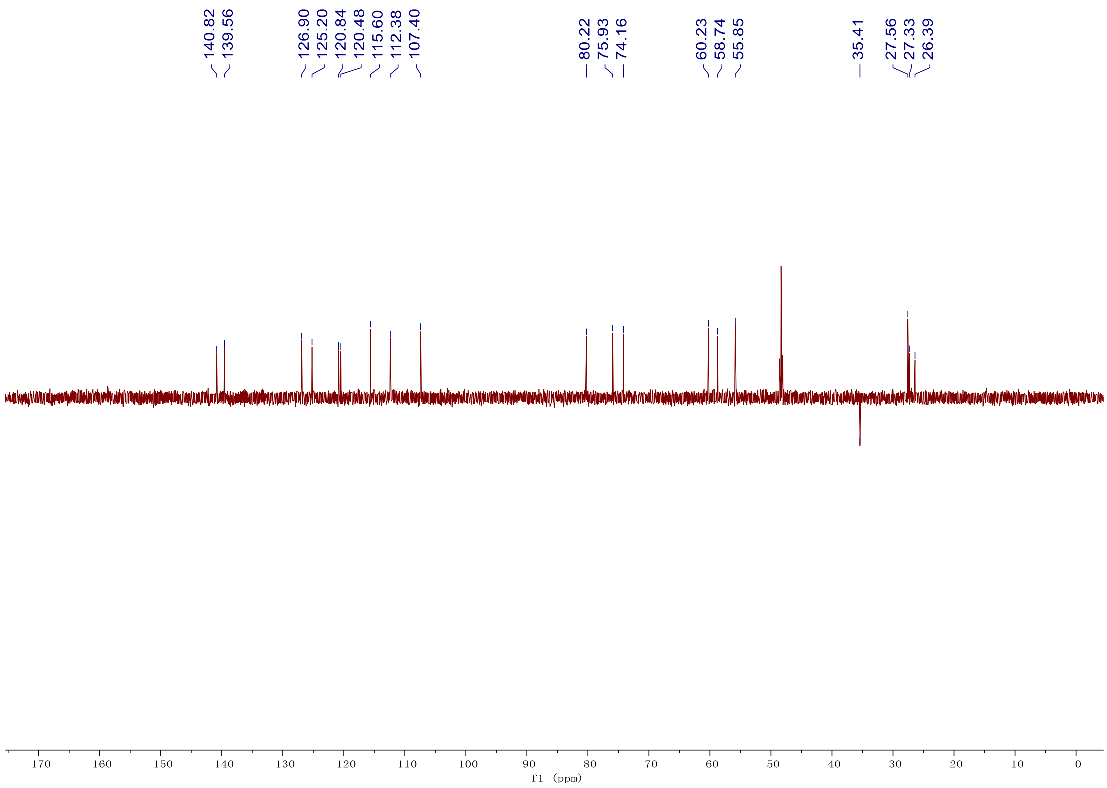

**Figure S55.** DEPT NMR spectrum of **6** recorded in $CD_3OD$

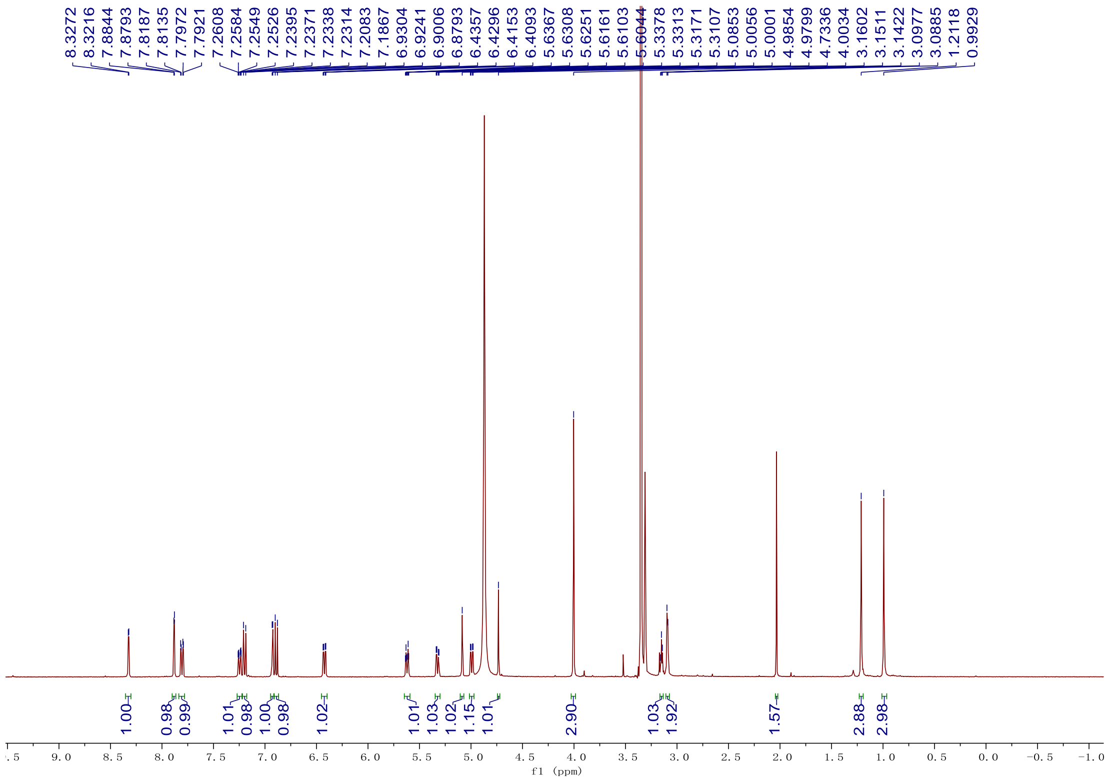

**Figure S56.** $^1H$ NMR (400 MHz) spectrum of **7** recorded in $CD_3OD$

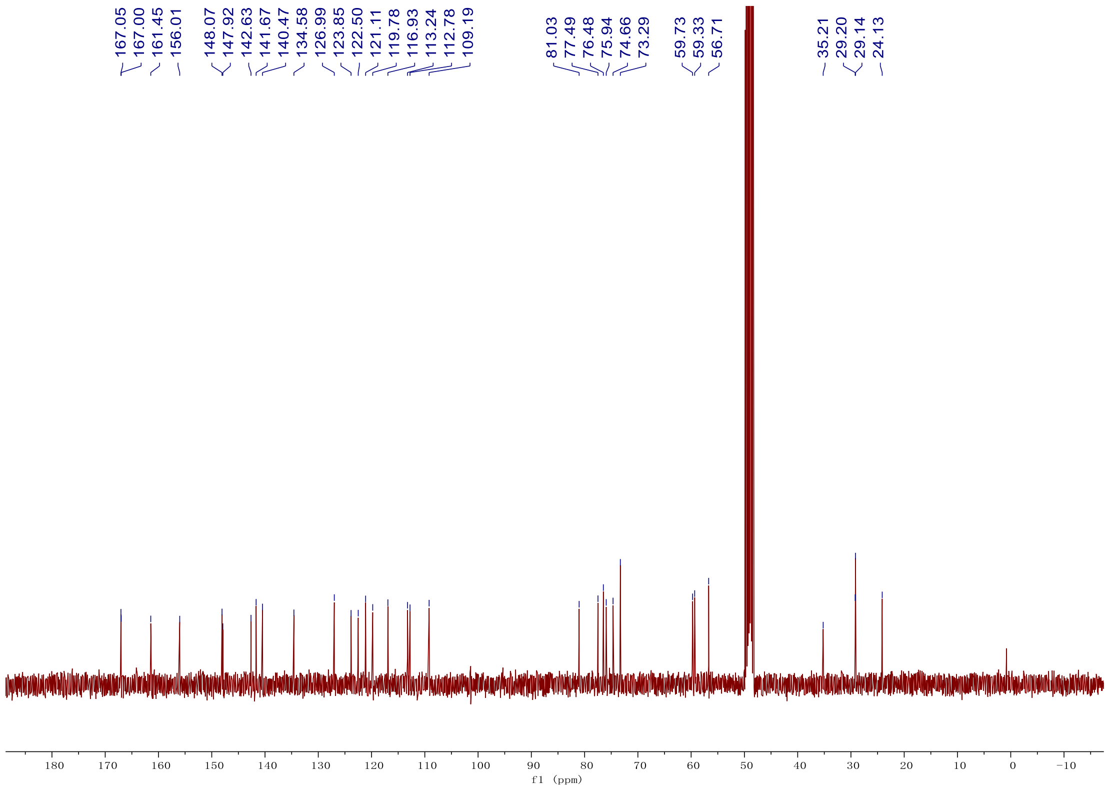


**Figure S57.** $^{13}$C NMR (100 MHz) spectrum of **7** recorded in $CD_3OD$

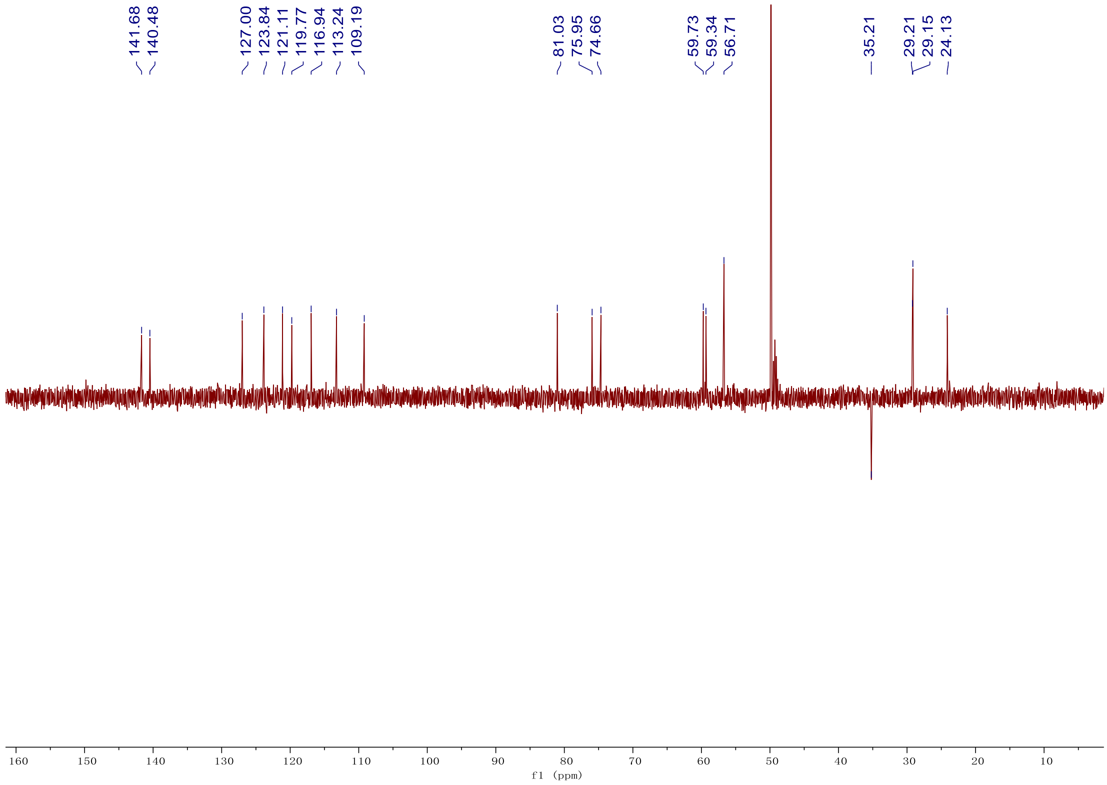


**Figure S58.** DEPT NMR spectrum of **7** recorded in $CD_3OD$
